# Cytidine deaminases catalyze the conversion of *N*(*S*,*O*)^4^-substituted pyrimidine nucleosides

**DOI:** 10.1126/sciadv.ade4361

**Published:** 2023-02-03

**Authors:** Nina Urbelienė, Matas Tiškus, Giedrė Tamulaitienė, Renata Gasparavičiūtė, Ringailė Lapinskaitė, Vykintas Jauniškis, Jurgis Sūdžius, Rita Meškienė, Daiva Tauraitė, Emilija Skrodenytė, Gintaras Urbelis, Justas Vaitekūnas, Rolandas Meškys

**Affiliations:** ^1^ Department of Molecular Microbiology and Biotechnology, Institute of Biochemistry, Life Sciences Center, Vilnius University, Saulėtekio av., 10257 Vilnius, Lithuania.; ^2^ Department of Protein–DNA Interactions, Institute of Biotechnology, Life Sciences Center, Vilnius University, Saulėtekio av. 7, 10257 Vilnius, Lithuania.; ^3^ Department of Organic Chemistry, Center for Physical Sciences and Technology, Akademijos 7, LT-08412 Vilnius, Lithuania.; ^4^ UAB Biomatter Designs (Biomatter), Žirmūnų st. 139A, 09120 Vilnius, Lithuania.

## Abstract

Cytidine deaminases (CDAs) catalyze the hydrolytic deamination of cytidine and 2′-deoxycytidine to uridine and 2′-deoxyuridine. Here, we report that prokaryotic homo-tetrameric CDAs catalyze the nucleophilic substitution at the fourth position of *N*^4^-acyl-cytidines, *N*^4^-alkyl-cytidines, and *N*^4^-alkyloxycarbonyl-cytidines, and *S*^4^-alkylthio-uridines and *O*^4^-alkyl-uridines, converting them to uridine and corresponding amide, amine, carbamate, thiol, or alcohol as leaving groups. The x-ray structure of a metagenomic CDA_F14 and the molecular modeling of the CDAs used in this study show a relationship between the bulkiness of a leaving group and the volume of the binding pocket, which is partly determined by the flexible β3α3 loop of CDAs. We propose that CDAs that are active toward a wide range of substrates participate in salvage and/or catabolism of variously modified pyrimidine nucleosides. This identified promiscuity of CDAs expands the knowledge about the cellular turnover of cytidine derivatives, including the pharmacokinetics of pyrimidine-based prodrugs.

## Teaser

The substrate scope of cytidine deaminases provides insight in the catabolism of modified nucleosides including anticancer ones.

## INTRODUCTION

More than 150 noncanonical nucleosides have been identified in the structures of RNA and DNA (*1*, *2*). Variously modified purines and pyrimidines play important roles in the regulation and structure formation of RNA and DNA molecules, which, in turn, alters their stability and turnover dynamics, transport, and localization (*3*). Numerous enzymes with known and unknown activities are involved in the synthesis of these modifications (*1*, *4*). As de novo synthesis of nucleosides is energetically and nutritionally costly to the cells, the abundant salvage pathways have evolved (*5*). The common enzymes found in these pathways are deaminases, which catalyze deamination of heterocyclic bases, nucleosides, nucleotides, and nucleic acids (*6*). The cytosine deaminases, cytidine deaminases (CDAs), and deoxycytidylate monophosphate deaminases are primarily involved in the salvage of pyrimidines or in their catabolism in prokaryotes and eukaryotes, as well as in bacteriophages. Several enzymes of this group can catalyze deamination of the cytosine moiety of the peptidyl nucleosides such as blasticidin S, hence conferring resistance toward nucleoside antibiotics (*7*, *8*). Another important group of deaminases, APOBEC, catalyze the in situ deamination of bases in both RNA and DNA (*9*, *10*).

Two types of CDAs (EC 3.5.4.5) participating in salvage/catabolism of cytidine and deoxycytidine have been found in nature. The first one consists of homodimeric proteins (D-CDA), for example, CDAs from *Escherichia coli* (*11*), *Arabidopsis thaliana* (*12*), and *Klebsiella pneumoniae* (*13*). The second one is formed by the homotetrameric enzymes (T-CDA) such as CDAs from *Homo sapiens* (*14*) and *Bacillus subtilis* (*15*). Both classes of CDAs contain one catalytic zinc ion per subunit.

In addition to deamination of the primary substrate cytidine or 2′-deoxycytidine, CDAs are involved in the metabolism of nucleoside analogs that are used widely as anticancer (pro)drugs (*16*). Together with carboxylesterase, human CDA converts capecitabine (*17*) into an active compound. However, both human and bacterial CDAs deactivate gemcitabine, cytarabine, azacytidine, and decitabine, hence lowering the efficiency of the drugs. CDA encoded by *cdd* gene from *E. coli* has been shown to catalyze the nucleophilic substitution in bioconversion of *N*^4^-methylcytidine (*18*) and *N*^4^-hydroxycytidine (*19*) to uridine. Analogous reactions are known to be catalyzed by cytokinin deaminase (belongs to amidohydrolase superfamily) on variously *N*^6^-substituted derivatives of adenine (*20*). However, the capabilities of CDAs to catalyze nucleophilic substitution at the fourth position of diversely substituted pyrimidine nucleosides have not yet been thoroughly investigated.

In this study, a functional metagenomic mining using complementation of uridine auxotrophy and *N*^4^-benzoyl-2′-deoxycytidine (**25**) as a source of uridine (*21*) has led to the successful isolation of several CDA homologs. Here, we show that CDAs convert different *N*^4^-acyl-arylpyrimidine, *N*^4^-/*S*^4^-/*O*^4^-alkyl-arylpyrimidine, and *N*^4^-/*S*^4^-/*O*^4^-arylpyrimidine nucleosides, including capecitabine, directly into derivatives of uridine. The solved crystal structure of metagenomic CDA suggests the possible binding mode for bulky modified pyrimidine nucleosides. Bioinformatics and structure-guided mutagenesis allowed identification of the key residues and structural motifs that determine the substrate specificity of CDAs. Collectively, these results suggest a possible involvement of CDAs in various previously unidentified catabolic pathways of modified pyrimidine nucleosides, including the therapeutic ones (*22*).

## RESULTS

### Selection of metagenomic CDAs

**Fig. 1.**
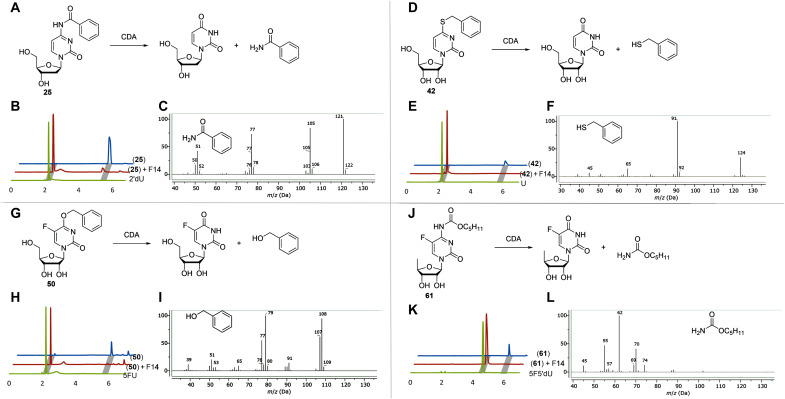
A diversity of reactions catalyzed by CDAs. The conversion of *N*^4^-benzoyl-2′-deoxycytidine (**25**) (**A**), *S*^4^-benzylthiouridine (**42**) (**D**), 4-benzyloxy-5-fluoro-uridine (**50**) (**G**), and capecitabine (**61**) (5′-deoxy-5-fluoro-*N*^4^-pentyloxycarbonylcytidine) (**J**) are shown. High-performance liquid chromatography–mass spectrometry chromatography spectra (254 nm) of reaction products: 2′-deoxyuridine (2′dU) (**B**), uridine (U) (**E**), 5-fluoro-uridine (5FU) (**H**), and 5-fluoro-5′deoxyuridine (545′dU) (**K**), and typical GC-MS chromatography spectra outtakes of extracted reaction products: benzamide (**C**), benzyl mercaptan (**F**), benzyl alcohol (**I**), and penthyl carbamate (**L**) are shown after the reaction schemes, respectively. *m*/*z*, mass/charge ratio.

Three metagenomic clones (EH, F14, and F18) were selected using a previously described method with uridine auxotrophic *E. coli* DH10B Δ*pyrFEC*::Km (*23*) cells and *N*^4^-benzoyl-2′-deoxycytidine (**25**) as a 2′-deoxyuridine (**1**) source (*21*). Unexpectedly, the sequence analysis of the DNA fragments from these clones failed to identify any of the typical amidohydrolase-encoding genes but showed the presence of open reading frames (ORFs) with high 69 to 85% sequence identity to CDAs found in the National Center for Biotechnology Information GenBank database (table S1) (*24*). The individual genes from metagenomic clones EH, F14, and F18 were cloned and recombinantly expressed in *E. coli* HMS174 ∆*pyrF*∆*cdd* cells, and purified enzymes were used to elucidate the catalytic properties of CDAs. All three enzymes (CDA_EH, CDA_F14, and CDA_F18) were active with cytidine (**2**) and 2′-deoxycytidine (**1**), in addition to converting *N*^4^-benzoyl-2′-deoxycytidine (**25**) into 2′-deoxyuridine (**1**) (Fig. 1, A and B, and figs. S1 and S2A). The gas chromatography–mass spectrometry (GC-MS) analysis allowed the identification of benzamide as the leaving group in reactions catalyzed by all three enzymes (Fig. 1C and fig. S2B). Encouraged by these results, we additionally tested several substrates such as *S*^4^-benzylthiouridine (**42**), 4-benzyloxy-5-fluoro-uridine (**45**), and capecitabine (5′-deoxy-5-fluoro-*N*^4^-pentyloxycarbonylcytidine) (**61**) and observed that all of them were fully converted by the selected CDAs (Fig. 1, D, E, G, H, J, and K, and figs. S3A to S5A). Moreover, benzyl mercaptan, benzyl alcohol, and pentyl carbamate were detected as products of hydrolysis catalyzed by CDA_EH, CDA_F14, and CDA_F18 (Fig. 1, F, I, and L, and figs. S3B to S5B). An analysis of kinetic parameters of CDA_F14 showed that both *N*^4^-benzoyl-2′-deoxycytidine (**25**) [The Michaelis-Menten constant ( *K*_M_)(1.15 ± 0.16) × 10^−4^ M, *k*_cat_ (5.04 ± 0.4) × 10^−1^ s^−1^, and *k*_cat_/*K*_M_ (4.36 ± 3.61) × 10^3^ M^−1^ s^−1^] and 2′-deoxycytidine (**1**) [(*K*_M_ 1.95 ± 0.36) × 10^−4^ M, *k*_cat_ (24.4 ± 1.71) × 10^−1^ s^−1^, and *k*_cat_/*K*_M_ (1.25 ± 0.12) × 10^4^ M^−1^ s^−1^] were hydrolyzed with a similar efficiency, and values of *K*_M_, *k*_cat_, and *k*_cat_/*K*_M_ were within a similar range of previously reported kinetic parameters for other CDAs when 2′-deoxycytidine (**1**) was used as a substrate (*25*). On the basis of these results, it was decided to expand functional mining of metagenomic CDAs using *S*^4^-methylthio-uridine (**37**), *S*^4^-ethylthio-uridine (**38**), and *S*^4^-benzylthio-uridine (**42**) as substrates instead of *N*^4^-benzoyl-2′-deoxycytidine (**25**). In total, 27 CDAs (table S1) from 20 metagenomic DNA libraries (table S2) were successfully selected using these compounds.

### Substrate scope of the CDAs

**Fig. 2.**
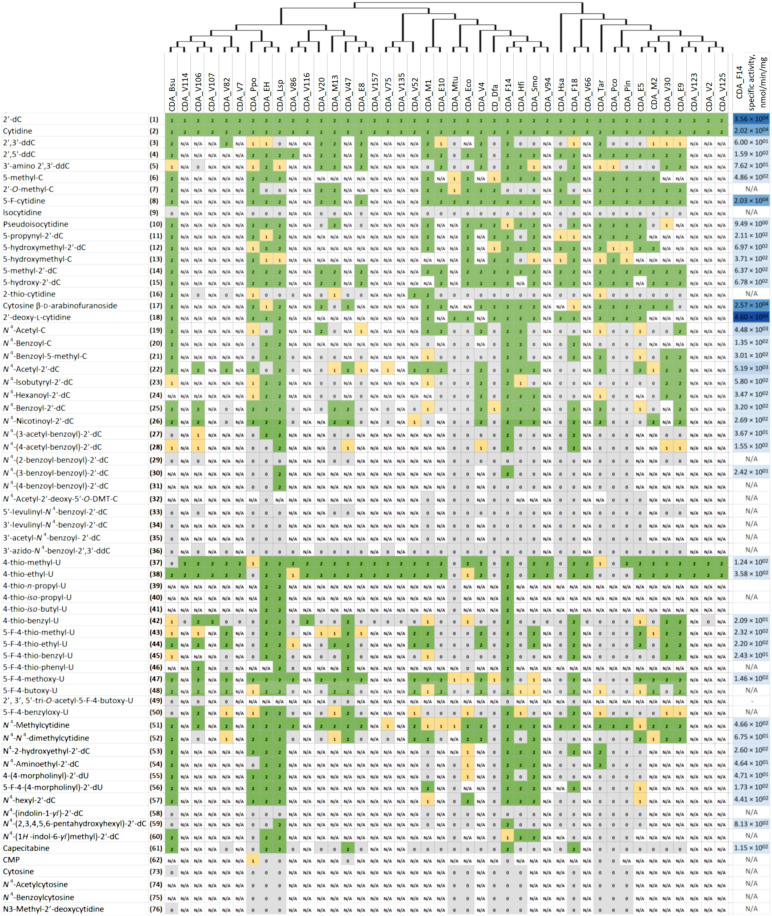
The clustering of the substrate ranges of the CDAs. The list of the tested substrates is shown on the left side (fig. S7). The ID code representing each CDA is given on the top. The phylogenetic tree was added above the ID code at the top of the figure. The phylogenetic analysis of CDAs was conducted using the neighbor-joining tree routine of MEGA X software. The alignment was performed using ClustalW. The activity of enzymes is defined as follows: 2, activity is observed after 3 hours of the incubation at RT; 1, activity is observed after overnight incubation (weakly active); 0, inactive toward the substrate; N/A, not analyzed. The specific activity of CDA_F14 (nanomole per min per milligram) is shown on the right side. Blue color intensity reflects activity. The SD of measurement is shown in the datafile S1.

For the activity analysis, 30 purified recombinant CDAs were chosen: 19 of the most diverse representatives from our selection experiments and 11 homologs from microorganisms found in intestine microbiota (CDA_Hfi, CDA_Lsp, CDA_Smo, CDA_Dfa, CDA_Pco, and CDA_Eco) or in environmental samples (CDA_Ppo, CDA_Bsu, CDA_Pin, CDA_Mtu, and CDA_Tar) (table S1 and fig. S6). The enzymatic activity of remaining 11 metagenomic CDAs (V2, V7, V66, V94, V107, V114, V116, V123, V125, V135, and V157) was determined in vivo only. The substrate preferences of the recombinant enzymes were qualitatively evaluated by thin-layer chromatography (TLC), high-performance liquid chromatography–mass spectrometry (HPLC-MS), and GC-MS using 66 different substrates, including various *N*^4^-unsubstituted cytidines (**1** to **18**, **62**, and **76**), *N*^4^-acyl-pyrimidine (**19** to **36**), *N*^4^-alkyl-pyrimidine (**51** to **60**), 4-alkylthio-pyrimidine (**37** to **46**), and 4-alkoxy-pyrimidine (**47** to **50**) nucleosides, as well as cytosine (**73**) and its derivatives (**74** and **75**) (Fig. 2 and fig. S7). In addition, the specific activity of CDA_F14 toward different substrates was determined (Fig. 2). All 42 CDAs were active with cytidine (**2**) and 2′-deoxycytidine (**1**). Moreover, all tested CDAs deaminated unnatural 2′-deoxy-l-cytidine (**18**) [containing 2-deoxy-l-ribofuranose instead of 2-deoxy-d-ribofuranose in (**1**)]. Analysis of the specific activity of CDA_F14 revealed that derivatives of 2′-deoxycytidine (**1**, **12**, **22**, and **25**) were hydrolyzed slightly more efficiently than the corresponding ribonucleosides (**2**, **13**, **19**, and **20**) (Fig. 2). Some modifications on ribose [such as 3′-amino-3′-deoxy- (**5**) or 2′-*O*-methyl- (**7**)] were moderately accepted by only a few enzymes. However, the presence of azido group (**36**), an acylation of ribose of 4-acyl/alkyl (2′-deoxy)-cytidines (**32** to **35** and **49**) or phosphorylation of cytidine (**62**) had detrimental effect on activity of all CDAs. The enzymes well tolerated various substituents at the fifth position of the heterocyclic base including *N*^4^-unsubstituted (**6**, **8**, and **11** to **15**) and *N*^4^-substituted substrates (**21**, **43 to 48**, **50**, and **56**); however, only few of the CDAs (CDA_Bsu, CDA_M1, and CDA_V52) deaminated 2-thiocytidine (**16**) efficiently. However, it should be noted that alkyl groups at the fifth position of heterocyclic bases reduced the activity of the enzyme CDA_F14 quite strongly. None of the CDA’s tested catalyzed the hydrolytic deamination of cytosine (**73**) or *N*^4^-substituted cytosine (**74** and **75**) analogs, N^3^-methyl-2′-deoxycytidine (**76**), and isocytidine (**9**). However, pseudoisocytidine (**10**) was a substrate for many of tested deaminases (16 of the tested 22 CDAs). On the basis of activity profiles with substrates containing different substituents at the fourth position of pyrimidine ring, CDAs distributed into two groups: The first group consisted of the enzymes CDA_Mtu, CDA_Hsa, CDA_Eco, CDA_Pco, CDA_Pin, and CDA_Dfa, which only used methylated *O*-, *S*-, and *N*-derivatives (**37**, **43**, **47**, and **51**), while the second group consisted of CDAs active with both methylated and bulky aliphatic or aromatic substituents harboring nucleosides (**19** to **61**). The substrate specificity of the individual CDAs from the second group was highly variable. CDA_Lsp, CDA_F14, and CDA_EH exhibited the widest range of substrates converting 49 (82%), 49 (79%), and 43 (72%) of 66 tested compounds, respectively. CDA_V47 converted 19 compounds of 27 tested compounds (73%) with bulky substituents at the fourth position. CDA_Bsu and CDA_F18 enzymes were next in order of substrate recognition, converted 35 of 53 (66%) and 31 of 57 (54%) derivatives, respectively. Furthermore, CDA_Lsp, CDA_F14, and CDA_EH enzymes showed a regioselectivity toward *N*^4^-(acetyl-benzoyl)-/*N*^4^-benzoyl-benzoyl-/-2′-deoxycytidine (**27** to **31**) isomers (Fig. 2). A comparison of specific activities of CDA_F14 toward 4-unsubstituted and 4-substituted pyrimidine nucleosides revealed that latter cytidine derivatives were converted less efficiently (**1**, **2**, **8**, **17**, and **18**). Moreover, the specific activity decreased with increasing the 4-substituent group. The nature of heteroatom at the fourth position had only a moderate impact on the activity of CDAs (**37**, **43**, **47**, and **51**).

### Prevalence of substrate promiscuity in CDAs

**Fig. 3.**
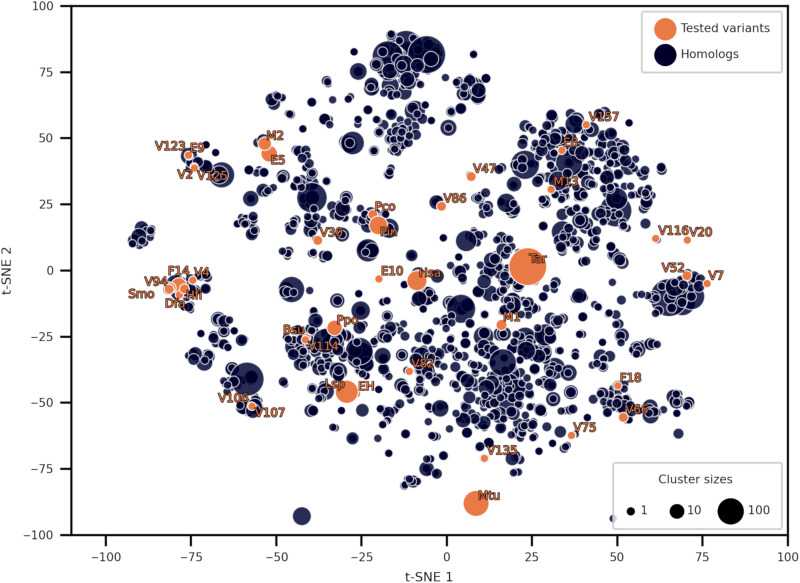
t-SNE representation of the CDA close homolog sequence space. Coordinates of t-SNE embedding of 1708 cluster representatives were used for plotting. The size of a given dot was visualized on the basis of the cluster size it represents and coral orange color with label if the cluster contained a tested CDA variant.

A phylogenetic analysis of selected CDAs was conducted to assess a possible relationship between specificity toward substrates and amino acid sequences (Fig. 2). The sequences were split into three branches but did not show grouping according to the specificity for the 4-substituted nucleosides. In addition, the sequences were compared to each other in the context of close CDA homolog sequence space. The homologous CDA sequences were collected by searching the tested variants against the UniRef100 (*26*) database. The resulting CDA hits were filtered to include only closer homologs (>50% sequence identity) and clustered at 70% sequence identity threshold to obtain 1708 clusters. The cluster representative sequence dissimilarity matrix was embedded into a two-dimensional (2D) graph using *t*-distributed stochastic neighbor embedding (t-SNE) (*27*). However, the embedding observed in the t-SNE plot did not explain the specificity profiles of different CDA homologs, as their distances in the embedding did not correspond to differences in specificity (Fig. 3).

The multiple sequence alignment of the selected CDAs (fig. S8) confirmed four conservative regions (*15*). The conservative VGA site (region I) in the N terminus was found in most of the analyzed CDAs. However, other variations including VAC, VSC, IGA, and VAA could be detected in this region (*15*). Only CDA_V20 from the examined CDAs contained VGCA region characteristic for *Saccharomyces cerevisiae* CDA (*15*) and CDA_M1 contained the VGV region characteristic for *Mycobacterium leprae* (*15*). The NXEN(S) motif (region II) in the N terminus was also identified in all studied proteins. The NXES sequence found in CDA_V66 and CDA_V116 sequences was characteristic for CDA from a parasitic roundworm *Brugia pahangi* (*15*). Other variations including NXEC or NXEXX also could be detected in this region (*15*). Both of the mentioned N terminus motifs proposedly support the tetrameric structure of the enzyme. The motif C(A/G)E(R/C/T)X [X, polar uncharged amino acids (Ser, Thr, and Asn) or small hydrophobic amino acid (Ala and Val)] (region III) included the zinc-coordinating residue Cys^53^ (here and in the rest of this article, a numeration was according the CDA_F14) and Glu^55^ that is essential for catalysis. Three CDAs (CDA_E5, CDA_Mtu, and CDA_V86) instead of a conservative Arg^56^ contained His, Cys, and Thr, respectively. The fourth conserved region consisted of the sequence PCXXCRQ (region IV), including two cysteines Cys^88^ and Cys^91^ (with the exception in the case of CDA_E10, where PCXXCLQ was found), which coordinate the zinc atom. The highly conservative Glu/Asp^97^, Pro^123^, and Phe^126^ were found in the C termini of CDAs. The overall pairwise identity of 42 tested CDAs sequences was 35%. The visible difference between the sequences was a slightly prolonged N terminus in several CDA: Pin, Pco, E5, M2, M3, E9, E12, V2, V30, V123, and V125. Exclusively, CDA_V86 had a prolonged middle part of the protein uncharacteristic for other CDAs. However, the functional relevance of these regions remains unclear.

### Crystal structure and molecular modeling of CDAs

**Fig. 4.**
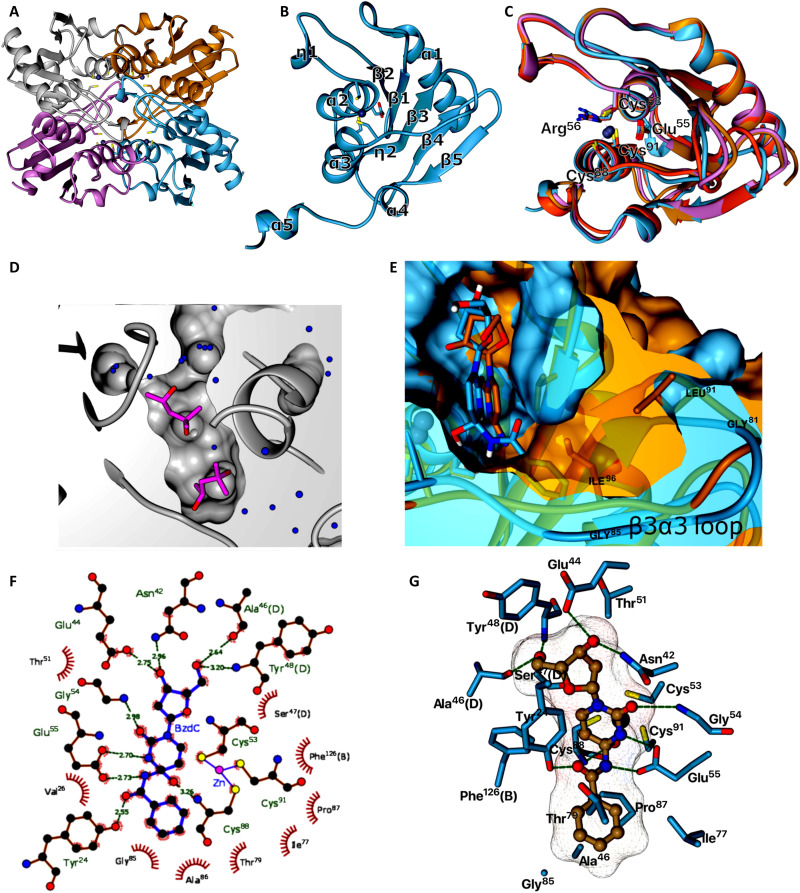
The crystal structure of CDA_F14. (**A**) Homotetramer of CDA_F14 with subunits colored in gray, gold, blue, and violet. (**B**) Subunit of CDA_F14 consisted of a core of five β strands (β1 to β5) sandwiched by five α helices (α1 to α5), and η1 and η2 symbols indicate 3_10_-helix; zinc atom (blue sphere) is coordinated by three cysteine residues located in α helices α2 (Cys^53^) and α3 (Cys^91^ and Cys^88^). (**C**) Comparison of CDA domain structures from different organisms: CDA_F14 is colored in blue, CDA_Mmu (PDB 2FR6) in gold, CDA_Bsu (PDB 1JTK) in coral, and CDA_Hsa (PDB 1MQ0) in violet. (**D**) Active site of CDA_F14 occupied by two molecules of crystallization agent 2-methyl-2,4-pentanediol (in purple); water molecules are shown as blue spheres. (**E**) Comparison of substrate binding pocket in CDA_F14 (blue) and in CDA_Mmu (gold); *N*^4^-benzoyl-2′-deoxycytidine and 2′-deoxycytidine are fitted in the active center, and the β3α3 loop is shown in front. (**F** and **G**) 2D and 3D schematic view of the active site of crystalized CDA_F14 with fitted *N*^4^-benzoyl-2′-deoxycytidine. The bond lengths are given in angstroms. The bond length between zinc and 4-hydroxyl group was 1.94 Å. Hydrogen and coordination bonds are shown as dotted lines. 2D view generated using LigPlot+ program (*62*), other views with Chimera1.16 (*53*).

CDA_F14 was chosen as the crystallization object because of its wide substrate specificity. The crystal structure of CDA_F14 [Protein Data Bank (PDB) 7ZOB] was resolved at 1.2-Å resolution (table S3). Asymmetric unit contained eight CDA_F14 subunits forming tetramers (Fig. 4A). Each subunit of the CDA_F14 tetramer consisted of a core of five β strands (β1 to β5) sandwiched by five α helices (α1 to α5) and exhibited a fold characteristic of the CDA family (Fig. 4, A and B). Structural comparison of CDA_F14 with CDA_Bsu, CDA_Hsa, and CDA_Mmu showed that all proteins had a similar core of α/β/α deaminase domain (Fig. 4C). As described earlier (*10*), three subunits were involved in the formation of the active center. In the crystal structure, the active site was occupied by two molecules of 2-methyl-2,4-pentanediol, which was used as a crystallization agent (Fig. 4D). Multiple attempts to obtain a crystal structure of substrate-bound CDA_F14 were unsuccessful. Therefore, amino acid residues, which are in close contact with substrate, were identified using molecular docking program Autodock Vina (*28*). *N*^4^-benzoyl-2′-deoxycytidine (**25**) was docked into the determined structure of CDA_F14, and the following interacting residues of amino acids were found: Glu^55^, Asn^42^, Glu^44^, Gly^54^, Cys^88^, Tyr^24^, Ala^46^(D), and Tyr^48^(D) (Fig. 4, F and G, and table S4). All these amino acids were highly conserved between CDAs (fig. S8). The substrate binding was mediated by the hydrogen bonds between oxygen atoms at 3′- and 5′-positions of ribose ring and Asn^42^, Glu^44^, Ala^46^(D), and Tyr^48^(D); among C2 position of the heterocyclic base and Gly^54^; and one at *N*^4^-acyl group and Tyr^24^. The hydrophobic substrate binding pocket area of CDA_F14 (distance to the docked substrate shorter than 3.9 Å) was formed by Val^26^, Ile^77^, Thr^79^, Gly^85^, Ala^86^, Pro^87^, Leu^107^, Phe^126^(B), and Leu^131^(B) (Fig. 4, F and G). Phe^126^ is a highly conservative amino acid in CDAs and is important for arrangement of the active site (*15*). The amino acids supporting the tetrameric structure by the formation of hydrogen bonds are presented in fig. S9.

The catalytic zinc ion in CDA_F14 was coordinated by three cysteine residues located in α helices α2 (Cys^53^) and α3 (Cys^91^ and Cys^88^). While in the dimeric CDA_Eco Zn^2+^ is coordinated by two Cys and one His residue. Accordingly, mono-Cys/His (F14_C53H_R56Q), double-Cys/His (F14_C88H_C91H), and triple-Cys/His (F14_HQHH) mutants were constructed to test the interchangeability of these cysteines. The amino acid residue Arg^56^ in mono-Cys/His and triple-Cys/His mutants was changed to Gln by the analogy to CDA_Eco. Activity analysis showed that the double- and triple-Cys mutants were completely inactive and only the mono-Cys/His (F14_C53H_R56Q) mutant retained some activity toward 2′-deoxycytidine (**1**) (fig. S10). In addition, CDA_F14 was completely inactive in the presence of 10 mM EDTA. To assess the influence of other amino acid residues on CDA activity, the β3α3 loop, C-terminal amino acids (amino acids 126 to 130), Thr^51^, and Arg^56^ were selected for mutagenesis.

### Influence of amino acids in the β3α3 loop on CDA selectivity

We hypothesized that the size of the substrate binding pocket should determine activity toward the substrates with bulky *N*(*O*,*S*)^4^-substituents. To that end, we compared the published crystal structures of CDAs (PDB IDs 2FR6, 2FR5, 1ZAB, 1R5T, 1MQ0, 3IJF, and 1JTK) and CDA_F14. The β3α3 loop (amino acids 79 to 88) of CDA_F14, connecting the third β strand to the fourth α helix, was identified as most likely to influence the space for binding of *N*^4^-substituted nucleosides. The comparison of β3α3 loops in CDA_F14 and CDA_Mmu (PDB ID 2FR6) showed that the aliphatic Ile^96^ and Leu^91^ residues in CDA_Mmu restricted the space near the catalytic amino residues. The CDA_F14 had Gly^81^ and Gly^85^ at the corresponding positions (Fig. 4E); consequently, the binding pocket of CDA_F14 was larger than that in CDA_Mmu (Fig. 4E). The conservative Phe^126^ (from other subunit) restricted space in the binding pocket from opposite side of β3α3 loop. Hence, the distance between Gly^85^(C) and Phe^126^(CE1) was 7.24 Å in CDA_F14. The interspace between the corresponding position in CDA_Bsu and CDA_Mmu was shorter: 4.45 Å [Phe^125^ (CE1) → Val^83^(CG2)] and to 3.73 Å [Phe^137^(CE1) → Ile^96^(CD1)], respectively. The distance (8.55 Å) between Phe^126^ and Gly^81^ was also the longest in CDA_F14 compared to 4.55 Å in CDA_Bsu [Phe^125^(CE1) → Thr^79^(CG2)] and 5.69 Å in CDA_Mmu [Phe^137^(CE1) → Leu^91^(CD2)]. In particular, the distance between C4 on benzoyl residue and C4 on cytidine pyrimidine ring is equal 6.55 Å in the *N*^4^-benzoyl-2′-deoxycytidine (**25**). So, the length of the *N*^4^-acyl in this substrate exceeded the gap between aforementioned amino acids in CDA_Bsu and CDA_Mmu. Moreover, according to the created model, the benzoyl group should be bend by ~39° with respect to the pyrimidine ring (Fig. 4E). In this configuration, the C4 atom of pyrimidine ring is arranged at the required distance from Glu^55^ that acts as a proton shuttle. The Glu^55^ transfers proton to *N*^3^, which promotes hydroxide attack on C4 position, forming tetrahedral intermediate (*29*). The proper configuration becomes impossible when positions 81 and 85 contain long aliphatic amino acids. Reviewing amino acid sequences in tested CDAs, various combinations of the amino acid residues were observed in β3α3 loop (fig. S8), but the motif 81GXXXG(A)85 was dominating in CDAs that were active toward cytidine with bulky substituents (CDA: F14, V47, Hfi, Lsp, and Smo) (fig. S8).

In addition, the binding pocket volumes were calculated and Pearson correlation between the binding pocket solvent-accessible surface area (SASA), and the substrate volume was examined (fig. S11). To evaluate a relationship between the geometry of active site of CDAs and substrate specificity, the 3D structures of CDAs were predicted by using two modeling methods: AlphaFold2 (*30*) (except CDA_F14 and CDA_Bsu, for which the PDB data were used) (fig. S11A) and HHpred bioinformatics (*31*) toolkit MODELLER by using CDA_F14 crystal structure as a template (fig. S11B). Comparison of 12 different CDAs showed that CDA_F14 had the largest binding pocket among them, but no direct correlation between the calculated substrate volume and the binding pocket size of CDAs was found.

**Fig. 5.**
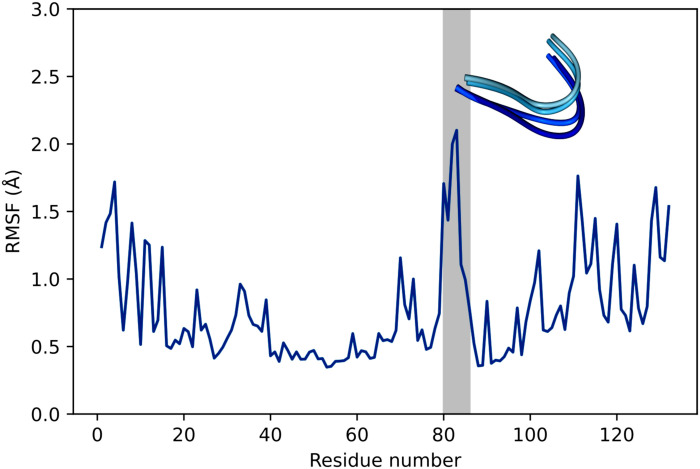
The flexibility of amino acids residues in CDA_F14: *N*^4^-benzoyl-2′-deoxycytidine complex. Root mean square fluctuation value (RMSF) measurements represent individual residue flexibility during a 100-ns simulation. The loop 80-to-86 region is highlighted in gray, and the several conformations for the loop are shown. The molecular dynamics trajectories were generated by using CPPTRAJ program from the AmberTools package.

**Fig. 6.**
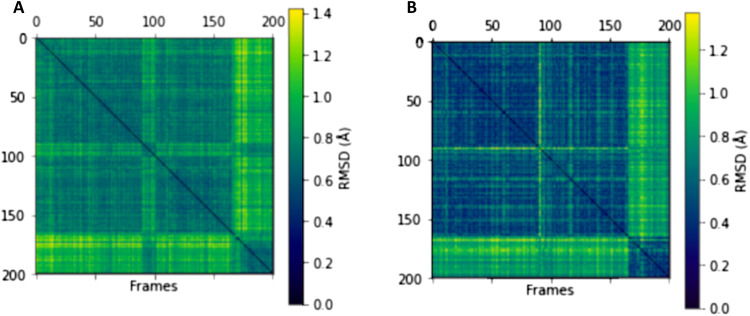
2DRMSD plots for the protein structure during a 100 ns simulation of the CDA_F14: N4-benzoyl-2′-deoxycytidine complex. (**A**) 2D root mean squared deviation (2DRMSD) plot for the whole protein structure and (**B**) 2DRMSD plot only for the loop 80-to-86 atoms. Two conformations during the simulation can be observed for the whole protein, especially for the 80-to-86 loop region.

The mismatch between structure and functional activity could be explained by the mobility of the β3α3 loop. Molecular dynamics simulation data (Figs. 5 and 6) and B factor of the crystal structure indicated the mobility of the β3α3 loop fragment formed by the 80th to 85th amino acids. The overall average B factor of CDA_F14 was about 17.57, while B factor of the 80-to-83 site was 25.38. In addition, the fluctuations of this region during MD simulations of the CDA from *Mycobacterium tuberculosis* was described (*32*), but the impact of this region to substrate binding was not previously analyzed.

Mutagenesis of the amino acids in the β3α3 loop of CDA_F14 revealed the following results: The G81L mutation reduced the *k*_cat_/*K*_M_ values for *N*^4^-benzoyl-2′-deoxycitidine (**25**) by about fivefold, the G85L mutation by 20-fold, and the double-F14_G81LG85I mutation by more than 50-fold (table S5 and fig. S12). Deletion of the 83-to-85 site (F14_del83-85) almost completely inactivated the enzyme; the activity toward cytidine was observed only after a prolonged incubation. However, analysis of the activity of the F14_SML (83to 85) and F14_HSL (83 to 85) mutants showed that leucine at the 85th position did not alter the selectivity for cytidine nucleosides with aromatic substitutes at *N*^4^ position (kinetic parameters were not evaluated).

The effect of mutations in the β3α3 loop on enzyme selectivity was tested with other CDAs. As a consequence, the Lsp_A82I mutant lost activity toward several *N*^4^-substituted cytidines with bulky acyl and aryl residues (**26** to **27**, **31**, **43** to **47**, **55**, and **59**) (fig. S10). In contrast, the Tar_I85A mutant became active toward 5-fluoro-4-(4-morpholinyl)-2′-deoxyuridine (**56**) and *N*^4^-[(1H-indol-6-yl)methyl]-2′-deoxycytidine (**60**) (fig. S10). However, the Pco_I108A mutant remained inactive toward N4-substituted cytidine (**19** to **36**), whereas the activity of the Ppo_V82L mutant was not substantially changed (fig. S10).

### Impact of the C-terminal amino acids to selectivity of CDAs

The function of C-terminal amino acids was evaluated by mutagenesis of the conservative Phe^126^ (F14_F126A and F126W mutants) and the deletion (F14_del127-130) or random changing (F14_HSSG and F14_CLYR) amino acids at positions 127 to 130. Compared to the wild-type CDA_F14, the *K*_M_ of both Phe^126^ mutants increased approximately twofold (table S5). The F126A mutation had a slightly stronger effect on substrate binding compared to F126W [*K*_M_ (2.59 ± 0.73) × 10^−4^ M and (2.11 ± 0.38) × 10^−4^ M, respectively]. This may be related to the loss of π-π stacking. Deletion of amino acids at positions 127 to 130 (F14_del127-130) did not substantially affect the *K*_M_ and substrate profiles (fig. S10) but markedly reduced *k*_cat_/*K*_M_ (table S5). In addition, changes in the C-terminal amino acids of CDA_F14 resulted in activity toward 5′-monophosphorylated nucleotides. Hence, the mutants F14_del127-130, F14_F126A, and F14_F126W began to use CMP as a substrate; however, F14_HSSG and F14_CLYR retained their wild-type specificity (fig. S10). It should be noted that the activity toward CMP nucleotide was extremely low, and a partial hydrolysis was only observed after a prolonged incubation [>18 hours, at room temperature (RT)].

### Impact of the Thr^51^ and Arg^56^ on selectivity of CDAs

On the basis of simulation data, the Thr^51^ position may affect the binding of the ribose ring. The kinetic parameters of the F14_T51G mutant were not qualitatively evaluated, because of a lower rate of the catalyzed reaction and higher *K*_M_ required substrate concentrations that were outside the solubility range and/or a linear range of the spectrophotometer (fig. S12). The substrate scope analysis indicated that T51G mutation affects selectivity. The F14_T51G mutant lost activity toward compounds **3**, **25**, **27** to **28**, **42**, **55**, **59**, and **61** (fig. S12). However, a new weak activity toward CMP (**62**) was observed. Notably, activity toward CMP was also observed for the mutant Ppo_C50T but not for Pco_G70T (fig. S12).

The change of arginine to leucine at the 56th position affected a catalytic efficiency of the constructed mutant F14_R56L: *K*_M_ constant changed slightly compare to wild-type CDA_F14, but kinetic efficiency (*k*_cat_/*K*_M_) of the enzyme decreased almost 50-fold (table S5).

## DISCUSSION

**Fig. 7.**
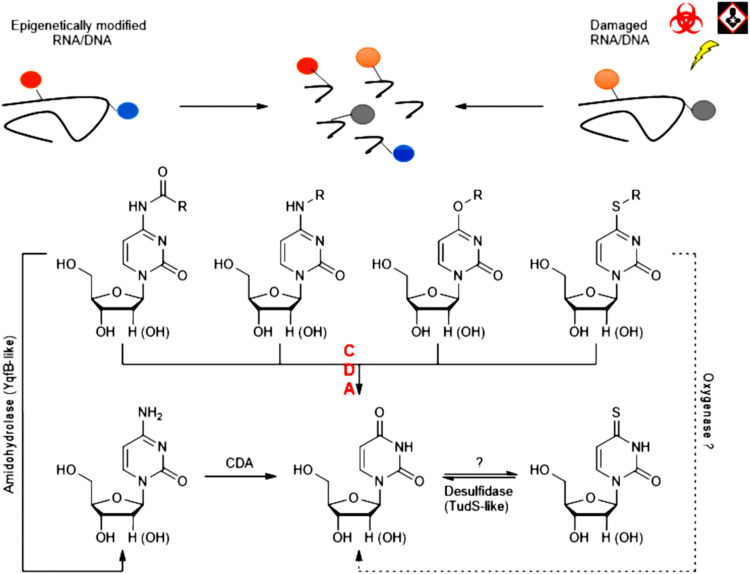
A putative role of CDAs in a catabolism of the modified pyrimidine nucleosides. The noncanonical nucleobases originate due to various epigenetic events, stress responses, or during action of mutagens. This research shows that various CDAs can catalyze the nucleophilic substitution at the fourth position of the heterocyclic ring of *N*^4^-acyl-cytidines, *N*^4^-alkyl-cytidines, *S*^4^-alkylthio-uridine, and *O*^4^-alkyl-uridine derivatives subsequently, leading to the formation of uridine and respective amide, amine, carbamate, thiol, or alcohol.

The noncanonical nucleobases [for example, 4-methylthiouridine (*33*), *N*^4^-methyl cytosine in DNA (*4*), and *N*^4^-acetylcytosine in RNA (*1*)] occur as a result of a variety of epigenetic events, transfer RNA (tRNA) modifications, stress responses, or mutagenic exposures (Fig. 7). However, the subsequent fate of the modified nucleotides, especially after breakdown of nucleic acids, is not yet fully understood.

This research shows that various CDAs can catalyze the nucleophilic substitution at the fourth position of the heterocyclic ring of *N*^4^-acyl-cytidines (**19** to **36**), *N*^4^-alkyl-cytidines (**51** to **60**), *N*^4^-alkyloxycarbonyl-cytidines (**61**), *S*^4^-alkylthio-uridine (**37** to **46**), and *O*^4^-alkyl-uridine (**47** to **50**) derivatives subsequently leading to the formation of uridine and respective amide, amine, carbamate, thiol, or alcohol. Hence, the catabolism of 4-methylthiouridine, which is present in small amounts in cells under stress (*34*), can possibly be attributed to the activity of CDAs determined in this study. Moreover, CDAs can be involved in the degradation of *N*^4^-acetylcytidine, a nucleotide found in tRNAs, mRNAs, and ribosomal RNAs (*1*). Recently, the amidohydrolase YqfB from *E. coli* has been identified, which is active toward *N*^4^-acetylcytidine (*35*). It can be speculated that in some organisms, CDAs act individually or in concert with YqfB-like enzymes to salvage these nucleosides. Catabolism of *O*^4^-alkyl derivatives of pyrimidines, which can be formed by reacting with mutagens (*36*), is also an incomplete story. *O*^4^-alkyl lesions in DNA can be repaired by *O*^6^-alkylguanine DNA alkyltransferases (AGTs). AGTs perform a direct covalent transfer of an alkyl group from the damaged base to a nucleophilic cysteine residue present in the active site of the enzyme, inactivating AGT and restoring DNA. AGTs are specialized to repair the alkylation occurring at the *O*^6^-position of 2′-deoxyguanosine and, to a variable extent, the *O*^4^-position of thymidine (*37*). However, enzymes active toward O-alkylated ribonucleotides are unknown. On the basis of the results of this study, it can be proposed that CDAs could take part in a catabolism of O-alkylated pyrimidines converting them into uridines.

In addition to natural nucleosides, a plethora of 5-fluoropyrimidines, used for cancer treatment, interacts with the human microbiota. Capecitabine, a prodrug for 5-fluorouracil (*17*), is an oral antimetabolite chemotherapeutic agent, which can be metabolized by gut microbiota (*38*). It is known that microorganisms participate in the late stages of catabolism of capecitabine (**61**), for example, in deglycosylation of 5′-deoxy-5-fluorouridine (*39*). Our study shows that prokaryotic CDAs can convert capecitabine directly to 5′-deoxy-5-fluorouridine (Fig. 1, J to L, and fig. S5) without the involvement of any human esterase, as generally assumed. This “off-target” formation of active drug in the intestine, which can affect the composition of intestinal microbiota, plays a role in cancer outcomes and anticancer response (*39*). The further research of CDAs and its effects on antimetabolite drugs could help to personalize a treatment and to improve the favorable clinical outcomes.

According to the results of this study, the selectivity of the CDAs toward cytidines can be very diverse. The profiles of activity toward substrates with different substitutes at fourth position of pyrimidine ring show that CDAs are distributed into two groups: The first one includes enzymes, which used methylated *O*-, *S*-, and *N*-derivatives only, and the second one consisted of CDAs active toward both methylated and bulky aliphatic or aromatic substituents harboring nucleosides. In addition, the *N*, *S*, or *O* heteroatoms at the fourth position have only a moderate impact on the activity of CDAs. Tested CDAs does not deaminate isocytidine and N^3^-methyl-2′-deoxycytidine, and this is explained by the described reaction mechanism (*29*). The double and triple mutants of catalytic cysteine were completely inactive. This result confirms that the reaction of nucleophilic substitution is catalyzed exactly by the enzyme, and artifacts of the system can be excluded.

Analysis of the available 3D structures of CDAs revealed that the enzyme specificity depends mostly on the volume of the binding pocket. After comparing CDA_F14 and CDA_Bsu, CDA_Hsa, and CDA_Mmu structures, it can be seen that the β3α3 loop (amino acids 81 to 85) formed by amino acids from the 79th to the 88th position in CDA_F14 is more distant from the substrate binding site, and this factor may predetermine the binding of *N*(*O*,*S*)^4^-substituted nucleosides, especially with a bulky groups at this site. Moreover, the variable region of the β3α3 loop increases or decreases the binding pocket volume depending on the sequence: The hydrophobic amino acid residues, such as isoleucine or phenylalanine, restrict the space near the active center (CDA_Hsa and CDA_Mmu) contrarily to glycine or alanine (CDA_F14, CDA_Lsp, CDA_V47, CDA_Smo, and CDA_Hfi). Even more, the mobility of β3α3 loop influences an entering of substrate to the binding site. Determined kinetic parameters indicated that mutations in β3α3 loop region and the Phe^126^ residue mutants F126A and F126W substantially increase *K*_M_, and the deletion of the 83-to-85 site almost completely inactivates CDA_F14 enzyme. The specificity of the enzymes also depends, in part, on the composition of amino acids of this region.

Hence, this study thus opens up new avenues of research into enzymes that act on a wider set of nucleosides and their derivatives. These enzymes could be a part of repair and defense systems in living organisms. The data gathered on the diversity of CDA substrates should open new insights into the in vivo turnover of the modified nucleosides and into development of novel antiviral and antitumor prodrug–based therapies.

## MATERIALS AND METHODS

### Commercial nucleoside derivatives

Capecitabine, cytidine, cytosine, *N*^4^-acetylcytosine, and cytosine β-d-arabinofuranoside were obtained from Sigma-Aldrich (Germany). *N*^4^-acetyl-2′-deoxy-5′-*O*-DMT-cytidine, *N*^4^-benzoylcytidine, *N*^4^-benzoyl-5-methylcytidine, 2′-deoxy-5-hydroxycytidine, 2′-deoxy-5-hydroxymethylcytidine, 5-hydroxymethylcytidine, 2′-deoxy-5-propynylcytidine, pseudoisocytidine, isocytidine, 5-fluorocytidine, 2-thiocytidine, 2′-deoxy-5-methylcytidine, 2′,5′-dideoxycytidine, 2′,3′-dideoxycytidine, 2′-*O*-methylcytidine, 3′-azido-*N*^4^-benzoyl-2′,3′-dideoxycytidine, and *N*^3^-methyl-2′-deoxy cytidine were purchased from Carbosynth (UK); 3′-*O*-levulinoyl-*N*^4^-benzoyl-2′-deoxycytidine, 5′-*O*-levulinoyl-*N*^4^-benzoyl-2′-deoxycytidine, 3′-*O*-acetyl-*N*^4^-benzoyl-2′-deoxycytidine, *S*^4^-*n*-propylthiouridine-5′-triphosphate, *S*^4^-*iso*-propylthiouridine-5′-triphosphate, and *S*^4^-*iso*-butylthiouridine-5′-triphosphate were obtained from Jena Bioscience. *S*^4^-*n*-propylthiouridine-5′-triphosphate, *S*^4^-*iso*-propylthiouridine-5′-triphosphate, and *S*^4^-*iso*-butylthiouridine-5′-triphosphate were dephosphorylated using FastAP alkaline phosphatase (Thermo Fisher Scientific) before treatment with CDAs. *N*^4^-acetylcytidine, *N*^4^-acetyl-2′-deoxycytidine, *N*^4^-benzoyl-2′-deoxycytidine, and *N*^4^-isobutyryl-2′-deoxycytidine were purchased from Combi-Blocks (USA), and *N*^4^-benzoylcytosine was purchased from Bide Pharmatech Ltd.

### Comercial enzyme

Recombinant human CDA (CDA_Hsa) was obtained from Sigma-Aldrich.

### Synthetic nucleoside derivatives

*N*^4^-hexanoyl-2′-deoxycytidine, *N*^4^-nicotinoyl-2′-deoxycytidine, *N*^4^-(3-acetyl)-benzoyl-2′-deoxycytidine, *N*^4^-(4-acetyl)-benzoyl-2′-deoxycytidine, *N*^4^-(2-benzoyl)-benzoyl-2′-deoxycytidine, *N*^4^-(3-benzoyl)-benzoyl-2′-deoxycytidine, and *N*^4^-(4-benzoyl)-benzoyl-2′-deoxycytidine were synthesized as described previously (*40*). Remaining compounds used in this study were synthesized by adapted or modified procedures. Synthetic approach, schemes, and detailed synthetic procedures are provided in the Supplementary Materials.

### Functional screening of metagenomic libraries

Metagenomic libraries were constructed from soil and sediment samples using pUC19 plasmid vector as described earlier (*41*). The list of used libraries is described in the table S2. The selection of CDAs was performed in uridine auxotrophic *E. coli* DH10B Δ*pyrFEC*::Km (*23*) cells by using the published protocol (*41*). False-positive hits were eliminated by the restreaking on M9 medium without uridine or uridine derivative (fig. S1).

### DNA sequencing and gene annotation

Nucleotide sequences were determined at Macrogen Europe, Netherlands using the following sequencing primers: M13F-pUC (5′-GTTTTCCCAGTCACGAC-3′), M13R-pUC (5′-CAGGAAACAGCTATGAC-3′), T7 promoter (5´-TAATACGACTCACTATAGGG-3′), and LIC reverse sequencing primer, 24-nucleotide oligomer (5′-GAGCGGATAACAATTTCACACAGG-3′). ORFs were analyzed using Benchling (Biology Software), 2020, retrieved from https://benchling.com. Homology search was conducted using the Blast server (www.ncbi.nlm.nih.gov/BLAST). Phylogenetic analysis was conducted using the neighbor-joining tree routine of MEGA X software (version 10.0.5) (*42*). The sequence alignment was performed using ClustalW in MEGA X.

### Cloning, overexpression, and purification of CDAs

Metagenomic CDAs encoding genes, the genes of CDAs from *E. coli* DH10B (CDA_Eco), and *B. subtilis* laboratory strain 35 (CDA_Bsu) genes were amplified with Phusion DNA polymerase using primers listed in the table S6. Other CDA genes were synthesized at Twist Bioscience, USA, South San Francisco. The cloning, overexpression, and purification procedures were performed as described previously (*41*). CDA_F14 for crystallization was additionally purified by gel filtration on Superdex 200 (Cytiva).

### Construction of *E. coli* HMS174 Δ*pyrF*∆*cdd* strain

*E. coli* strain BW25113 JW1273 from Keio collection (*43*) served as the genetic source for the *pyrF::FRT-kan^R^-FRT* deletion cassette; JW2131 strain for *cdd::FRT-kan^R^-FRT* deletion cassette. The deletion cassettes were amplified using dpyrFF (5′-CTTCAGCGTCATCCGACCAT-3′) and dpyrFR (5′-CGCCTGCGAGTTTTACCTTC-3′), dCddF (5′-ACATTGCTTAATGCGATGCGT-3′), and dCddR (5′-GGGGAGATCCTGCAATTCGT-3′) DNA primers. Chromosomal in-frame gene deletions in *E. coli* and subsequent *kan^R^* marker removal were accomplished via a Quick & Easy *E. coli* gene deletion kit (Gene Bridges GmbH).

### Site-directed and random mutagenesis

Mutations in the CDAs genes were introduced by using the protocol “Phusion Site-Directed Mutagenesis Kit” (Thermo Fisher Scientific). Primer pairs used for specific mutagenesis listed in the table S7. The primers were phosphorylated before polymerase chain reaction (PCR). The mutant HQHH was prepared by using C53HR56Q mutant as template DNA for PCR. A two-step mutagenesis was used for random mutagenesis of selected regions. In the first step, a pair of primes surrounding the region that will be randomized, but not including it, was used to create a deletion of the region of interest (primers F14_del83-85). In the second round, one of the original primers was modified by adding a random nucleotide overhang that fills the deleted region to its 5′ end, and the other primer was left unmodified (F14_mutdel83-85). PCR fragments were ligated at 4°C for 18 hours, and after incubation, DNA plasmids of site-directed mutants were transformed into DH5α *E. coli* cells and plated on LB agar plates supplemented with ampicillin (100 μg/ml). The functional screening of active random mutants was selected by using *E. coli* HMS174 Δ*pyrF*∆*cdd* strain and *N*^4^-benzoyl-2′-deoxycytidine as a 2′-deoxyuridine source (*21*). Isopropyl-β-d-thiogalactopyranoside (1 mM) was added to a selective medium for induction of gene expression.

### Test of substrate scope of hydrolases

A hydrolytic activity of enzymes was analyzed by TLC, HPLC-MS, and GC-MS methods. Nucleosides (substrates) and hydrolysis reaction products were tested by TLC and HPLC-MS method. Other reaction products (amide, amine, thiol, and carbamate) were detected by GC-MS. TLC was conducted on the Merck silica gel 60 F_254_ plates using chloroform and methanol (5:1) mixture as eluent. HPLC-MS analyses were performed using a HPLC system (Shimadzu, Japan) and a mass spectrometer (LCMS-2020, Shimadzu, Japan) equipped with an electrospray ionization source. The chromatographic separation was conducted using a Hydrosphere C18 column. The data were analyzed using the LabSolutions LCMS software.

The substrate scope of the CDAs was assayed in reaction mixture containing 45 mM potassium phosphate buffer (pH 7.5), 1 μl of enzyme (0.1 to 4.6 μg per reaction), and 4 mM substrate. The total reaction mix volume was 20 μl. Reaction mixtures were incubated at 30°C temperature up to 3 hours and at RT overnight.

The specific activity of CDA_F14 was assayed in reaction mixture containing 45 mM potassium phosphate buffer (pH 7.5), 2.15 or 21.5 μg of enzyme, and 4 mM substrate in a total reaction volume equal to 50 μl. After incubation (2 min to 19 hours, 22° to 23°C), the reaction was stopped by adding 50 μl of acetonitrile. The exact time of hydrolysis, the amount of enzyme in reactions, and the calibration curves of quantification with different substrates are provided in datafile S1. The quantity of reaction product was analyzed by HPLC at the ultraviolet 254-nm wavelength. The activity units were calculated from the calibration curve of quantitative standards.

#### GC-MS analysis of hydrolysis reaction products

GC-MS analyses were performed with a Shimadzu GCMS-QP2010 Ultra Plus (Kyoto, Japan). Chromatographic separation was achieved on a Rxi-5ms column (30 m × 0.25 mm internal diameter, 0.25 μm film thickness, Restek, USA) using helium as a carrier gas at 40 cm/s in a constant linear velocity mode (temperature program, 50°C (1 min), 50°C → 250°C (25°C/min); total run time, 15 min). The temperature of injector, interface, and ion source were 250°C. Detection was operated by selected ion monitoring mode [Electron Ionization (EI) mode], data were collected and analyzed using the GC-MS solution, version 2.71 (Kyoto, Japan). For GC-MS analysis, the reaction mixture consisted of 45 mM potassium phosphate buffer (pH 7.5), 2.4 mM substrate (from 50 to 100 mM stock in dimethyl sulfoxide), and protein (0.5 to 1 mg/ml). The total reaction mix volume was 1000 μl. The hydrolysis reaction products were extracted with ethyl acetate (3 × 400 μl).

### Kinetic experiments

The deamination of *N*^4^-benzoyl-2′-deoxycytidine and 2′-deoxycytidine was monitored by the decrease of absorbance at 310 nm (∆ϵ = cytidine 11,000 M^−1^ cm^−1^) or 290 nm (∆ϵ = deoxycytidine 1600 M^−1^ cm^−1^), respectively, using cuvettes maintained at 22° to 23°C. The reaction was started by adding an appropriate amount of protein to potassium phosphate buffer (pH 7.5; 25°C) supplemented with 0.3, 0.15, 0.1, 0.075, 0.05, 0.0375, 0.025, and 0.0125 mM *N*^4^-benzoyl-2′-deoxycytidine or 1, 0.8, 0.5, 0.4, 0.25, 0.2, 0.1, and 0.05 mM 2′-deoxycytidine. In these experiments, the experimental error in the measurements of the enzymatic activity has been determined by performing repeated assays at least three times. Kinetic parameters were calculated using lmfit-py 1.0.2 software, fitting the experimental data to a simple Michaelis-Menten kinetics scheme.

### t-SNE plot generation

The tested CDAs sequences were aligned by Clustal Omega (*44*). The alignment was used as a query profile for search against UniRef100 (*26*) using HHblits (*45*) sequence search tool. Sequences were then clustered together with tested CDA variants using MMseqs2 (*46*) easy cluster and 50% minimal sequence identity. Clusters containing the tested sequences were pooled and reclustered at 70% minimal sequence identity to obtain 1708 cluster representatives. The distance matrix from the sequences was generated using Clustal Omega. 2D embedding was calculated from the distance matrix using scikit-learn t-SNE module (*27*) with default settings (early exaggeration, 12; learning rate, 200; and maximum number of iterations, 1000), except that the embedding generation perplexity was set to 7.

### Protein crystallization and structure determination

The crystals of CDA_F14 were obtained by sitting drop vapor diffusion method at 19°C by mixing 0.75 μl of CDA_F14 [16 mg/ml in 20 mM tris-HCl (pH 7.6; 25°C) and 100 mM NaCl buffer] with 0.75 μl of the crystallization solution containing 28% 2-methyl-2,4-pentanediol, 0.02 M magnesium acetate, and 0.1 M Na-MES (pH 4.6). The crystals were flash-cooled for data collection at 100 K without additional cryoprotection. The x-ray diffraction dataset was collected at the EMBL/DESY Petra III P13 beamline (Germany) at 100 K. XDS (*47*), SCALA, and TRUNCATE (*48*) were used for data processing. The data collection and refinement statistics are presented in table S5. The homology model of CDA_F14 prepared by SWISS-MODEL server (https://swissmodel.expasy.org/) (*49*), using *B. subtilis* CDA (PDB ID 1JTK chain A) as a template, was used for molecular replacement in Phaser (*50*). Manual rebuilding of the models was performed in COOT (*51*), and the structure was refined with phenix.refine (*52*). All molecular scale representations were prepared using Chimera 1.16 (*53*).

### Protein structure modeling

CDA_F14 homology modeling was carried out using either the Bioinformatics Toolkit available at the Max Planck Institute for Developmental Biology (Tübingen, Germany; https://toolkit.tuebingen.mpg.de) (*31*, *54*), Robetta available at https://robetta.bakerlab.org/ (*55*, *56*), or AlphaFold2 API notebook available at https://colab.research.google.com/github/sokrypton/ColabFold/blob/main/AlphaFold2.ipynb (*30*). Modeling with the Bioinformatics Toolkit homologous templates were found using HHpred (*30*). Good structures (probability of >95%, identity of >40%, and resolution of <2.5 Å) were selected for homology modeling using MODELLER. The default parameters were used for modeling with Robetta or AlphaFold2. The best model overall was selected by comparing the quality of models produced by different methods using VoroMQA (available at https://bioinformatics.lt/wtsam/voromqa) (*57*, *58*) and checking for model agreement with known structures.

### Molecular docking

Molecular docking was performed using Autodock Vina (*28*). Substrates were docked into CDA_F14 poses obtained from molecular dynamics simulations every 0.5 ns. Protein structures were prepared for docking using USCF Chimera DockPrep software. Substrate structures were prepared using Avogadro software, minimized using GAFF force field, and protonated to pH 7.5. Molecular docking was performed into each CDA active site separately. Binding boxes were centered on Zn^2+^ ions found in the active site, and their dimensions were determined by the size of the substrate. Parameters used for docking were as follows: exhaustiveness = 50, num_modes = 20, and energy_range = 15. Docked structures were selected according to distances between the substrate and the residues relevant for the enzyme-substrate interactions in CDAs. These selected structures were then used for molecular dynamics simulations. The plot for embedding was generated using the 2D graphics package Matplotlib (*59*).

### Molecular dynamics

Molecular dynamics were performed using AMBER16 software. Protein structures were prepared using TLEAP, and substrates were prepared using ANTECHAMBER. The protein structures were parameterized using the ff14sb force field and substrates were parametrized using the GAFF force field. The enzyme-substrate complex was solvated using TIP3P molecular water in a box with a distance of 35 Å from the enzyme to the box boundary. The system was neutralized by adding the needed number of Na^+^ or Cl^−^ ions. The simulation had five steps. First, the system was minimized with sander and then heated to 300 K over 1 ns, then the system pressure was equilibrated to 1 bar over 2 ns, and then the system was equilibrated for a further 2 ns. The production simulation was run for 100 ns. Simulations were performed in constant volume periodic boundary conditions with isotropic pressure scaling. For heating, equilibration, and production simulations, the nonbonded cutoff was set to 12 Å, the temperature was maintained using Langevin dynamics with a collision frequency of 2 ps^−1^, and the pressure was maintained using the Berendsen barostat. The trajectory was integrated every 2 fs with the SHAKE algorithm for bond length control. Analysis of trajectories was performed using CPPTRAJ.

### Assessing enzyme binding pocket SASA relationship with substrate selectivity

Enzyme binding pocket SASA relationship to substrate selectivity was checked for CDA enzymes modeled using AlphaFold2 and CDA enzymes modeled with modeler software using the determined CDA_F14 crystal structure as a template. The modeled monomers of CDA (CDA_EH, CDA_Lsp, CDA_Ppo, CDA_Pin, CDA_Pco, CDA_Smo, CDA_Tar, CDA_Dfa, CDA_Hfi, and CDA_Mtu) and crystal structures [CDA_F14 and CDA_Bsu (PDB 1JTK)] were superimposed onto each other. Mouse CDA monomer with bound cytidine (PDB 2FR6) was also superimposed onto the structures. Atoms that were within 5 Å of the cytidine in mouse CDA were considered to belong to the binding pocket. Per-atom SASA was calculated using the Shrake-Rupley algorithm (*60*) implemented in the Biopython package version 1.79 (https://biopython.org/) (*61*). Binding pocket SASA was determined by summing SASA of atoms that were considered to belong to the binding pocket. Substrate volume was calculated using RDKit version 2022.03.1 (www.rdkit.org/). The Pearson correlation between the binding pocket SASA and substrate volume was determined using NumPy version 1.22.3 (https://numpy.org/doc/stable/index.html).

## Supplementary Materials

### This PDF file includes:

Supplementary Text Figs. S1 to S13 Tables S1 to S7 References

## Notes

View/request a protocol for this paper from *Bio-protocol*.

## References

[1] P. Boccaletto, F. Stefaniak, A. Ray, A. Cappannini, S. Mukherjee, E. Purta, M. Kurkowska, N. Shirvanizadeh, E. Destefanis, P. Groza, G. Avşar, A. Romitelli, P. Pir, E. Dassi, S. G. Conticello, F. Aguilo, J. M. Bujnicki, MODOMICS: A database of RNA modification pathways. 2021 update. Nucleic Acids Res. 50, D231–D235 (2022). 34893873 10.1093/nar/gkab1083 PMC8728126

[2] A. J. Sood, C. Viner, M. M. Hoffman, DNAmod: The DNA modification database. J. Cheminform. 11, 30 (2019). 31016417 10.1186/s13321-019-0349-4 PMC6478773

[3] P. J. McCown, A. Ruszkowska, C. N. Kunkler, K. Breger, J. P. Hulewicz, M. C. Wang, N. A. Springer, J. A. Brown, Naturally occurring modified *ribonucleosides*. Wiley Interdiscip. Rev. RNA 11, e1595 (2020). 32301288 10.1002/wrna.1595 PMC7694415

[4] S. Hong, X. Cheng, DNA base flipping: A general mechanism for writing, reading, and erasing DNA modifications. Adv. Exp. Med. Biol. 945, 321–341 (2016). 27826845 10.1007/978-3-319-43624-1_14 PMC5542066

[5] W. L. Nyhan, Nucleotide Synthesis Via Salvage Pathway, in *eLS* (John Wiley & Sons Ltd, 2014).

[6] L. M. Iyer, D. Zhang, I. B. Rogozin, L. Aravind, Evolution of the deaminase fold and multiple origins of eukaryotic editing and mutagenic nucleic acid deaminases from bacterial toxin systems. Nucleic Acids Res. 39, 9473–9497 (2011). 21890906 10.1093/nar/gkr691 PMC3239186

[7] G. Niu, H. Tan, Nucleoside antibiotics: Biosynthesis, regulation, and biotechnology. Trends Microbiol. 23, 110–119 (2015). 25468791 10.1016/j.tim.2014.10.007

[8] L. Li, J. Wu, Z. Deng, T. M. Zabriskie, X. He, Streptomyces lividans blasticidin S deaminase and its application in engineering a blasticidin S-producing strain for ease of genetic manipulation. Appl. Environ. Microbiol. 79, 2349–2357 (2013). 23377931 10.1128/AEM.03254-12 PMC3623232

[9] N. Navaratnam, R. Sarwar, An overview of cytidine deaminases. Int. J. Hematol. 83, 195–200 (2006). 16720547 10.1532/IJH97.06032

[10] A. R. Ramiro, V. M. Barreto, Activation-induced cytidine deaminase and active cytidine demethylation. Trends Biochem. Sci. 40, 172–181 (2015). 25661247 10.1016/j.tibs.2015.01.006

[11] L. Betts, S. Xiang, S. A. Short, R. Wolfenden, C. W. Carter Jr., Cytidine deaminase. The 2.3 A crystal structure of an enzyme: Transition-state analog complex. J. Mol. Biol. 235, 635–656 (1994). 8289286 10.1006/jmbi.1994.1018

[12] S. E. Faivre-Nitschke, J. M. Grienenberger, J. M. Gualberto, A prokaryotic-type cytidine deaminase from *Arabidopsis thaliana* gene expression and functional characterization. Eur. J. Biochem. 263, 896–903 (1999). 10469156 10.1046/j.1432-1327.1999.00591.x

[13] W. Liu, F. Shang, Y. Chen, J. Lan, L. Wang, J. Chen, P. Gao, N.-C. Ha, C. Quan, K. H. Nam, Y. Xu, Biochemical and structural analysis of the *Klebsiella pneumoniae* cytidine deaminase CDA. Biochem. Biophys. Res. Commun. 519, 280–286 (2019). 31495495 10.1016/j.bbrc.2019.08.167

[14] S. Costanzi, S. Vincenzetti, G. Cristalli, A. Vita, Human cytidine deaminase: A three-dimensional homology model of a tetrameric metallo-enzyme inferred from the crystal structure of a distantly related dimeric homologue. J. Mol. Graph. Model. 25, 10–16 (2006). 16303324 10.1016/j.jmgm.2005.10.008

[15] E. Johansson, N. Mejlhede, J. Neuhard, S. Larsen, Crystal structure of the tetrameric cytidine deaminase from *Bacillus subtilis* at 2.0 Å resolution. Biochemistry 41, 2563–2570 (2002). 11851403 10.1021/bi011849a

[16] C. Serdjebi, G. Milano, J. Ciccolini, Role of cytidine deaminase in toxicity and efficacy of nucleosidic analogs. Expert Opin. Drug Metab. Toxicol. 11, 665–672 (2015). 25495470 10.1517/17425255.2015.985648

[17] C. M. Walko, C. Lindley, Capecitabine: A review. Clin. Ther. 27, 23–44 (2005). 15763604 10.1016/j.clinthera.2005.01.005

[18] R. M. Cohen, R. Wolfenden, Cytidine deaminase from *Escherichia coli*. J. Biol. Chem. 246, 7561–7565 (1971). 4944311

[19] A. J. Burke, W. R. Birmingham, Y. Zhuo, T. W. Thorpe, B. Zucoloto da Costa, R. Crawshaw, I. Rowles, J. D. Finnigan, C. Young, G. M. Holgate, M. P. Muldowney, S. J. Charnock, S. L. Lovelock, N. J. Turner, A. P. Green, An engineered cytidine deaminase for biocatalytic production of a key intermediate of the Covid-19 antiviral molnupiravir. J. Am. Chem. Soc. 144, 3761–3765 (2022). 35224970 10.1021/jacs.1c11048 PMC8915250

[20] A. M. Goble, H. Fan, A. Sali, F. M. Raushel, Discovery of a cytokinin deaminase. ACS Chem. Biol. 6, 1036–1040 (2011). 21823622 10.1021/cb200198c PMC3199332

[21] N. Urbelienė, R. Meškienė, M. Tiškus, R. Stanislauskienė, A. Aučynaitė, A. Laurynėnas, R. Meškys, A rapid method for the selection of amidohydrolases from metagenomic libraries by applying synthetic nucleosides and a uridine auxotrophic host. Catalysts 10, 445 (2020).

[22] A. Frances, P. Cordelier, The emerging role of cytidine deaminase in human diseases: A new opportunity for therapy? Mol. Ther. 28, 357–366 (2020). 31870623 10.1016/j.ymthe.2019.11.026 PMC7001087

[23] A. Aučynaitė, R. Rutkienė, R. Gasparavičiūtė, R. Meškys, J. Urbonavičius, A gene encoding a DUF523 domain protein is involved in the conversion of 2-thiouracil into uracil. Environ. Microbiol. Rep. 10, 49–56 (2018). 29194984 10.1111/1758-2229.12605

[24] K. Clark, I. Karsch-Mizrachi, D. J. Lipman, J. Ostell, E. W. Sayers, GenBank. Nucleic Acids Res. 44, D67–D72 (2016). 26590407 10.1093/nar/gkv1276 PMC4702903

[25] E. Johansson, J. Neuhard, M. Willemoës, S. Larsen, Structural, kinetic, and mutational studies of the zinc ion environment in tetrameric cytidine deaminase. Biochemistry 43, 6020–6029 (2004). 15147186 10.1021/bi035893x

[26] B. E. Suzek, H. Huang, P. McGarvey, R. Mazumder, C. H. Wu, UniRef: Comprehensive and non-redundant UniProt reference clusters. Bioinformatics 23, 1282–1288 (2007). 17379688 10.1093/bioinformatics/btm098

[27] F. Pedregosa, G. Varoquaux, A. Gramfort, V. Michel, B. Thirion, O. Grisel, M. Blondel, P. Prettenhofer, R. Weiss, V. Dubourg, J. Vanderplas, A. Passos, D. Cournapeau, Scikit-learn: Machine learning in Python. J. Mach. Learn. Res. 12, 2825–2830 (2011).

[28] O. Trott, A. J. Olson, AutoDock Vina: Improving the speed and accuracy of docking with a new scoring function, efficient optimization and multithreading. J. Comput. Chem. 31, 455–461 (2010). 19499576 10.1002/jcc.21334 PMC3041641

[29] T. Matsubara, M. Ishikura, M. Aida, A quantum chemical study of the catalysis for cytidine deaminase: Contribution of the extra water molecule. J. Chem. Inf. Model. 46, 1276–1285 (2006). 16711747 10.1021/ci050479k

[30] J. Jumper, R. Evans, A. Pritzel, T. Green, M. Figurnov, O. Ronneberger, K. Tunyasuvunakool, R. Bates, A. Žídek, A. Potapenko, A. Bridgland, C. Meyer, S. A. A. Kohl, A. J. Ballard, A. Cowie, B. Romera-Paredes, S. Nikolov, R. Jain, J. Adler, T. Back, S. Petersen, D. Reiman, E. Clancy, M. Zielinski, M. Steinegger, M. Pacholska, T. Berghammer, S. Bodenstein, D. Silver, O. Vinyals, A. W. Senior, K. Kavukcuoglu, P. Kohli, D. Hassabis, Highly accurate protein structure prediction with AlphaFold. Nature 596, 583–589 (2021). 34265844 10.1038/s41586-021-03819-2 PMC8371605

[31] L. Zimmermann, A. Stephens, S. Z. Nam, D. Rau, J. Kübler, M. Lozajic, F. Gabler, J. Söding, A. N. Lupas, V. Alva, A completely reimplemented MPI bioinformatics toolkit with a new HHpred server at its core. J. Mol. Biol. 430, 2237–2243 (2018). 29258817 10.1016/j.jmb.2017.12.007

[32] Z. A. Sánchez-Quitian, L. F. S. M. Timmers, R. A. Caceres, J. G. Rehm, C. E. Thompson, L. A. Basso, W. F. de Azevedo Jr., D. S. Santos, Crystal structure determination and dynamic studies of *Mycobacterium tuberculosis* cytidine deaminase in complex with products. Arch. Biochem. Biophys. 509, 108–115 (2011). 21295009 10.1016/j.abb.2011.01.022

[33] N. Shigi, Biosynthesis and degradation of sulfur modifications in tRNAs. Int. J. Mol. Sci. 22, 11937 (2021). 34769366 10.3390/ijms222111937 PMC8584467

[34] C. Borek, V. F. Reichle, S. Kellner, Synthesis and metabolic fate of 4-methylthiouridine in bacterial tRNA. Chembiochem 21, 2768–2771 (2020). 32394608 10.1002/cbic.202000272 PMC7586944

[35] R. Stanislauskienė, A. Laurynėnas, R. Rutkienė, A. Aučynaitė, D. Tauraitė, R. Meškienė, N. Urbelienė, A. Kaupinis, M. Valius, L. Kaliniene, R. Meškys, YqfB protein from *Escherichia coli*: An atypical amidohydrolase active towards N4-acylcytosine derivatives. Sci. Rep. 10, 788 (2020). 31964920 10.1038/s41598-020-57664-w PMC6972931

[36] N. Shrivastav, D. Li, J. M. Essigmann, Chemical biology of mutagenesis and DNA repair: Cellular responses to DNA alkylation. Carcinogenesis 31, 59–70 (2010). 19875697 10.1093/carcin/bgp262 PMC2802671

[37] A. E. Pegg, Multifaceted roles of alkyltransferase and related proteins in DNA repair, DNA damage, resistance to chemotherapy and research tools. Chem. Res. Toxicol. 24, 618–639 (2011). 21466232 10.1021/tx200031q PMC3095683

[38] M. Zimmermann, M. Zimmermann-Kogadeeva, R. Wegmann, A. L. Goodman, Mapping human microbiome drug metabolism by gut bacteria and their genes. Nature 570, 462–467 (2019). 31158845 10.1038/s41586-019-1291-3 PMC6597290

[39] B. Javdan, J. G. Lopez, P. Chankhamjon, Y. C. J. Lee, R. Hull, Q. Wu, X. Wang, S. Chatterjee, M. S. Donia, Personalized mapping of drug metabolism by the human gut microbiome. Cell 181, 1661–1679.e22 (2020). 32526207 10.1016/j.cell.2020.05.001 PMC8591631

[40] J. Jakubovska, D. Tauraite, L. Birštonas, R. Meškys, *N*^4^-acyl-2′-deoxycytidine-5′-triphosphates for the enzymatic synthesis of modified DNA. Nucleic Acids Res. 46, 5911–5923 (2018). 29846697 10.1093/nar/gky435 PMC6158702

[41] N. Urbelienė, S. Kutanovas, R. Meškienė, R. Gasparavičiūtė, D. Tauraitė, M. Koplūnaitė, R. Meškys, Application of the uridine auxotrophic host and synthetic nucleosides for a rapid selection of hydrolases from metagenomic libraries. J. Microbial. Biotechnol. 12, 148–160 (2019). 10.1111/1751-7915.13316 PMC6302743 30302933

[42] S. Kumar, G. Stecher, M. Li, C. Knyaz, K. Tamura, MEGA X: Molecular evolutionary genetics analysis across computing platforms. Mol. Biol. Evol. 35, 1547–1549 (2018). 29722887 10.1093/molbev/msy096 PMC5967553

[43] T. Baba, T. Ara, M. Hasegawa, Y. Takai, Y. Okumura, M. Baba, K. A. Datsenko, M. Tomita, B. L. Wanner, H. Mori, Construction of *Escherichia coli* K-12 in-frame, single-gene knockout mutants: The Keio collection. Mol. Syst. Biol. 2, 2006.0008 (2006). 10.1038/msb4100050 PMC1681482 16738554

[44] F. Sievers, D. G. Higgins, Clustal omega for making accurate alignments of many protein sequences. Protein Sci. Publ. Protein Soc. 27, 135–145 (2018). 10.1002/pro.3290 PMC5734385 28884485

[45] M. Remmert, A. Biegert, A. Hauser, J. Söding, HHblits: Lightning-fast iterative protein sequence searching by HMM-HMM alignment. Nat. Methods 9, 173–175 (2012). 10.1038/nmeth.1818 22198341

[46] M. Steinegger, J. Söding, MMseqs2 enables sensitive protein sequence searching for the analysis of massive data sets. Nat. Biotechnol. 35, 1026–1028 (2017). 29035372 10.1038/nbt.3988

[47] W. Kabsch, XDS. Acta Crystallogr. D Biol. Crystallogr. 66, 125–132 (2010). 20124692 10.1107/S0907444909047337 PMC2815665

[48] The CCP4 suite: Programs for protein crystallography. Acta Crystallogr. D Biol. Crystallogr. 50, 760–763 (1994). 15299374 10.1107/S0907444994003112

[49] A. Waterhouse, M. Bertoni, S. Bienert, G. Studer, G. Tauriello, R. Gumienny, F. T. Heer, T. A. P. de Beer, C. Rempfer, L. Bordoli, R. Lepore, T. Schwede, SWISS-MODEL: Homology modelling of protein structures and complexes. Nucleic Acids Res. 46, W296–W303 (2018). 29788355 10.1093/nar/gky427 PMC6030848

[50] A. J. McCoy, R. W. Grosse-Kunstleve, P. D. Adams, M. D. Winn, L. C. Storoni, R. J. Read, Phaser crystallographic software. J. Appl. Cryst. 40, 658–674 (2007). 19461840 10.1107/S0021889807021206 PMC2483472

[51] P. Emsley, K. Cowtan, Coot: Model-building tools for molecular graphics. Acta Crystallogr. D Biol. Crystallogr. 60, 2126–2132 (2004). 15572765 10.1107/S0907444904019158

[52] P. V. Afonine, R. W. Grosse-Kunstleve, N. Echols, J. J. Headd, N. W. Moriarty, M. Mustyakimov, T. C. Terwilliger, A. Urzhumtsev, P. H. Zwart, P. D. Adams, Towards automated crystallographic structure refinement with phenix.refine. Acta Crystallogr. D Biol. Crystallogr. 68, 352–367 (2012). 22505256 10.1107/S0907444912001308 PMC3322595

[53] E. F. Pettersen, T. D. Goddard, C. C. Huang, G. S. Couch, D. M. Greenblatt, E. C. Meng, T. E. Ferrin, UCSF Chimera-a visualization system for exploratory research and analysis. J. Comput. Chem. 25, 1605–1612 (2004). 15264254 10.1002/jcc.20084

[54] F. Gabler, S.-Z. Nam, S. Till, M. Mirdita, M. Steinegger, J. Söding, A. N. Lupas, V. Alva, Protein sequence analysis using the MPI bioinformatics toolkit. Curr. Protoc. Bioinforma. 72, e108 (2020). 10.1002/cpbi.108 33315308

[55] M. Baek, F. DiMaio, I. Anishchenko, J. Dauparas, S. Ovchinnikov, G. R. Lee, J. Wang, Q. Cong, L. N. Kinch, R. D. Schaeffer, C. Millán, H. Park, C. Adams, C. R. Glassman, A. DeGiovanni, J. H. Pereira, A. V. Rodrigues, A. A. van Dijk, A. C. Ebrecht, D. J. Opperman, T. Sagmeister, C. Buhlheller, T. Pavkov-Keller, M. K. Rathinaswamy, U. Dalwadi, C. K. Yip, J. E. Burke, K. C. Garcia, N. V. Grishin, P. D. Adams, R. J. Read, D. Baker, Accurate prediction of protein structures and interactions using a three-track neural network. Science 373, 871–876 (2021). 34282049 10.1126/science.abj8754 PMC7612213

[56] N. Hiranuma, H. Park, M. Baek, I. Anishchenko, J. Dauparas, D. Baker, Improved protein structure refinement guided by deep learning based accuracy estimation. Nat. Commun. 12, 1340 (2021). 33637700 10.1038/s41467-021-21511-x PMC7910447

[57] J. Dapkūnas, K. Olechnovič, Č. Venclovas, Modeling of protein complexes in CAPRI Round 37 using template-based approach combined with model selection. Proteins 86 (Suppl. 1), 292–301 (2018). 28905467 10.1002/prot.25378

[58] K. Olechnovič, Č. Venclovas, VoroMQA: Assessment of protein structure quality using interatomic contact areas. Proteins 85, 1131–1145 (2017). 28263393 10.1002/prot.25278

[59] J. D. Hunter, Matplotlib: A 2D graphics environment. Comput. Sci. Eng. 9, 90–95 (2007).

[60] A. Shrake, J. A. Rupley, Environment and exposure to solvent of protein atoms. Lysozyme and insulin. J. Mol. Biol. 79, 351–371 (1973). 4760134 10.1016/0022-2836(73)90011-9

[61] P. J. A. Cock, T. Antao, J. T. Chang, B. A. Chapman, C. J. Cox, A. Dalke, I. Friedberg, T. Hamelryck, F. Kauff, B. Wilczynski, M. J. L. de Hoon, Biopython: Freely available Python tools for computational molecular biology and bioinformatics. Bioinformatics 25, 1422–1423 (2009). 19304878 10.1093/bioinformatics/btp163 PMC2682512

[62] R. A. Laskowski, M. B. Swindells, LigPlot+: Multiple ligand-protein interaction diagrams for drug discovery. J. Chem. Inf. Model. 51, 2778–2786 (2011). 21919503 10.1021/ci200227u

[63] Y. Gong, L. Chen, W. Zhang, R. Salter, Transglycosylation in the modification and isotope labeling of pyrimidine nucleosides. Org. Lett. 22, 5577–5581 (2020). 32628494 10.1021/acs.orglett.0c01941

[64] J. Milecki, J. Nowak, B. Skalski, S. Franzen, 5-Fluoro-4-thiouridine phosphoramidite: New synthon for introducing photoaffinity label into oligodeoxynucleotides. Bioorg. Med. Chem. 19, 6098–6106 (2011). 21917468 10.1016/j.bmc.2011.08.035

[65] Z. Kaleta, B. T. Makowski, T. Soós, R. Dembinski, Thionation using fluorous Lawesson’s reagent. Org. Lett. 8, 1625–1628 (2006). 16597126 10.1021/ol060208a

[66] G. Wenska, K. Taras-Goslinska, P. Filipiak, G. L. Hug, B. Marciniak, Photochemical reactions of 4-thiouridine disulfide and 4-benzylthiouridine—The involvement of the 4-pyrimidinylthiyl radical. Photochem. Photobiol. Sci. 7, 250–256 (2008). 18264594 10.1039/b713218b

[67] A. Kraszewski, A. M. Delort, R. Teoule, Synthesis of 4-mono- and dialkyl-2′-deoxycytidines and their insertion into an oligonucleotide. Tetrahedron Lett. 27, 861–864 (1986).

[68] X. Robert, P. Gouet, Deciphering key features in protein structures with the new ENDscript server. Nucleic Acids Res. 42, W320–W324 (2014). 24753421 10.1093/nar/gku316 PMC4086106

