# Supplementary material for: Cytidine deaminases catalyze the conversion of N(S,O)4-substituted pyrimidine nucleosides
Source: Sci Adv. 2023 Feb 3;9(5):eade4361. doi: 10.1126/sciadv.ade4361 (PMC9897663; doi:10.1126/sciadv.ade4361)
Supplement: Supplementary file 1 — Supplementary Text Figs. S1 to S13 Tables S1 to S7 References [file sciadv.ade4361_sm.pdf]

Science Advances
AAAS

Supplementary Materials for

Cytidine deaminases catalyze the conversion of *N*(*S*,*O*)^4-substituted pyrimidine nucleosides

Nina Urbelienė *et al.*

Corresponding author: Nina Urbelienė, nina.urbeliene@bchi.vu.lt; Rolandas Meškys, rolandas.meskys@bchi.vu.lt

Sci. Adv. **9**, eade4361 (2023)
DOI: 10.1126/sciadv.ade4361

The PDF file includes:

Supplementary Text
Figs. S1 to S13
Tables S1 to S7
Legends for data S1 to S3
References

Other Supplementary Material for this manuscript includes the following:

Data S1 to S3

Synthesis of nucleosides

Materials and equipment. All the chemicals for the synthesis were purchased from the common sources: Sigma Aldrich, Acros Organics, Alfa Aesar, Strem Chemicals, PENTA Chemicals, Fluorochem, Cambridge Isotope Laboratories, Inc. Unless otherwise noted, all the reagents were used without further purification. Solvents used in the reactions were distilled and dried prior the use. Reactions were monitored with thin-layer chromatography (TLC) using Merck TLC silica gel 60 F_{254} plates and UV lamp (254 nm) or Hanessian's stain for visualisation. Column chromatography was done on silica gel 60 (0.063–0.200 nm) (Merck). Reverse phase chromatography was carried out on Grace flash cartridges C-18. NMR spectra were recorded on a Bruker Avance III spectrometer (400 MHz for ^{1}H NMR and 100 MHz for ^{13}C NMR). All chemical shifts δ are reported in ppm with a reference to a residual solvent. HPLC-MS analyses were performed using a high performance liquid chromatography system, equipped with a photo diode array detector (SPD-M20A) and a mass spectrometer (LCMS-2020, Shimadzu, Japan) equipped with an ESI source. The chromatographic separation was conducted using a YMC Pack Pro column, 3×150 mm (YMC, Japan) at 40 °C and a mobile phase that consisted of 0.1 % formic acid water solution (solvent A), and acetonitrile (solvent B). Mass spectrometry data was acquired in both positive and negative ionization mode and analyzed using the LabSolutions LCMS software.

Acetylated precursors **49**, **70**, **71** and **72** were deacetylated to compounds **46**, **47**, **48**, **50** as follows: a reaction mixture containing 100 mM potassium phosphate buffer, pH 7.5, 0.5 mg/ml esterase SVG1(43) and 12-20 mg/ml of corresponding derivative (from 100 mM Stock in DMSO were incubated at 37°C up to 3 h.

Synthesis of N^4-alkylcytidine, S^4-alkylthiouridine and O^4-alkyluridine derivatives

General scheme for the synthesis of N^4-alkylcytidine, S^4-alkylthiouridine and O^4-alkyluridine derivatives is presented in the Figure S13.

Acetylation of uridine (64ad) and 5-fluorouridine (64bd). General procedure:

Acetic anhydride (6 eq.) was added dropwise to a 0.5 M solution of a substrate (1 eq.) in pyridine at room temperature. After 16 h, the reaction was quenched by the addition of methanol and the mixture was concentrated under reduced pressure by co-evaporating it with toluene at 40-45 °C (4 to 5 times). Traces of pyridine were removed by extraction: the crude mixture was dissolved in dichloromethane (1 g/25 mL) and it was washed twice with 1M HCl and once with water, then brine. The organic phase was dried over Na_2SO_4, filtered, and concentrated under reduced pressure.

2’, 3’, 5’-Tri-*O*-acetyluridine (66ad)

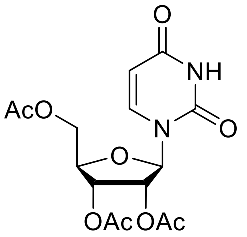

The acetylation (63) of uridine (**64ad**) was done on 41 mmol scale (10 g). The product was isolated as colourless glassy oil (15.1 g, 99 %) and was used in further reactions without additional purification. R_f = 0.6 [DCM:MeOH (14:1); ^{1}H NMR (400 MHz, $CDCl_3$) δ 9.44 (s, 1H), 7.39 (d, *J* = 8.2 Hz, 1H), 6.04 (d, *J* = 4.6 Hz, 1H), 5.79 (dd, *J* = 8.2, 2.1 Hz, 1H), 5.37 – 5.27 (m, 2H), 4.40 – 4.27 (m, 3H), 2.13 (s, 3H), 2.12 (s, 3H), 2.09 (s, 3H); ^{13}C NMR (100 MHz, $CDCl_3$) δ 170.3, 169.8 (2C), 163.0, 150.4, 139.4, 103.6, 87.6, 80.1, 72.8, 70.3, 63.3, 20.9, 20.6, 20.5.

2’,3’,5’-Tri-*O*-acetyl-5-fluorouridine (66bd)

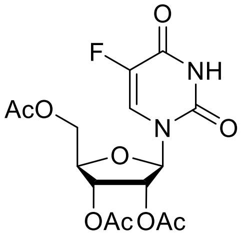

The acetylation (63) of 5-fluorouridine (**64bd**) was done on 5.8 mmol (1.51 g) scale. The product **66bd** was isolated as colourless glassy oil (2.2 g, 98 %) and was used in further reactions without additional purification. R_f = 0.7 [DCM:MeOH (10:1)]; ^{1}H NMR (400 MHz, $CDCl_3$) δ 9.46 (d, *J* = 4.8 Hz, 1H), 7.61 (d, *J* = 6.0 Hz, 1H), 6.11 – 6.06 (m, 1H), 5.36 – 5.27 (m, 2H), 4.43 – 4.28 (m, 3H), 2.10 (d, *J* = 0.8 Hz, 3H), 2.17 (s, 3H), 2.13 (s, 3H); ^{13}C NMR (100 MHz, $CDCl_3$) δ 170.2, 169.8 (2C), 156.6 (d, *J* = 27.0 Hz), 149.1, 141.0 (d, *J* = 239.4 Hz), 123.3 (d, *J* = 34.6 Hz), 87.2, 80.3, 72.9, 70.2, 63.1, 20.9, 20.6, 20.5.

2’, 3’, 5’-Tri-*O*-acetyl-5-fluoro-4-(1,2,4-triazol-1-yl)uridine (69)

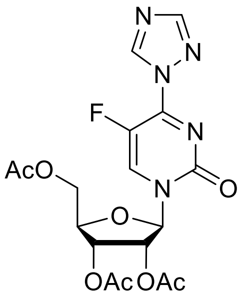

Compound **69** was synthesized using literature procedure (64). The compound **3b** was unstable during column chromatography purification. Therefore, it was prepared and, after isolating it from the liquid-liquid extraction, immediately used as a crude in further reactions (**49**, **70**, **71**, **72**). R_f = 0.4-0.5 [DCM:MeOH (30:1)]; ^{1}H NMR (400 MHz, $CDCl_3$) δ 9.25 (s, 1H), 8.41 (d, *J* = 6.6 Hz, 1H), 8.23 (s, 1H), 6.13 (d, *J* = 3.6 Hz, 1H), 5.46 (dd, *J* = 5.2, 3.5 Hz, 1H), 5.30 (d, *J* = 6.2 Hz, 1H), 4.51 (dt, *J* = 6.2, 2.8 Hz, 1H), 4.48 – 4.40 (m, 2H), 2.19 (s, 3H), 2.15 (s, 4H), 2.10 (s, 3H); ^{19}F NMR (375 MHz, $CDCl_3$) δ -156.77 (d, *J* = 6.6 Hz).

General procedure for *O*^{4}-alkyl-5-fluorouridines (49, 70, 71) and 5-fluoro-*S*^{4}-phenylthiouridine (72):

DIPEA (Hünigs base, 5 eq, 0.5 mmol, 87 µL) and thiophenol (5 eq, 0.5 mmol, 51 µL) or R_3OH (5 eq, 0.5 mmol) were added to a 0.1 M solution of 4-(1,2,4-triazol-1-yl)uridine (**69**) (1 eq, 0.1 mmol) in acetonitrile at room temperature. After 16 h, the reaction mixture was diluted with water (5 mL) and extracted with dichloromethane (2×10 mL). Combined organic phases were washed with brine (1×5 mL), then dried over Na_2SO_4, filtered, and concentrated under reduced pressure. Products were purified by column chromatography (DCM: MeOH 200:1 to 30:1).

2’, 3’, 5’-Tri-*O*-acetyl-5-fluoro-4-butoxyuridine (49)

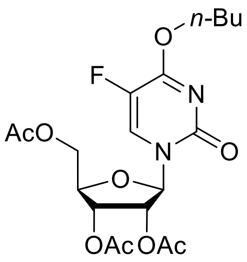

The product **49** was isolated as a colourless oil (53 mg, 32 % yield over 2 steps from 0.37 mmol of 2’,3’,5’-tri-*O*-acetyl-5-fluorouridine (**66bd**)). R_f = 0.25 [DCM:MeOH (30:1)]; ^{1}H NMR (400 MHz, $CDCl_3$) δ 7.79 (d, *J* = 5.8 Hz, 1H), 6.14 (dd, *J* = 4.4, 1.5 Hz, 1H), 5.34 (t, *J* = 4.9 Hz, 1H), 5.28 (t, *J* = 5.3 Hz, 1H), 4.52 – 4.40 (m, 2H), 4.43 – 4.21 (m, 3H), 2.16 (s, 3H), 2.10 (s, 3H), 2.09 (s, 3H), 1.77 (p, *J* = 6.9 Hz, 2H), 1.44 (h, *J* = 7.4 Hz, 2H), 0.95 (t, *J* = 7.4 Hz, 3H); ^{13}C NMR (100 MHz, $CDCl_3$) δ 170.1, 169.7, 169.6, 163.0 (d, *J* = 13.0 Hz), 153.5, 137.2 (d, *J* = 248.9 Hz), 126.3 (d, *J* = 32.2 Hz), 88.3, 79.9, 73.7, 69.7, 68.6, 62.7, 30.5, 20.8, 20.6 (2C), 19.1, 13.8; LCMS; HRMS (ESI) calcd for $C_{19}H_{25}O_9N_2FNa$ $(M+Na)^+$: 467.1436; found 467.1431.

2’, 3’, 5’-Tri-*O*-acetyl-5-fluoro-4-methoxyuridine (70)

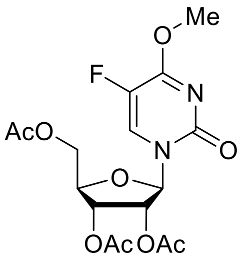

The product **70** was isolated as a colourless glassy oil (640 mg, 69 % yield over 2 steps from 2.3 mmol of 2’,3’,5’-tri-*O*-acetyl-5-fluorouridine (**66bd**)). R_f = 0.4 [DCM:MeOH (30:1)]; ^{1}H NMR (400 MHz, $CDCl_3$) δ 7.81 (d, *J* = 5.7 Hz, 1H), 6.15 (dd, *J* = 4.4, 1.5 Hz, 1H), 5.36 – 5.32 (m, 1H), 5.32 – 5.25 (m, 1H), 4.44 – 4.31 (m, 3H), 4.07 (s, 3H), 2.16 (s, 3H), 2.11 (s, 3H), 2.09 (s, 3H); ^{13}C NMR (100 MHz, $CDCl_3$) δ 170.1, 169.7, 169.6, 163.1 (d, *J* = 13.0 Hz), 153.4, 137.2 (d, *J* = 248.9 Hz), 126.4 (d, *J* = 32.0 Hz), 88.3, 80.0, 73.7, 69.7, 62.7, 55.5, 20.9, 20.6 (2C); ^{19}F NMR (375 MHz, $CDCl_3$) δ -167.60 (d, *J* = 6.0 Hz). LCMS; HRMS (ESI) calcd for $C_{16}H_{19}O_9N_2FNa$ $(M+Na)^+$: 425.0967; found 425.0964.

2’, 3’, 5’-Tri-*O*-acetyl-5-fluoro-4-benzyloxyuridine (71)

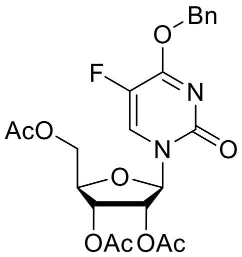

The product **71** was isolated as a colourless oil (40 mg, 23 % yield over 2 steps from 0.37 mmol of 2’,3’,5’-tri-*O*-acetyl-5-fluorouridine (**66bd**)). R_f = 0.25-0.35 [DCM:MeOH (30:1)]; ^{1}H NMR (400 MHz, $CDCl_3$) δ 7.84 (d, *J* = 5.7 Hz, 1H), 7.50 – 7.43 (m, 2H), 7.43 – 7.33 (m, 3H), 6.15 (dd, *J* = 4.4, 1.5 Hz, 1H), 5.50 (s, 2H), 5.36 (dd, *J* = 5.4, 4.3 Hz, 1H), 5.29 (t, *J* = 5.4 Hz, 1H), 4.44 – 4.31 (m, 3H), 2.16 (s, 3H), 2.12 (s, 3H), 2.09 (s, 3H); ^{13}C NMR (100 MHz, $CDCl_3$) δ 170.1, 169.7, 169.6, 162.6 (d, *J* = 13.1 Hz), 153.3, 137.1 (d, *J* = 249.2 Hz), 134.7, 128.9, 128.8 (4C), 126.7 (d, *J* = 32.0 Hz), 88.4, 79.9, 73.8, 70.0, 69.7, 62.7, 20.8, 20.61 (2C); HRMS (ESI) calcd for $C_{22}H_{23}O_9N_2FNa$ $(M+Na)^+$: 501.1280; found 501.1278.

2’, 3’, 5’-Tri-*O*-acetyl-5-fluoro-*S*⁴-phenylthiouridine (72)

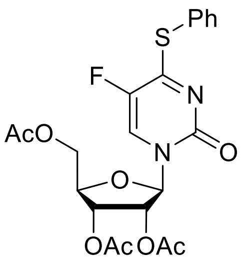

The product **72** was isolated as a colourless glassy oil (110 mg, 61 % yield over 2 steps from 0.376 mmol of 2’,3’,5’-tri-*O*-acetyl-5-fluorouridine (**66bd**)). R_f = 0.4 [DCM:MeOH (30:1)]; ^{1}H NMR (400 MHz, DMSO-d_6) δ 8.32 (d, *J* = 5.6 Hz, 1H), 7.62 – 7.48 (m, 5H), 5.82 (d, *J* = 3.7 Hz, 1H), 5.46 (dd, *J* = 6.2, 3.8 Hz, 1H), 5.32 (t, *J* = 6.1 Hz, 1H), 4.38 – 4.22 (m, 3H), 2.06 (s, 3H), 2.04 (s, 3H), 2.04 (s, 3H); ^{13}C NMR (100 MHz, DMSO-d_6) δ 170.0, 169.2, 169.1, 168.2 (d, *J* = 17.1 Hz), 150.7, 142.2 (d, *J* = 239.1 Hz), 135.7 (2C), 130.4, 129.6 (2C), 128.3 (d, *J* = 34.2 Hz), 124.1, 90.1, 79.4, 72.6, 69.2, 62.7, 20.5, 20.2 (2C); ^{19}F NMR (375 MHz, DMSO-d_6) δ -157.82, -157.84; HRMS (ESI) calcd for $C_{21}H_{22}O_8N_2SF$ $(M+H)^+$: 481.1075; found 481.1073.

Synthesis of thiones 68ad and 68bd can be accomplished directly from the uridines **64ad** and **64bd**, as described below, excluding the extraction procedure for the acetylation step. Otherwise, it can be followed from the (*) mark.

2',3',5'-Tri-*O*-acetyl-4-thiouridine (68ad)

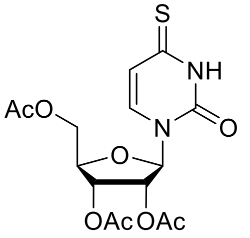

Acetic anhydride (172 mmol, 16.3 mL) was added in a dropwise manner to a solution of uridine (**64ad**, 41 mmol, 10 g) in pyridine (120 mL) and left to stir overnight at room temperature (65). The reaction was quenched with methanol (20 mL) and evaporated under reduced pressure. Most of the pyridine was removed by co-evaporation with toluene (5×30 mL). 2', 3', 5'-Tri-*O*-acetyluridine (**66ad**) was collected as a colourless oil (16.9 g) and (*) without further purification it was dissolved in dioxane (250 mL). It was followed by addition of phosphorus pentasulfide (91.2 mmol, 20.3 g) and subsequent reflux. After 2 h, the reaction mixture was cooled down and filtered. The remaining solids were washed with DCM (3×50 mL) and the filtrate was concentrated under reduced pressure. The remaining crude mixture was redissolved in DCM (200 mL), filtered through a pad of Celite (washing it with additional portion of DCM, 100 mL) and once again concentrated under reduced pressure. The resulting oil was subjected to column chromatography (two columns: 1. DCM: MeOH 100:1 to 25:1 and 2. THF:hex 1:2.3 to 1:1.8). 2',3',5'-Tri-*O*-acetyl-4-thiouridine (**68ad**) was isolated as a colorless glassy oil (10 g, 63 % yield). R_f = 0.65 [DCM:MeOH (19:1)]; ^{1}H NMR (400 MHz, $CDCl_3$) δ 10.12 (s, 1H), 7.23 (d, *J* = 7.7 Hz, 1H), 6.42 (dd, *J* = 7.7, 1.7 Hz, 1H), 5.97 (d, *J* = 4.9 Hz, 1H), 5.39 – 5.28 (m, 2H), 4.41 – 4.29 (m, 3H), 2.13 (s, 3H), 2.11 (s, 3H), 2.10 (s, 3H); ^{13}C NMR (100 MHz, $CDCl_3$) δ 189.9, 170.2, 169.7 (2C), 147.6, 133.7, 114.1, 88.1, 80.2, 73.0, 70.2, 63.1, 20.9, 20.6, 20.5.

2',3',5'-tri-*O*-acetyl-5-fluoro-4-thiouridine (68bd)

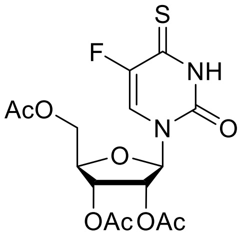

2',3',5'-tri-*O*-acetyl-5-fluoro-4-thiouridine (**68bd**) was synthesized following the procedure used for 2',3',5'-tri-*O*-acetyl-4-thiouridine (**68ad**). Reaction was done using 5-fluorouridine (**64bd**, 6.37 mmol, 1.67 g) (66). The desired product **68bd** was isolated as a yellow oil (2.5 g, 97%) which was unstable in a solution as well as when kept neat. R_f = 0.5 [DCM:MeOH (19:1)]; ^{1}H NMR (400 MHz, $CDCl_3$) δ 10.48 (s, 1H), 7.55 (d, *J* = 4.3 Hz, 1H), 6.02 (dd, *J* = 4.7, 1.4 Hz, 1H), 5.36 (t, *J* = 5.2 Hz, 1H), 5.34 – 5.24 (m, 1H), 4.43 – 4.30 (m, 2H), 2.16 (s, 3H), 2.11 (s, 6H); ^{13}C

NMR (100 MHz, $CDCl_3$) δ 180.3 (d, *J* = 31.2 Hz), 170.2, 169.8, 169.7, 147.5 (d, *J* = 227.3 Hz), 146.8, 119.2 (d, *J* = 41.3 Hz), 87.9, 80.3, 73.1, 69.9, 62.9, 20.8, 20.6, 20.5; ^{19}F NMR (375 MHz, $CDCl_3$) δ -144.57 (d, *J* = 4.1 Hz).

S^4-Methylthiouridine (37)

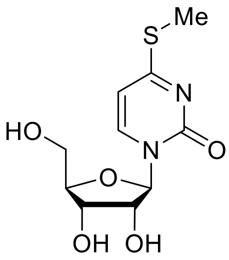

Iodomethane (5.5 mmol, 324 μL) was added to a solution of 2',3',5'-tri-*O*-acetyl-4-thiouridine (**68ad**, 3.1 mmol, 1.18 g) in water-methanol (1:5, 30 mL). It was followed by slow addition of 1M NaOH (3 mL, aq.) over 20 min. After 20 min of stirring at room temperature, reaction was quenched by adding acetic acid (3 mmol, 172 μL). Reaction mixture was concentrated under reduced pressure and subjected to column chromatography (DCM:MeOH 14:1 to 11:1). S^4-Methylthiouridine (**37**) was isolated as a white amorphous solid (370 mg, 43 %). R_f = 0.3 [DCM:MeOH (9:1)]; ^{1}H NMR (400 MHz, MeOD-d_4) δ 8.31 (d, *J* = 7.1 Hz, 1H), 6.47 (d, *J* = 7.2 Hz, 1H), 5.85 (d, *J* = 2.0 Hz, 1H), 4.21 – 4.11 (m, 2H), 4.12 – 4.03 (m, 1H), 3.94 (dd, *J* = 12.4, 2.4 Hz, 1H), 3.78 (dd, *J* = 12.4, 2.9 Hz, 1H), 2.53 (s, 3H); ^{13}C NMR (100 MHz, MeOD-d_4) δ 180.5, 156.4, 142.0, 104.9, 92.9, 85.9, 76.5, 70.1, 61.4, 12.9; HRMS (ESI) calcd for $C_{10}H_{15}O_5N_2S$ $(M+H)^+$: 275.0696; found 275.0691.

S^4-Ethylthiouridine (38)

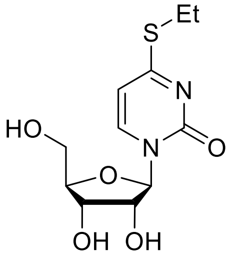

S^4-Ethylthiouridine (**38**) was synthesized following the procedure for S^4-methylthiouridine (**37**). The reaction was done using 2',3',5'-tri-*O*-acetyl-4-thiouridine (**68ad**, 1.29 mmol, 0.5 g) and iodoethane (4.2 mmol, 335 μL). It was quenched after 18 h. The product **38** was isolated as a white amorphous solid (310 mg, 83 %). R_f = 0.4 [DCM:MeOH (9:1)]; ^{1}H NMR (400 MHz, MeOD-d_4) δ 8.31 (d, *J* = 7.1 Hz, 1H), 6.43 (d, *J* = 7.2 Hz, 1H), 5.85 (d, *J* = 1.9 Hz, 1H), 4.18 – 4.11 (m, 2H), 4.12 – 4.03 (m, 1H), 3.94 (dd, *J* = 12.4, 2.5 Hz, 1H), 3.78 (dd, *J* = 12.4, 2.9 Hz, 1H), 3.17 (q, *J* = 7.3 Hz, 2H), 1.34 (t, *J* = 7.4 Hz, 3H); ^{13}C NMR (100 MHz, MeOD-d_4) δ 180.0, 156.4, 142.1, 105.2, 93.0, 85.8, 76.5, 70.1, 61.4, 25.0, 14.6.

S^4-Benzylthiouridine (42)

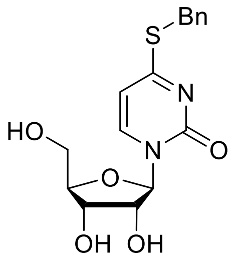

S^4-Benzylthiouridine (**42**) was synthesized following the procedure for S^4-methylthiouridine (**37**). The reaction was done using 2',3',5'-tri-*O*-acetyl-4-thiouridine (**68ad**, 3.1 mmol, 1.18 g) and benzyl bromide (3.4 mmol, 410 µL). It was quenched after 20 min. The product **42** was isolated as a white amorphous solid (700 mg, 64 %). $R_f = 0.4$ [DCM:MeOH (9:1)]; ^{1}H NMR (400 MHz, MeOD-*d₄*) δ 8.34 (d, *J* = 7.1 Hz, 1H), 7.44 – 7.38 (m, 2H), 7.34 – 7.19 (m, 3H), 6.43 (d, *J* = 7.2 Hz, 1H), 5.87 (d, *J* = 2.1 Hz, 1H), 4.44 (s, 2H), 4.18 – 4.11 (m, 2H), 4.08 (dt, *J* = 5.9, 2.7 Hz, 1H), 3.94 (dd, *J* = 12.4, 2.4 Hz, 1H), 3.78 (dd, *J* = 12.5, 2.8 Hz, 1H); 13C NMR (100 MHz, MeOD-d_4) δ 179.2, 156.3, 142.5, 138.3, 130.2 (2C), 129.6 (2C), 128.5, 104.8, 93.0, 85.9, 76.5, 70.1, 61.4, 34.6; HRMS (ESI) calcd for $C_{16}H_{19}O_5N_2S$ $(M+H)^+$: 351.1009; found 351.1007.

5-Fluoro-S^4-methylthiouridine (43)

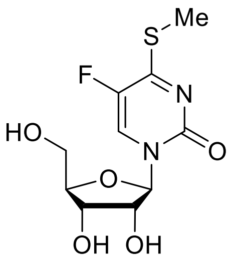

5-Fluoro-S^4-methylthiouridine (**43**) was synthesized following the procedure for S^4-methylthiouridine (**37**). The reaction was done using 2',3',5'-tri-*O*-acetyl-5-fluoro-4-thiouridine (**68bd**, 1.24 mmol, 0.5 g) and quenched after 1 h. The product **37** was isolated as a white amorphous solid (90 mg, 25 %). $R_f = 0.4$ [DCM:MeOH (9:1)]; ^{1}H NMR (400 MHz, MeOD-d_4) δ 8.61 (d, *J* = 5.8 Hz, 1H), 5.80 (t, *J* = 1.8 Hz, 1H), 4.21 – 4.11 (m, 2H), 4.08 (dt, *J* = 7.1, 2.4 Hz, 1H), 3.98 (dd, *J* = 12.4, 2.4 Hz, 1H), 3.80 (dd, *J* = 12.4, 2.3 Hz, 1H), 2.56 (s, 3H); ^{13}C NMR (100 MHz, MeOD-d_4) δ 171.3 (d, *J* = 17.6 Hz), 154.6, 145.7 (d, *J* = 240.0 Hz), 127.1 (d, *J* = 35.4 Hz), 92.9, 85.8, 76.6, 69.6, 60.8, 12.0 (d, *J* = 2.2 Hz); HRMS (ESI) calcd for $C_{10}H_{14}O_5N_2SF$ $(M+H)^+$: 293.0602; found 293.0603.

5-Fluoro-S^4-ethylthiouridine (44)

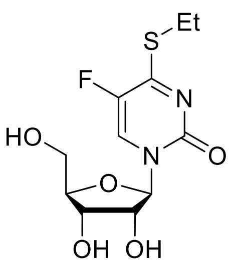

5-Fluoro-S^4-ethylthiouridine (**44**) was synthesized following the procedure for S^4-methylthiouridine (**37**). The reaction was done using 2',3',5'-tri-*O*-acetyl-5-fluoro-4-thiouridine (**68bd**, 1.24 mmol, 0.5 g) and iodoethane (2.5 mmol, 200 µL). It was quenched after 2 h. The product **44** was isolated as a white amorphous solid (150 mg, 39 %). $R_f = 0.5$ [DCM:MeOH (9:1)]; ^{1}H NMR (400 MHz, MeOD-d_4) δ 8.61 (d, *J* = 5.8 Hz, 1H), 5.80 (t, *J* = 1.7 Hz, 1H), 4.21 – 4.11 (m, 2H), 4.08 (dt, *J* = 7.0, 2.3 Hz, 1H), 3.98 (dd, *J* = 12.4, 2.4 Hz, 1H), 3.79 (dd, *J* = 12.4, 2.3 Hz, 1H), 3.22 (q, *J* = 7.4 Hz, 2H), 1.37 (t, *J* = 7.4 Hz, 3H); ^{13}C NMR (100 MHz, MeOD-d_4) δ

170.9 (d, J = 17.4 Hz), 154.6, 145.5 (d, J = 239.9 Hz), 127.3 (d, J = 35.4 Hz), 92.9, 85.8, 76.6, 69.7, 60.8, 24.3 (d, J = 1.9 Hz), 14.4; HRMS (ESI) calcd for $C_{11}H_{16}O_5N_2SF$: 307.0758; found 307.0760.

5-Fluoro-*S*⁴-benzylthiouridine (45)

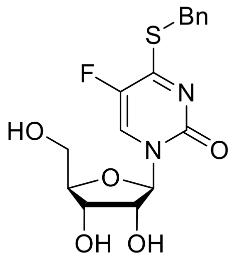

5-Fluoro-S^4-benzylthiouridine (**45**) was synthesized following the procedure for S^4-methylthiouridine (**37**). The reaction was done using 2',3',5'-tri-*O*-acetyl-5-fluoro-4-thiouridine (**68bd**, 1.24 mmol, 0.5 g) and benzyl bromide (2.0 mmol, 240 μL). It was quenched after 2 h. The product **45** was isolated as a white amorphous solid (130 mg, 28 %). R_f = 0.5 [DCM:MeOH (9:1)]; ^{1}H NMR (400 MHz, MeOD-d_4) δ 8.64 (d, J = 5.7 Hz, 1H), 7.47 – 7.39 (m, 2H), 7.35 – 7.20 (m, 3H), 5.82 (t, J = 1.6 Hz, 1H), 4.48 (s, 2H), 4.21 – 4.12 (m, 2H), 4.08 (dt, J = 6.7, 2.4 Hz, 1H), 3.98 (dd, J = 12.4, 2.4 Hz, 1H), 3.79 (dd, J = 12.5, 2.3 Hz, 1H); ^{13}C NMR (100 MHz, MeOD-d_4) δ 170.1 (d, J = 17.9 Hz), 154.4, 145.2 (d, J = 239.7 Hz), 137.8, 130.3 (2C), 129.7 (2C), 128.7, 127.7 (d, J = 35.5 Hz), 93.0, 85.8, 76.6, 69.6, 60.8, 33.8 (d, J = 1.8 Hz; HRMS (ESI) calcd for $C_{16}H_{18}O_5N_2SF$ $(M+H)^+$: 369.0919; found 369.0919.

Synthesis of N⁴-alkylated 2'-deoxycytidine derivatives. General procedure.

The alkylation of 4-thio-2’-deoxy-uridines was accomplished using modified literature procedure (67). This synthesis can be done either on acetylated 4-thio-2’-deoxyuridines or as described here. Desired amine (0.45 mmol) was added to a solution of -4-thio-2'-deoxyuridine (0.15 mmol, 37 mg,) in aqueous ethanol solution (70 %, 1 mL). The mixture was stirred at 55 °C for 24–120 h. After the reaction was completed (TLC, $CHCl_3$:MeOH 9:1), solvents were evaporated under reduced pressure. The residue was dissolved in either chloroform and then purified by column chromatography ($CHCl_3$ to $CHCl_3$:MeOH 9:1) or water and purified by reverse phase column chromatography (C-18 cartridges, H_2O to H_2O:MeOH 5:1). After purification products were isollated in 39–91 % yields.

N^4-(2-hydroxyethyl)- 2'-deoxycytidine (53)

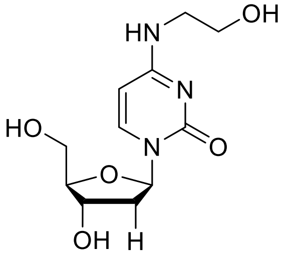

Yield 30 mg (75 %); UV (CH_3OH) λ_{max} 281 nm; ^{1}H NMR (400 MHz, DMSO-*d*6) δ 7.79 (t, *J* = 5.5 Hz, 1H), 7.73 (d, *J* = 7.5 Hz, 1H), 6.16 (dd, *J* = 10.3, 3.1 Hz, 1H), 5.80 (d, *J* = 7.5 Hz, 1H), 4.19 (dt, *J* = 6.1, 3.1 Hz, 1H), 3.77 – 3.74 (m, 1H), 3.57 – 3.52 (m, 2H), 3.49 (d, *J* = 5.8 Hz, 2H), 3.31 (q, *J* = 5.6 Hz, 2H), 2.17 – 2.02 (m, 1H), 1.93 (dd, *J* = 13.9, 6.7 Hz, 1H); ^{13}C NMR (100 MHz, DMSO-*d*6) δ 164.0, 155.5, 140.2, 95.2, 87.6, 85.3, 70.9, 61.8, 60.0, 43.1, 40.8; MS (ESI$^+$): *m/z* 271.90 $[M+H]^+$, 269.90 $[M-H]^-$.

N^4-(2-aminoethyl)-2'-deoxycytidine (54)

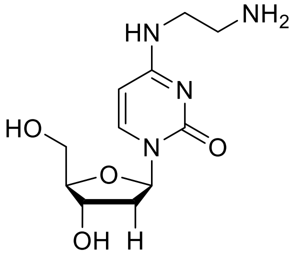

Yield 37 mg (91 %); UV (CH_3OH) λ_{max} 273 nm; ^{1}H NMR (400 MHz, DMSO-*d*6) δ 8.01–7.76 (m, 1H), 7.74 (d, *J* = 7.5 Hz, 1H), 6.21–6.10 (m, 1H), 5.77 (d, *J* = 7.5 Hz, 1H), 4.20 (dt, *J* = 6.1, 3.2 Hz, 1H), 3.80 – 3.71 (m, 3H), 3.60–3.48 (m, 2H), 3.25 (s, 2H), 2.67 (t, *J* = 6.3 Hz, 2H), 2.15–2.02 (m, 1H), 1.98–1.88 (m, 1H); ^{13}C NMR (100 MHz, DMSO-*d*6) δ 163.9, 155.5, 140.1, 95.2, 87.6, 85.3, 70.8, 61.8, 43.5, 41.1, 40.7; MS (ESI$^+$): *m/z* 270.95 $[M+H]^+$, 268.95 $[M-H]^-$.

N^4-(4-morpholinyl)- 2'-deoxyuridine (55)

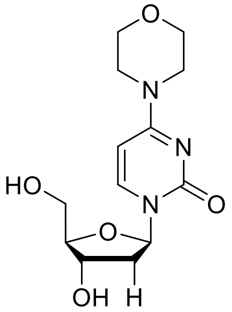

Yield 36 mg (81 %); UV (CH_3OH) λ_{max} 286 nm; ^{1}H NMR (400 MHz, DMSO-*d*6) δ 7.96 (d, *J* = 7.8 Hz, 1H), 6.18 (d, *J* = 7.8 Hz, 1H), 6.14 (t, *J* = 6.6 Hz, 1H), 5.22 (bs, 1H), 5.04 (bs, 1H), 4.27–4.17 (m, 1H), 3.84–3.76 (m, 1H), 3.68 – 3.54 (m, 10H), 2.21 – 2.10 (m, 1H), 2.01 – 1.91 (m, 1H); ^{13}C NMR (100 MHz, DMSO-*d*6) δ 163.1, 154.8, 142.2, 91.6, 87.8, 85.5, 70.7, 66.2, 66.0, 61.7, 41.0; MS (ESI$^+$): *m/z* 297.90 $[M+H]^+$, 295.90 $[M-H]^-$.

5-fluoro-N^4-(4-morpholinyl)-2'-deoxy uridine (56)

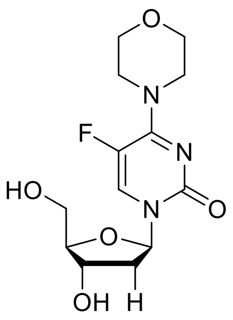

Yield 26 mg (55 %); UV (CH_3OH) λ_{max} 293 nm; ^{1}H NMR (400 MHz, DMSO-d_6) δ 8.22 (d, *J* = 10.2 Hz, 1H), 6.07 (t, *J* = 6.4 Hz, 1H), 5.22 (bs, 2H), 4.30–4.18 (m, 1H), 3.81 – 3.76 (m, 1H), 3.73–3.70 (m, 2H), 3.68 – 3.64 (m, 4H), 3.62 – 3.56 (m, 4H), 2.18 – 2.11 (m, 1H), 2.04–1.97 (m, 1H); ^{13}C NMR (100 MHz, DMSO-d_6) δ 154.5, 152.7, 138.4, 136.0, 128.7, 87.9, 85.7, 70.4, 66.4, 61.4, 46.4, 45.4, 41.0; MS (ESI$^+$): *m/z* 315.85 $[M+H]^+$.

N^4-*n*-hexyl2'-deoxycytidine (57)

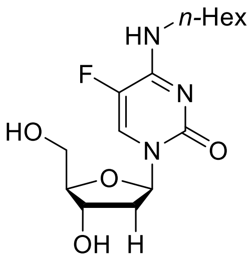

Yield 42 mg (90 %); UV (CH_3OH) λ_{max} 282 nm; ^{1}H NMR (400 MHz, DMSO-d_6) δ 7.65 (d, *J* = 7.5 Hz, 1H), 7.61 (t, *J* = 5.5 Hz, 1H), 6.09 (dd, *J* = 7.3, 6.2 Hz, 1H), 5.66 (d, *J* = 7.5 Hz, 1H), 5.12 (d, *J* = 4.2 Hz, 1H), 4.92 (t, *J* = 10.6, 4.2 Hz, 1H), 4.21–4.06 (m, 1H), 3.76–3.61 (m, 1H), 3.55–3.38 (m, 2H), 3.22 – 3.07 (m, 2H), 2.11–1.95 (m, 1H), 1.93 – 1.79 (m, 1H), 1.45 – 1.35 (m, 2H), 1.25–1.17 (m, 6H), 0.79 (t, *J* = 4.8 Hz, 3H); ^{13}C NMR (100 MHz, DMSO-d_6) δ 163.7, 155.6, 140.1, 95.1, 87.6, 85.3, 70.9, 61.9, 40.7, 31.5, 29.0, 26.6, 22.5, 14.4; MS (ESI$^+$): *m/z* 312.00 $[M+H]^+$, 310.00 $[M-H]^-$.

4-(indolin-1-yl)-2'-deoxyuridine (58)

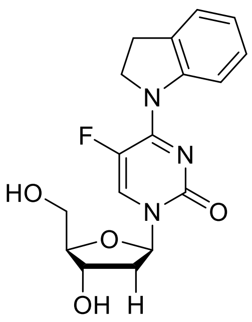

Yield 26 mg (53 %); UV (CH_3OH) λ_{max} 320 nm; ^{1}H NMR (400 MHz, DMSO-d_6) δ 8.54 (s, 2H), 8.13 (d, *J* = 7.6 Hz, 2H), 7.26 (d, *J* = 7.3 Hz, 2H), 7.20 (t, *J* = 7.7 Hz, 2H), 7.00 (td, *J* = 7.4, 0.8 Hz, 2H), 6.20 (t, *J* = 6.5 Hz, 2H), 6.16 (d, *J* = 7.3 Hz, 1H), 5.25 (d, *J* = 4.2 Hz, 2H), 5.06 (t, *J* = 5.2 Hz, 2H), 4.24 (td, *J* = 7.3, 3.6 Hz, 2H), 4.10 (t, *J* = 8.7 Hz, 2H), 3.83 (q, *J* = 3.6 Hz, 2H), 3.67–3.51 (m, 5H), 3.19 (t, *J* = 8.5 Hz, 2H), 2.28–2.16 (m, *J* = 13.2, 6.0, 3.6 Hz, 2H), 2.07–1.95 (m, 2H); ^{13}C NMR (100 MHz, DMSO-d_6) δ 161.2, 154.1, 143.2, 142.4, 133.1, 127.1, 125.2, 123.5, 94.0, 88.0, 85.9, 70.7, 61.6, 49.0, 40.6, 27.2; MS (ESI$^+$): *m/z* 329.90 $[M+H]^+$, 327.90 [M-H]

N^4-(2,3,4,5,6-pentahydroxyhexyl)- 2'-deoxycytidine (59)

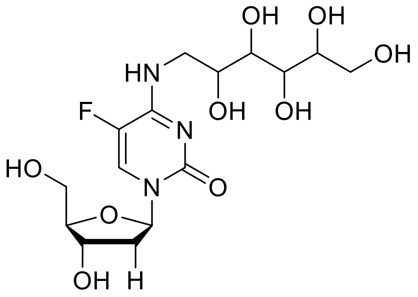

Yield 35 mg (60 %); UV (CH_3OH) λ_{max} 282 nm; ^{1}H NMR (400 MHz, DMSO-d_6) δ = 7.73 (d, *J* = 7.5 Hz, 1H), 7.70 (t, *J* = 5.6 Hz, 1H), 6.18 – 6.13 (m, 1H), 5.85 (d, *J* = 7.5 Hz, 1H), 5.19 (d, *J* = 4.1 Hz, 1H), 5.08 – 4.85 (m, 2H), 4.49 (d, *J* = 5.2 Hz, 1H), 4.43 (t, *J* = 5.8 Hz, 2H), 4.36 (t, *J* = 5.4 Hz, 1H), 4.22–4.16 (m, 1H), 3.76 (dd, *J* = 6.9, 3.7 Hz, 1H), 3.73– 3.65 (m, 1H), 3.62–3.51 (m, 3H), 3.52–3.43 (m, 3H), 3.40 (dd, *J* = 9.5, 4.0 Hz, 1H), 3.29 – 3.13 (m, 1H), 2.09 (ddd, *J* = 13.1, 5.9, 3.6 Hz, 1H), 2.02 – 1.83 (m, 1H); ^{13}C NMR (100 MHz, DMSO-d_6) δ 164.1, 155.4, 140.1, 95.3, 87.5, 85.2, 72.5, 72.1, 72.0, 70. 9, 70.1, 63.8, 61.8, 43.7, 40.7; MS (ESI$^+$): *m/z* 391.85 $[M+H]^+$.

N^4-((1*H*-indol-6-yl)methyl)- 2'-deoxycytidine (60)

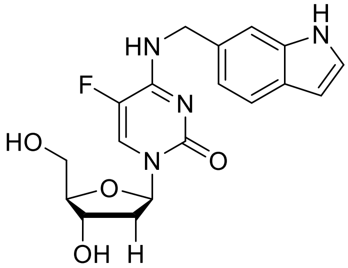

Yield 35 mg (65 %); UV (CH_3OH) λ_{max} 282 nm; ^{1}H NMR (400 MHz, DMSO-d_6) δ 11.05 (s, 1H), 8.13 (t, *J* = 5.7 Hz, 1H), 7.77 (d, *J* = 7.5 Hz, 1H), 7.48 (d, *J* = 8.1 Hz, 1H), 7.32 (s, 1H), 7.31–7.29 (m, 1H), 6.95 (dd, *J* = 8.1, 1.3 Hz, 1H), 6.41–6.36 (m, 1H), 6.22–6.15 (m, 1H), 5.83 (d, *J* = 7.5 Hz, 1H), 5.20 (d, *J* = 4.2 Hz, 1H), 4.97 (t, *J* = 5.3 Hz, 1H), 4.56 (d, *J* = 5.7 Hz, 2H), 4.28–4.14 (m, *J* = 3.3 Hz, 1H), 3.83–3.66 (m, 1H), 3.61 – 3.48 (m, 2H), 2.15 – 2.05 (m, 1H), 2.00 – 1.90 (m, 1H); ^{13}C NMR (100 MHz, DMSO-d_6) δ 163.6, 155.5, 140.3, 136.5, 132.0, 127.2, 125.8, 120.2, 110.8, 101.3, 95.1, 87.6, 85.2, 79.7, 70.9, 61.8, 56.5, 44.2; MS (ESI$^+$): *m/z* 357.00 $[M+H]^+$, 355.00 $[M-H]^-$.

Synthesis of n-pentyl carbamate

It was used as a standard for analysis of hydrolysis products after the incubation of capecitabine with CDA. A mixture of urea (1.5 g, 25 mmol), n-pentanol (2.97 ml, 27.5 mmol) and Cu(II)acetate (46 mg, 0.25 mmol) was heated for 3-4 hours at 150 °C temperature. Then, the mixture was cooled to room temperature. The formed product was extracted to chloroform (120 mL) and washed with water (3x50 mL). Organic layer was dried with anhydrous Na_2SO_4 and filtered. The filtrate was concentrated under reduced pressure to afford 2.8 g (77 % yield) of n-pentyl carbamate. The structure of carbamate was approved by NMR spectroscopy and HPLC-MS and GC-MS.

n-Pentyl carbamate. Yield 2.8 g (77 %).

MS (ESI+):m/z 132.05 [M+H]+.

1H NMR (400 MHz, $CDCl_3$): δ = 4.74 (bs, 2H, NH_2), 4.07 (t, J = 6.8 Hz, 2H, CH_2O), 1.69–1.60 (m, 2H, CH_2), 1.41–1.33 (m, 4H, $2CH_2$), 0.99–0.91 (m, 3H, CH_3).

13C NMR (101 MHz, $CDCl_3$): δ = 157.19, 65.35, 28.61, 27.96, 22.34, 13.97.

Figures

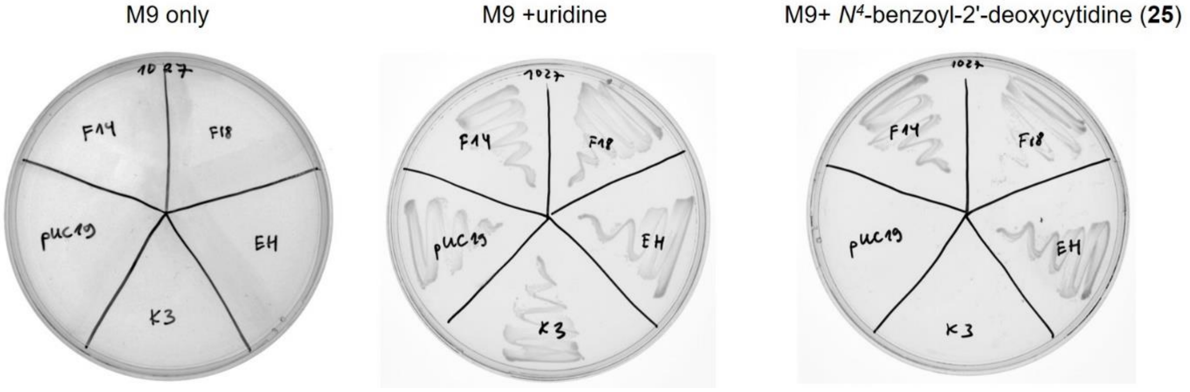

Figure S1. **Growth of the selected clones on the M9 medium after 2 days of incubation at 37 °C**. M9 only: negative control (no uridine source); M9 + uridine: positive control; M9 + N^4-benzoyl-2'-deoxycytidine (**25**) – compound as a sole source of uridine; pUC19: *E. coli* DH10B *ΔpyrFEC cdd*::Km (uridine and cytidine deaminase auxotroph) with empty pUC19 vector (control); F14: DH10B *ΔpyrFEC cdd*::Km + pUC19_CDA_F14 plasmid; F14: DH10B *ΔpyrFEC cdd*::Km + pUC19_CDA_EH plasmid; F18: DH10B *ΔpyrFEC cdd*::Km + pUC19_CDA_F18 plasmid; K3: DH10B *ΔpyrFEC cdd*::Km + pUC19_K3_Am (amidohydrolase) plasmid (21);

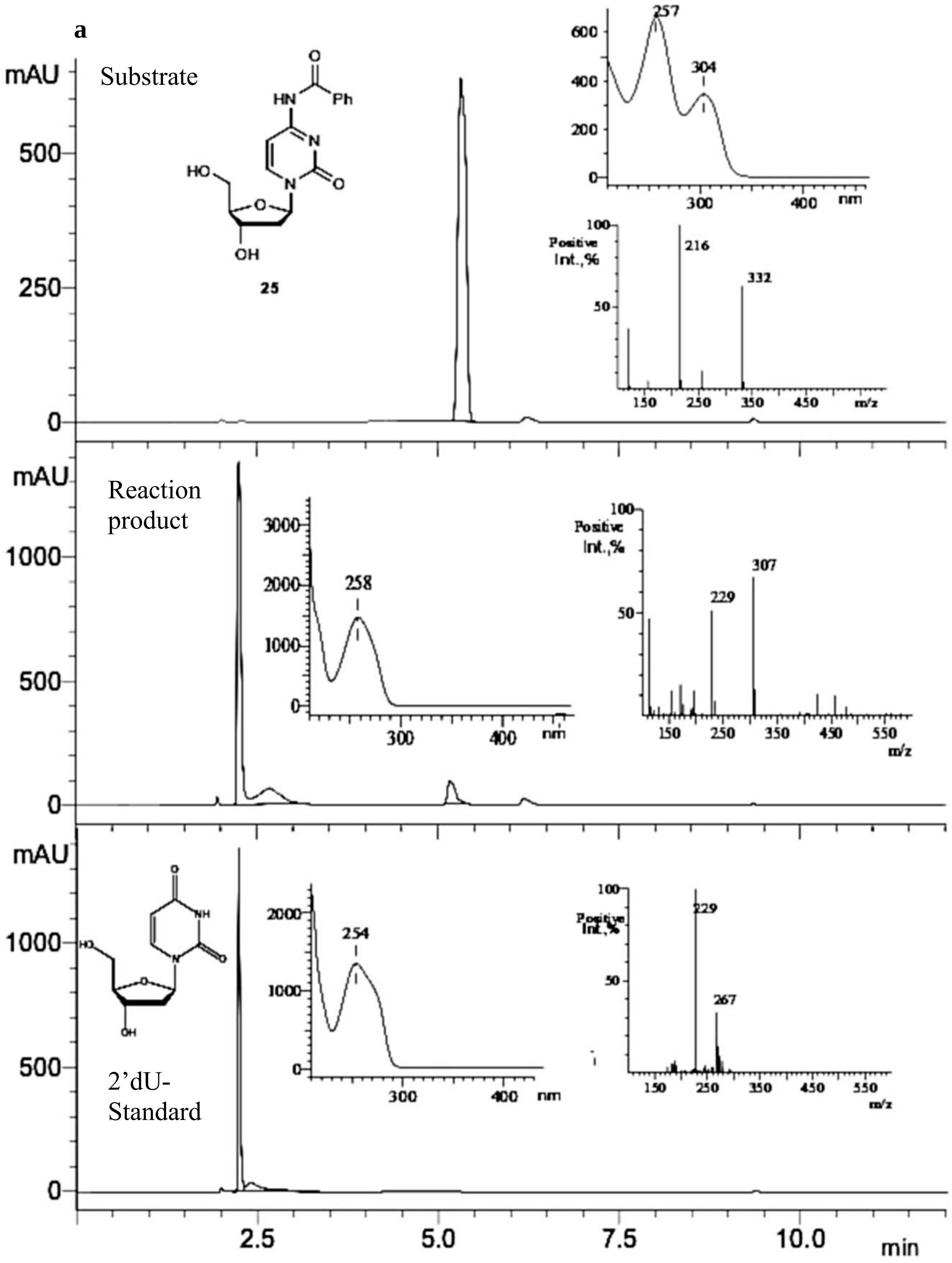

a
Substrate
25
Reaction product
2'dU-Standard
mAU
min
257
304
216
332
258
229
307
254
267
Positive Int.,%
nm
m/z

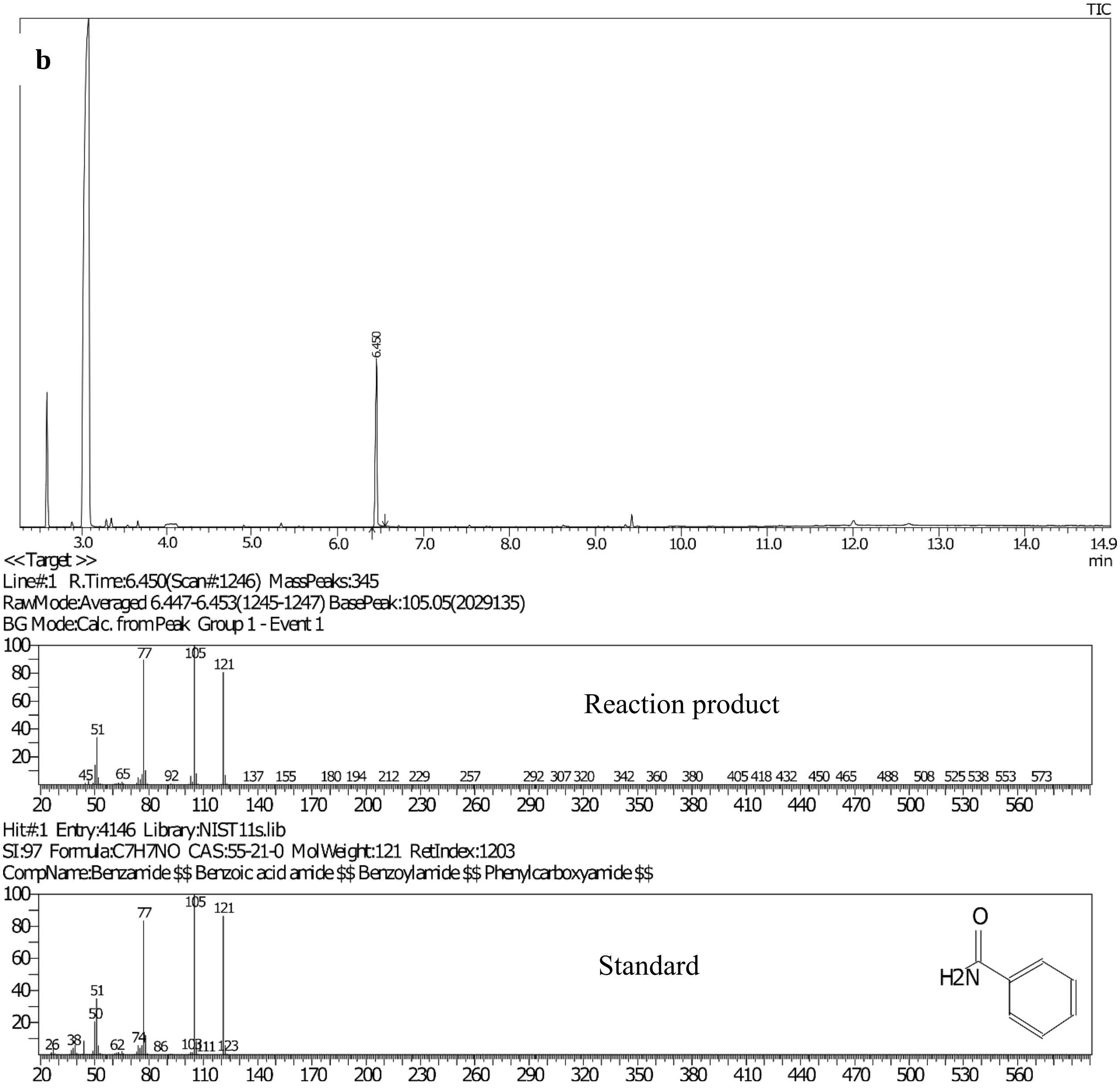

Figure S2. HPLC-MS and GC-MS analysis of the activity of CDA_F14 after incubation with *N*[4]-benzoyl-2'-deoxycytidine (**25**). **a**: HPLC-MS spectrum of nucleosides, **b**: GC-MS spectrum of benzamide.

a

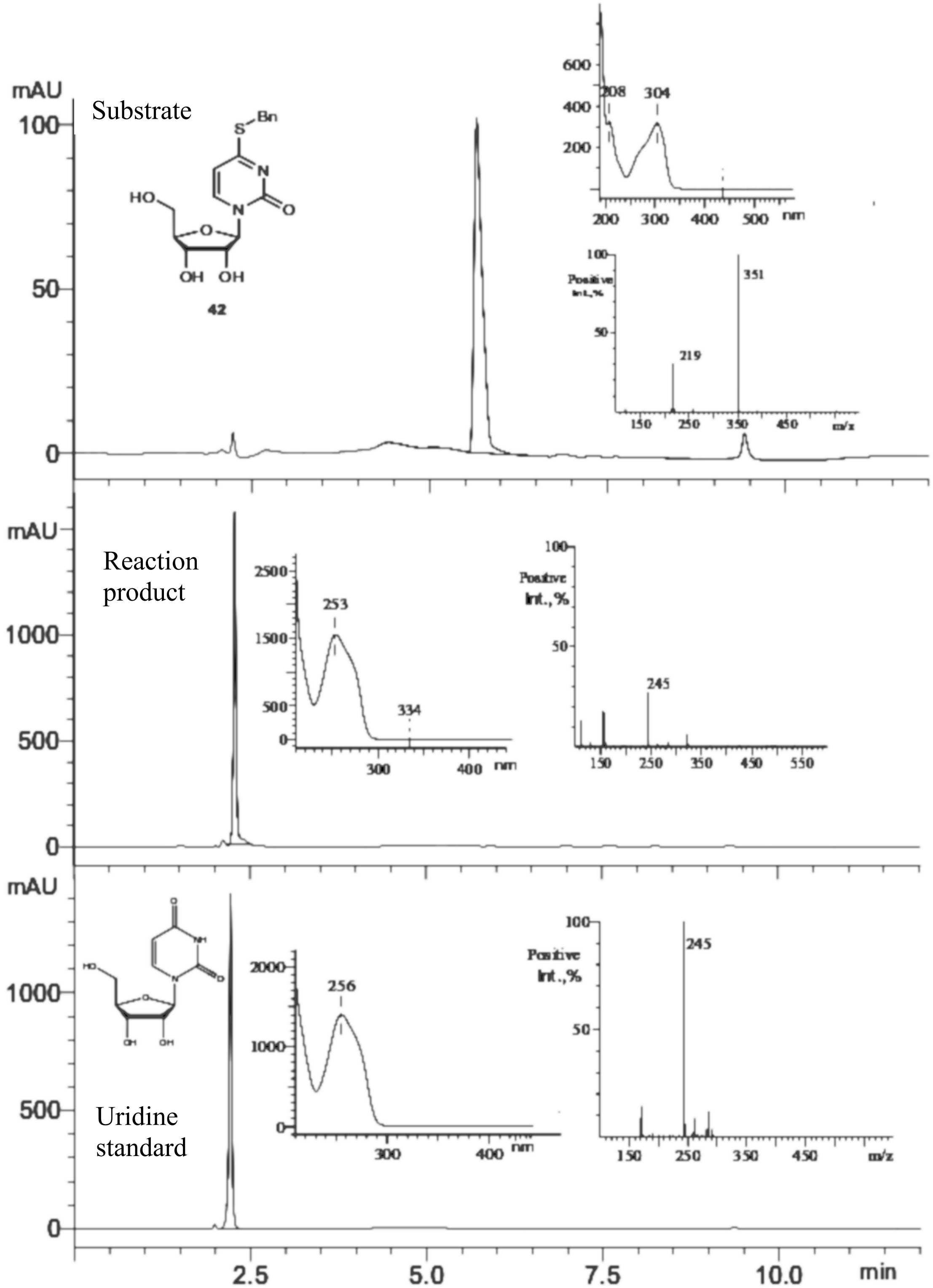

mAU
Substrate
S
Bn
N
HO
N
O
O
OH OH
42
600
400
200
208
304
200 300 400 500 nm
100
Positive
Int.,%
50
351
219
150 250 350 450 m/z
100
50
0
mAU
Reaction
product
2500
1500
500
0
253
334
300 400 nm
100
Positive
Int.,%
50
245
150 250 350 450 550
1000
500
0
mAU
HO
OH OH
2000
1000
0
256
300 400 nm
100
Positive
Int.,%
50
245
150 250 350 450 m/z
Uridine
standard
1000
500
0
2.5 5.0 7.5 10.0 min

b

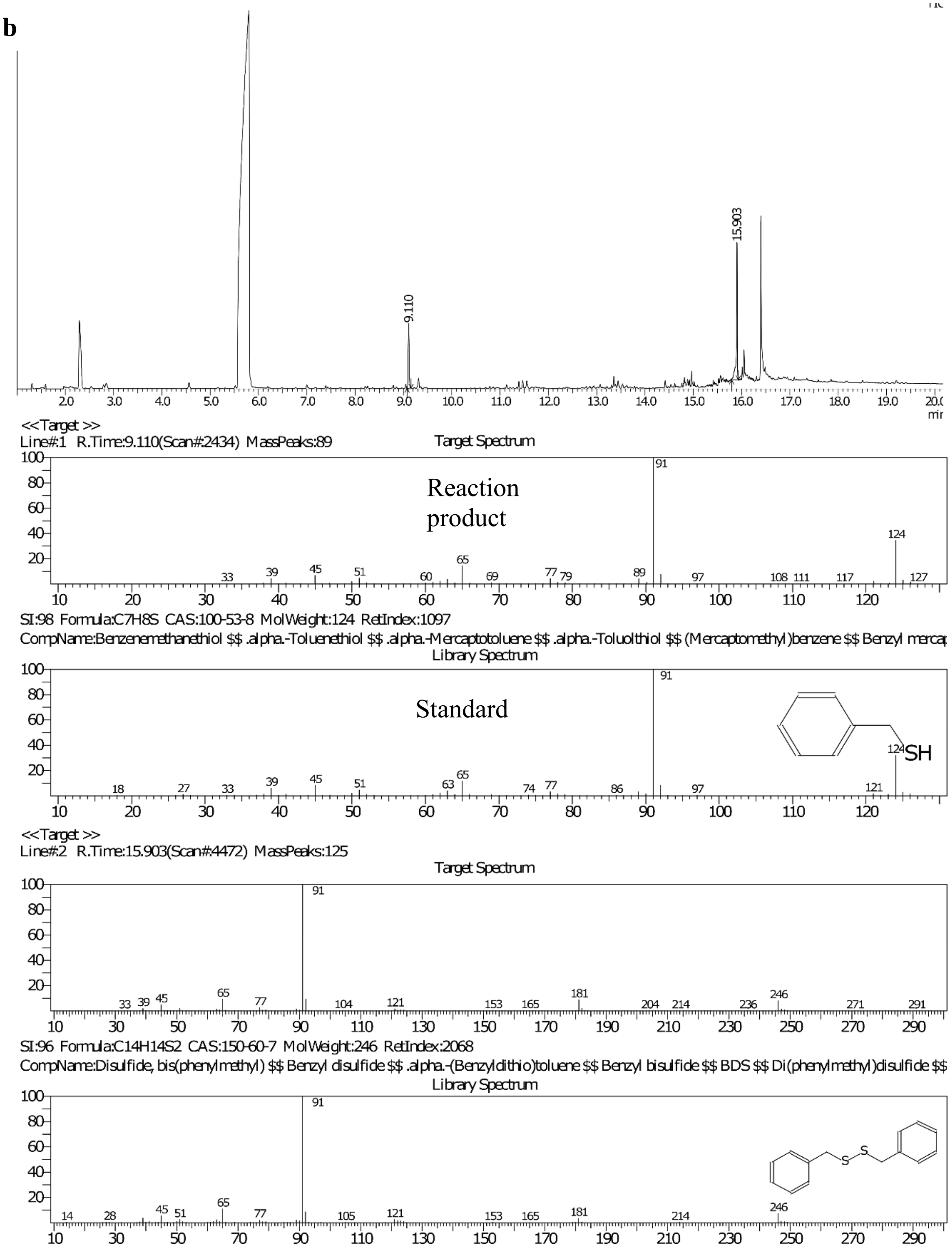

Figure S3. HPLC-MS and GC-MS analysis of the activity of CDA_F14 after incubation with *S*[4]-benzylthiouridine (42); **a**: HPLC-MS spectrum of nucleosides, **b**: GC-MS spectrum of benzyl mercaptan.

a

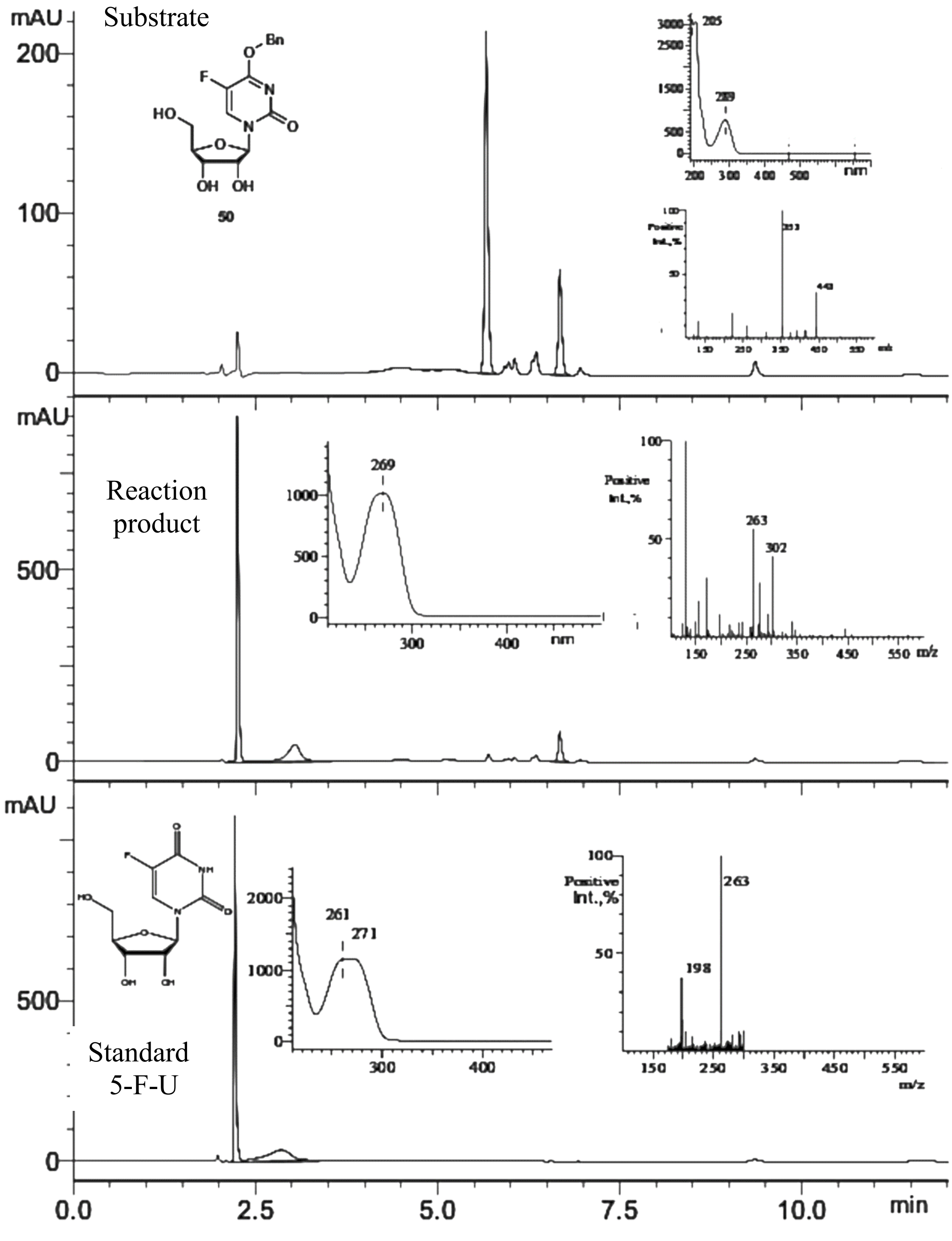

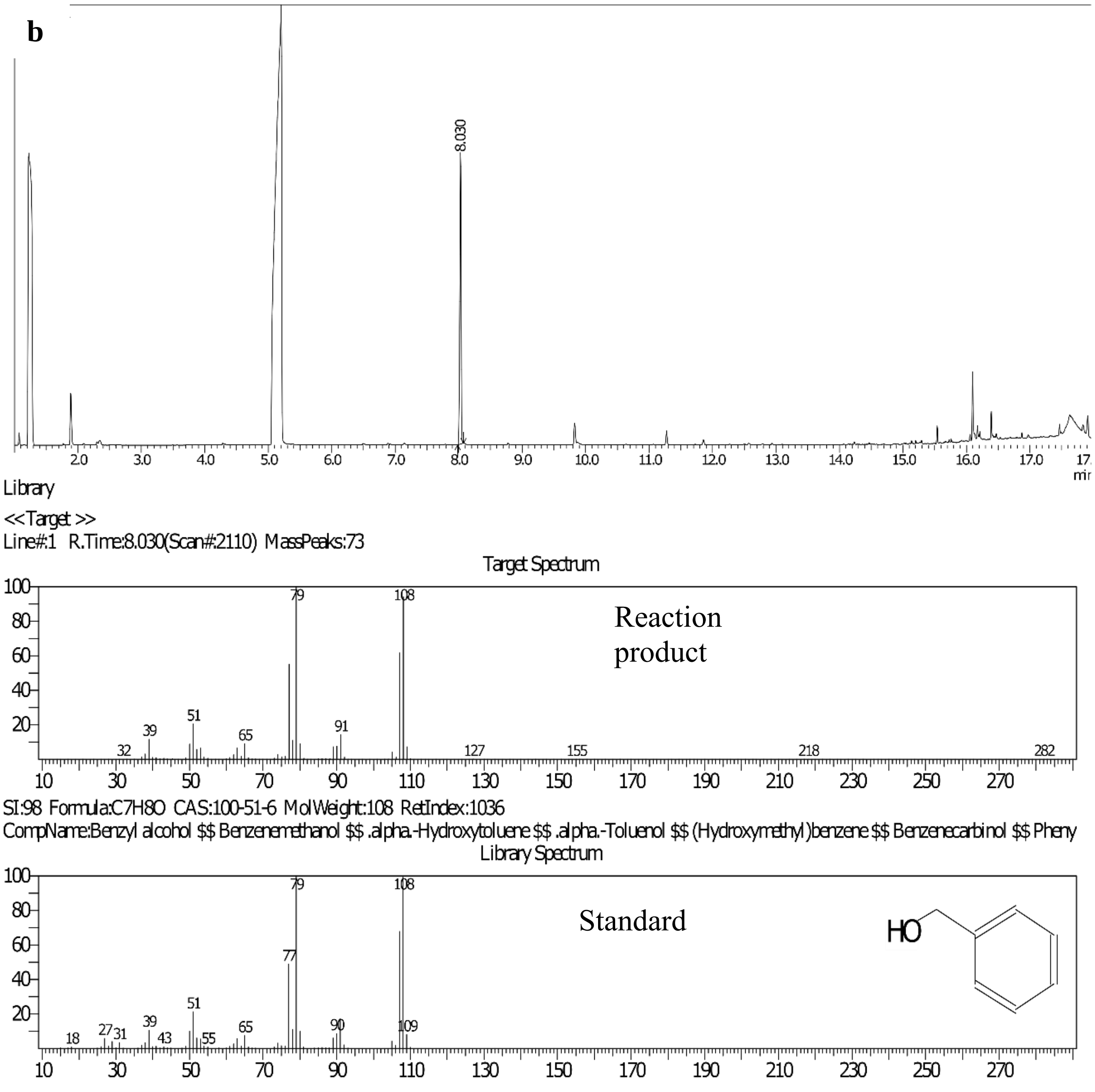

Figure S4. HPLC-MS and GC-MC analysis of the activity of CDA_F14 after incubation with 4-benzyloxy-5-fluoro-uridine (50); a: HPLC-MS spectrum of nucleosides, **b**: GC-MS spectrum of benzyl alcohol.

a

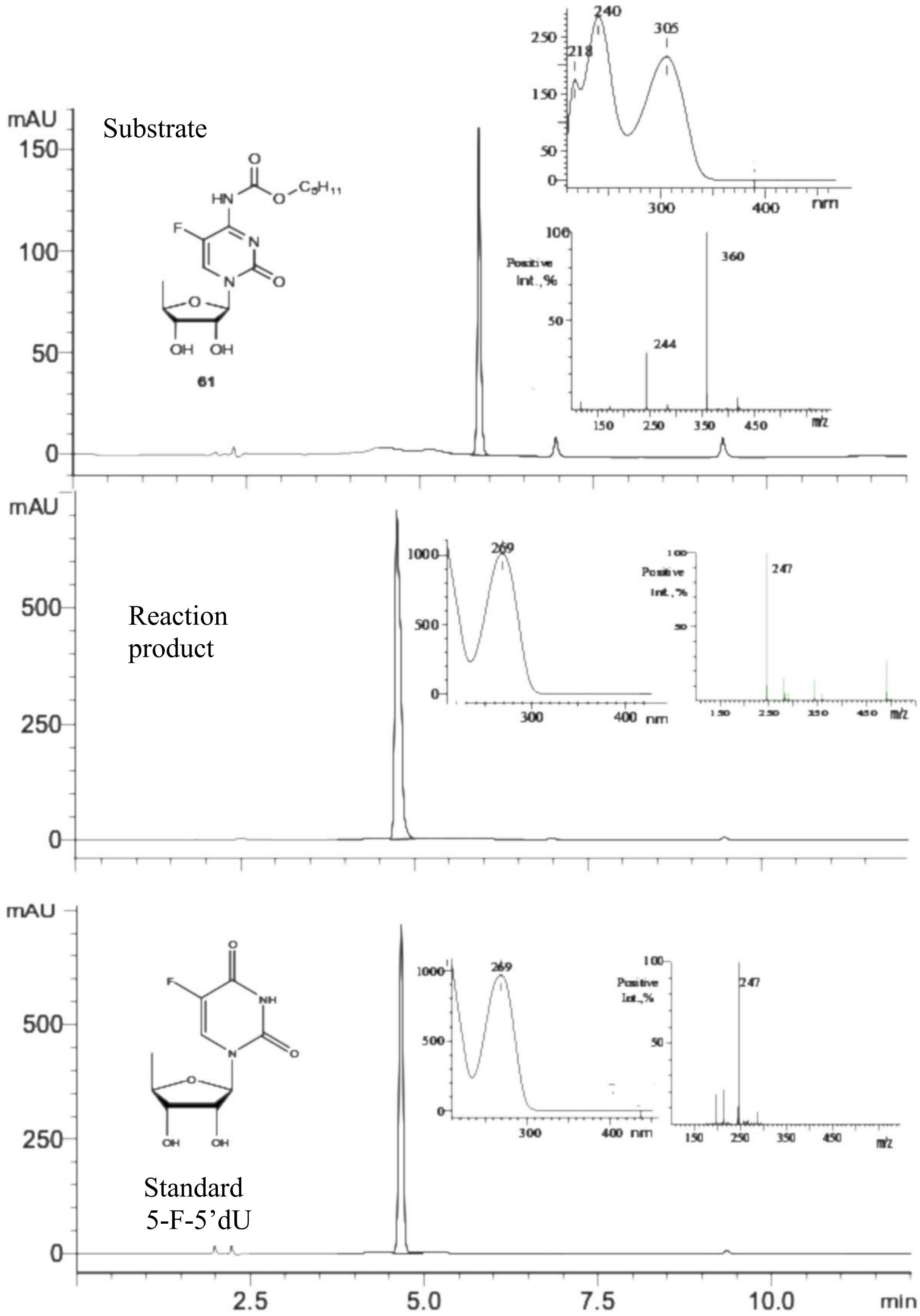
mAU
Substrate
61
218
240
305
nm
Positive Int.,%
360
244
m/z
Reaction product
269
247
Standard 5-F-5'dU
min
2.5
5.0
7.5
10.0

b

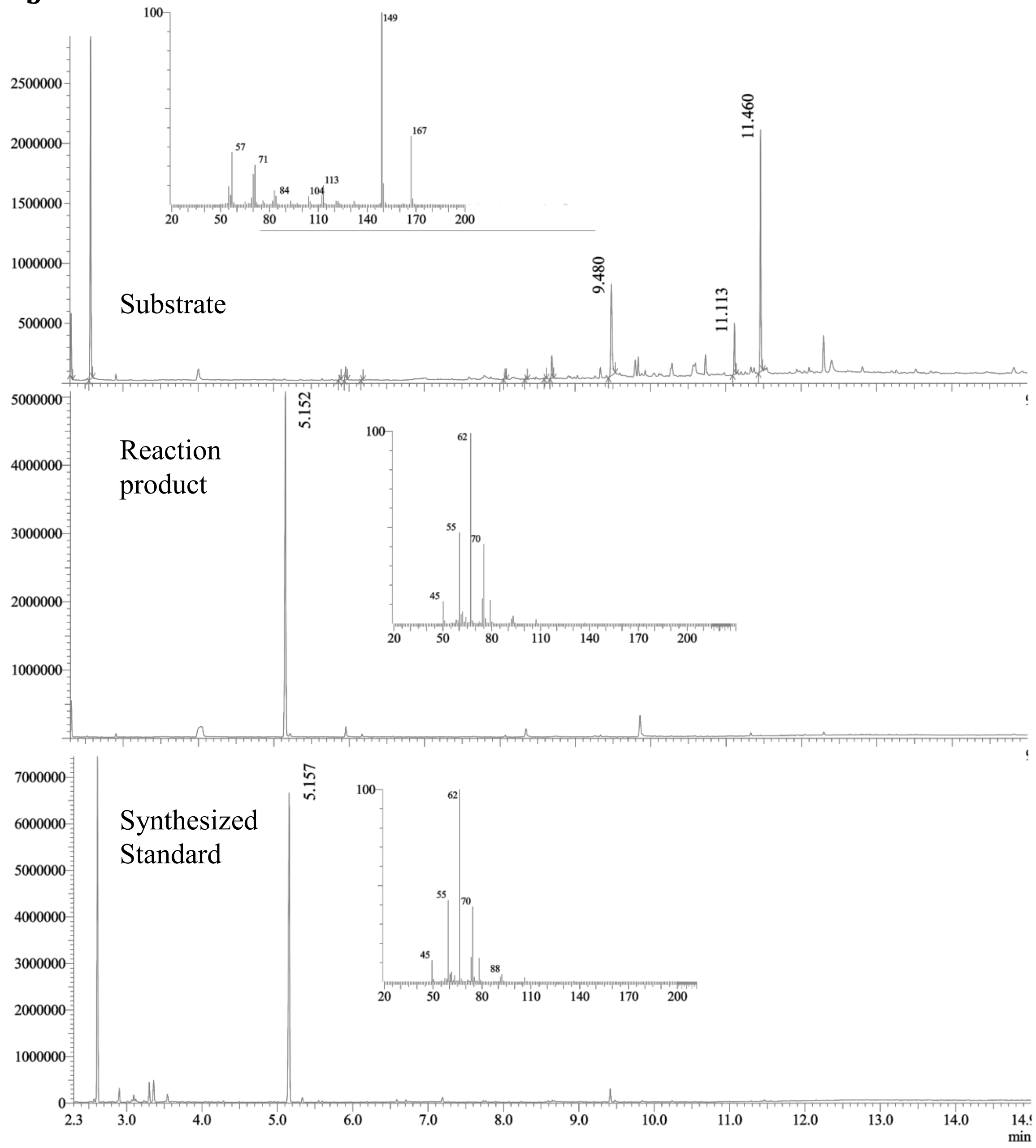

Figure S5. HPLC-MS and GC-MS analysis of the activity of CDA_F14 after incubation with capecitabine (61) (5’-deoxy-5-fluoro-*N*[4]-pentyloxycarbonylcytidine); **a**: HPLC-MS spectrum of nucleosides, **b**: GC-MS spectrum of penthyl carbamate (synthesis Supl.12-13 p).

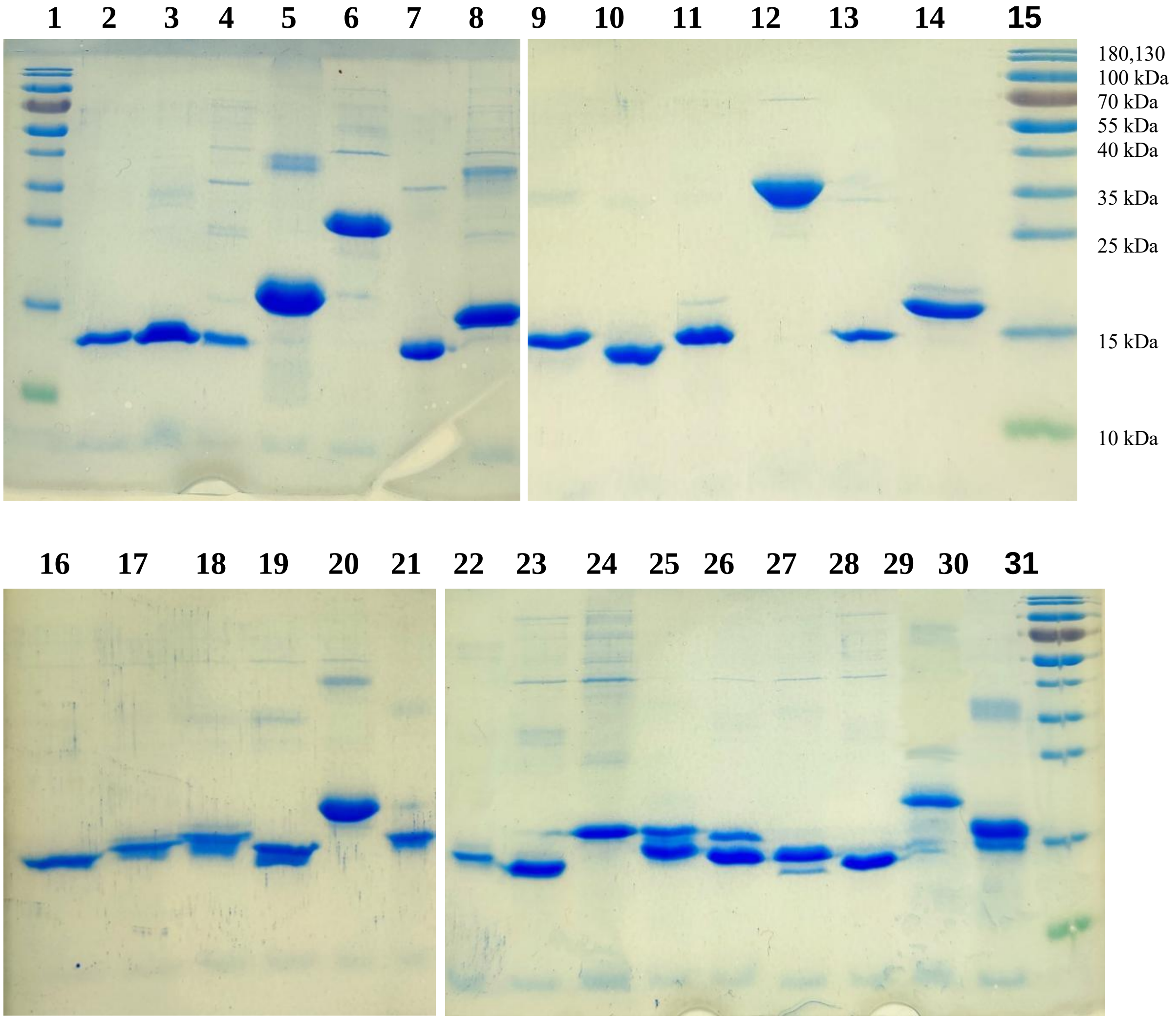

Figure S6. SDS-PAGE analysis of the purified cytidine deaminases. Proteins were separated by SDS-PAGE (14% separating and 4.0% stacking gels). Gels were developed in Coomassie Brilliant Blue G-250 dye. **1, 15, 31** – PageRuler Prestained Protein Ladder (180-10 kDa), **2** – CDA_F14, **3** – CDA_F18, **4** – CDA_MTU, **5** – CDA_E9, **6** – CDA_V86, **7** – CDA_V52, **8** – CDA_Pco, **9** –CDA_ EH, **10** – CDA_Lsp, **11** – CDA_Bsu, **12** – CDA_ Eco, **13** – CDA_M1, **14** – CDA_E5, **16** – CDA_ Ppo, **17** – CDA_Hfi, **18** – CDA_Smo, **19** – CDA_Tar, **20** – CDA_Pin, **21** – CDA_E8, **22** – CDA_E10, **23** – CDA_ V4, **24** – CDA_V20, **25** – CDA_30, **26** – CDA_V47, **27** – CDA_82, **28** – CDA_106, **29** – CDA_M2, **30** – CDA_M13.

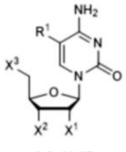

1-8, 11-15

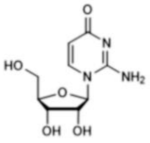

9

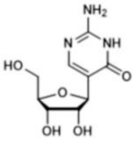

10

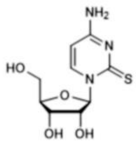

16

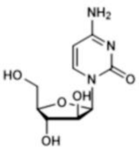

17

Substrate	-R^1	-X^1	-X^2	-X^3
**1**	H	H	OH	OH
**2**	H	OH	OH	OH
**3**	H	H	H	OH
**4**	H	H	OH	H
**5**	H	OH	NH_2	OH
**6**	Me	OH	OH	OH
**7**	H	OMe	OH	OH
**8**	F	OH	OH	OH
**11**	*n*Pr	H	OH	OH
**12**	CH_2OH	H	OH	OH
**13**	CH_2OH	OH	OH	OH
**14**	Me	H	OH	OH
**15**	OH	H	OH	OH

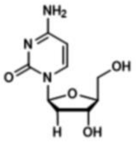

18

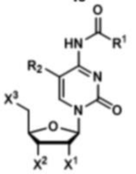

19-36

Compound number	-R^1	-R^2	-X^1	-X^2	-X^3
19	Me	H	OH	OH	OH
20	Ph	H	OH	OH	OH
21	Ph	Me	OH	OH	OH
22	Me	H	H	OH	OH
23	*i*Pr	H	H	OH	OH
24	C_5H_{11}	H	H	OH	OH
25	Ph	H	H	OH	OH
26	3-pyridyl	H	H	OH	OH
27	3-acetylphenyl	H	H	OH	OH
28	4-acetylphenyl	H	H	OH	OH
29	2-benzoylphenyl	H	H	OH	OH
30	3-benzoylphenyl	H	H	OH	OH
31	4-benzoylphenyl	H	H	OH	OH
32	Me	H	H	OH	DMTr
33	Ph	H	H	OH	*O*-levulinoyl
34	Ph	H	H	O-levulinoyl	OH
35	Ph	H	H	OAc	OH
36	Ph	H	H	N_3	OH

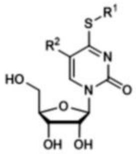

37-46

Substrate	-R^1	-R^2
**37**	Me	H
**38**	Et	H
**39**	*n*Pr	H
**40**	*i*Pr	H
**41**	*i*Bu	H
**42**	Bn	H
**43**	Me	F
**44**	Et	F
**45**	Bn	F
**46**	Ph	F

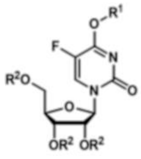

47-50

Substrate	-R^1	-R^2
**47**	Me	H
**48**	*n*Bu	H
**49**	*n*Bu	Ac
**50**	Bn	H

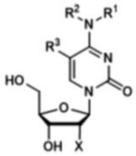

51-60

Compound number	-R^1	-R^2	-R^3	-X
51	Me	H	H	OH
52	Me	Me	H	OH
53	2-hydroxyethyl	H	H	H
54	2-aminoethyl	H	H	H
55	4-morpholinyl		H	H
56	4-morpholinyl		F	H
57	*n*Hex	H	H	H
58	1-indolinyl		H	H
59	2,3,4,5,6-pentahydroxyhexyl	H	H	OH
60	(1*H*-indol-6-yl)methyl	H	H	H

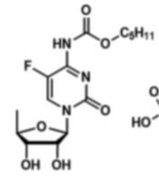

61

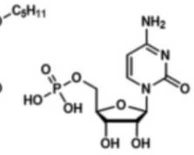

62

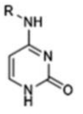

73-75

Compound number	-R
73	H
74	Acetyl
75	Benzoyl

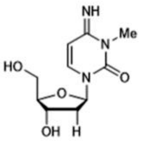

76

Figure S7. Compounds tested as substrates for cytidine deaminases.

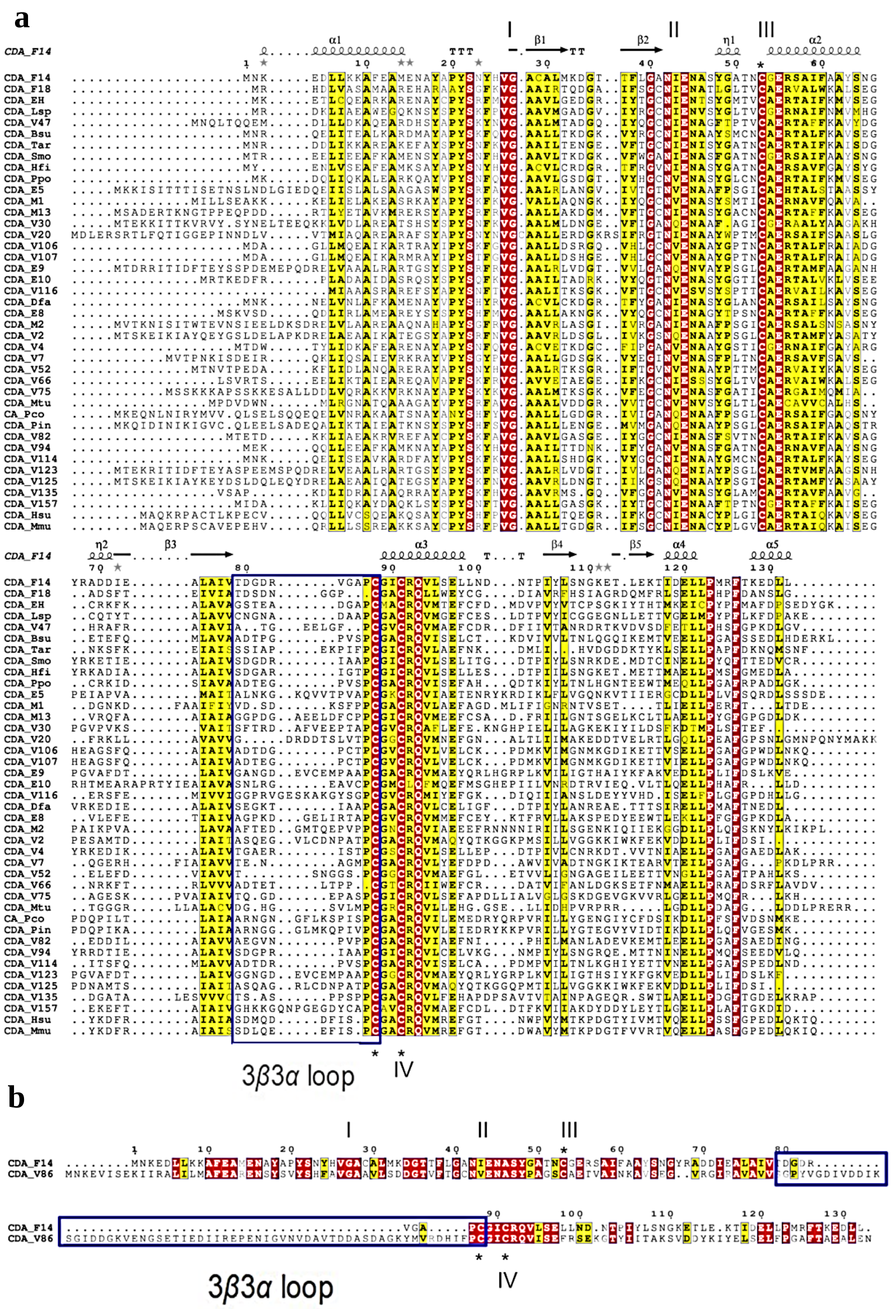

Figure S8. **The alignment of amino acid sequences of tested CDAs**. CDA_V86 had a prolonged middle part of the protein uncharacteristic for other CDAs (**a**), therefore was shown separately (**b**). Identical conserved residues and strongly similar are shown in red in yellow background, respectively. The secondary structure elements displayed above sequence: α helices and β strands, turns (TT) and 3_{10}-helix (η). Symbols I, II, III and IV indicate conservative regions, asterisk***** – catalytic cysteines and the non-conservative β3α3 loop is drawn in blue box. The sequence alignment was performed using ClustalW in MEGA X (42). The visualisation performed by ESPript 3.0 server (68).

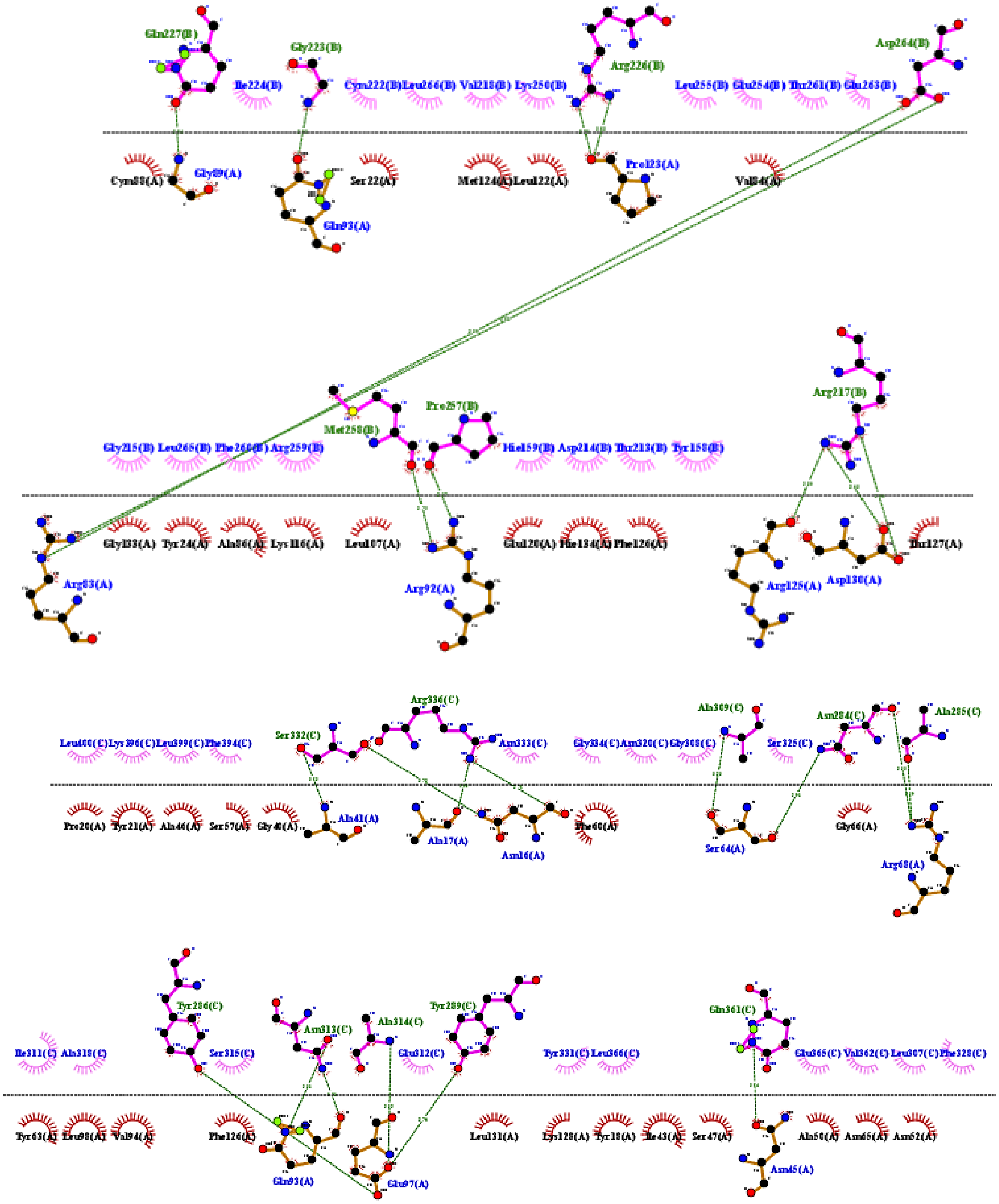

Gln227(B)
Gly223(B)
Ile224(B)
Cym222(B)
Leu266(B)
Val218(B)
Lys250(B)
Arg226(B)
Leu255(B)
Glu254(B)
Thr261(B)
Glu263(B)
Asp264(B)
Cym88(A)
Gly89(A)
Gln93(A)
Ser22(A)
Met124(A)
Leu122(A)
Pro123(A)
Val84(A)
Met258(B)
Pro257(B)
Arg217(B)
Gly215(B)
Leu265(B)
Phe266(B)
Arg259(B)
Hie159(B)
Asp214(B)
Thr213(B)
Tyr158(B)
Gly133(A)
Tyr24(A)
Ala86(A)
Lys116(A)
Leu107(A)
Glu120(A)
Phe134(A)
Phe126(A)
Thr127(A)
Arg83(A)
Arg92(A)
Arg125(A)
Asp130(A)
Arg336(C)
Ser332(C)
Ala309(C)
Asn284(C)
Ala285(C)
Leu400(C)
Lys396(C)
Leu399(C)
Phe394(C)
Asn333(C)
Gly334(C)
Asn320(C)
Gly308(C)
Ser325(C)
Pro20(A)
Tyr21(A)
Ala46(A)
Ser57(A)
Gly40(A)
Ala41(A)
Ala17(A)
Asn16(A)
Phe60(A)
Ser64(A)
Gly66(A)
Arg68(A)
Tyr286(C)
Asn313(C)
Ala314(C)
Tyr289(C)
Gln361(C)
Ile311(C)
Ala318(C)
Ser315(C)
Glu312(C)
Tyr331(C)
Leu366(C)
Glu365(C)
Val362(C)
Leu307(C)
Phe328(C)
Tyr63(A)
Leu98(A)
Val94(A)
Phe126(A)
Leu131(A)
Lys128(A)
Tyr18(A)
Ile43(A)
Ser47(A)
Ala50(A)
Asn65(A)
Asn62(A)
Gln93(A)
Glu97(A)
Asn45(A)

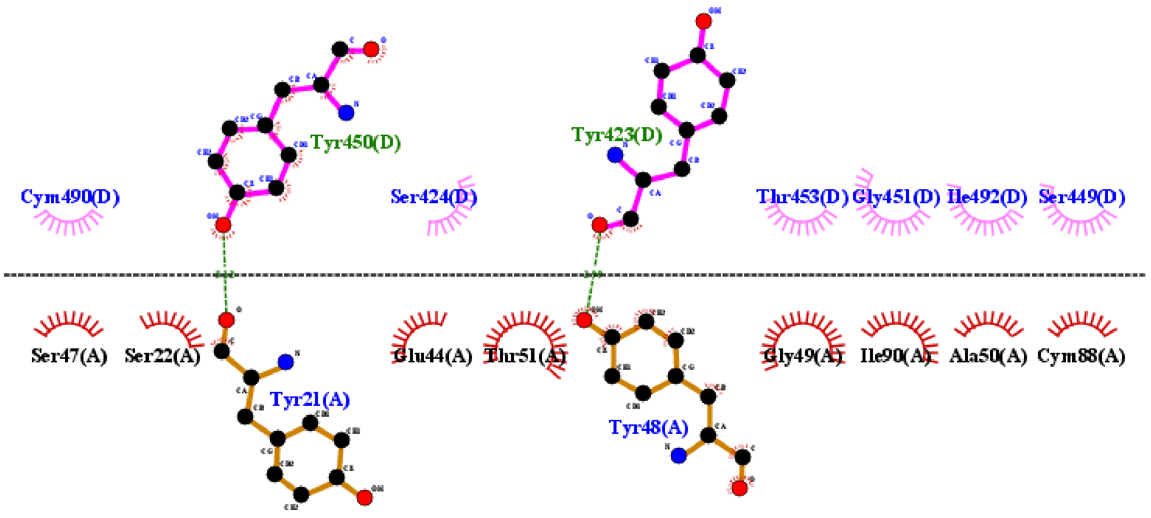

Figure S9. Schematic view of residues that constitute the interaction between subunits. Hydrogen bonds and coordination bonds are shown as dotted lines. Schematic view by using LigPlot+ program. The tetrameric structure was supported by formation of hydrogen bonds between Gln93, Gly89, Arg83, Pro123, Asp130, Arg92, Met124, and Arg125 (between A and B subunits); Ala41, Ser64, Ala17, Asn16, Arg68, Tyr18, Gln93, Glu97, Asn45, Ala46 (between A and C subunits); Tyr21 and Tyr48 residues (between A-D subunits). Several residues that constitute the interaction surfaces were highly conserved in the analysed CDAs. This applies to residues Tyr18, Tyr21, Asn45, Arg92 (CDA_E10 have Leu), Gln93, Glu97, Pro123, conservative also are Tyr48, Ala46, Ser64.

		CDA_F14	F14 del127-130	F14 del83-85	F14 F126A	F14 F126W	F14 T51G	F14 G85I	F14 R56L	F14 G81L	F14 G81L G85I	F14 SML	F14 HSI	F14 QQS	F14 HSSG	F14 CLYR	F14 C53H R56Q	F14 C88H C91H	F14 HQHH	CDA_Pco	CD_Pco G70T	CD_Pco I108A	CDA_Ppo	CD_Ppo C50T	CD_Ppo V82L	CDA_Tar	CD_Tar I85A	CDA_Lsp	CD_Lsp A82I
2'-dC	(1)	2	2	2	2	2	2	2	2	2	2	2	2	2	2	2	1	0	0	2	2	2	2	2	2	2	2	2	2
Cytidine	(2)	2	N/A	N/A	N/A	N/A	N/A	N/A	N/A	N/A	N/A	2	2	2	2	2	N/A	N/A	N/A	2	N/A	N/A	2	N/A	N/A	2	N/A	2	2
2',3'-ddC	(3)	2	0	0	0	0	0	0	N/A	N/A	N/A	0	0	0	0	0	N/A	N/A	N/A	0	0	0	1	2	0	2	2	0	0
2',5'-ddC	(4)	2	2	0	2	2	2	2	N/A	N/A	N/A	2	2	2	2	2	N/A	N/A	N/A	2	0	2	2	2	2	2	2	2	2
3'-amino 2',3'-ddC	(5)	2	N/A	N/A	0	1	1	N/A	N/A	N/A	N/A	N/A	N/A	N/A	N/A	N/A	N/A	N/A	N/A	1	N/A	N/A	1	N/A	N/A	1	N/A	1	N/A
5-methyl-C	(6)	2	N/A	N/A	N/A	N/A	N/A	N/A	N/A	N/A	N/A	N/A	N/A	N/A	N/A	N/A	N/A	N/A	N/A	2	N/A	N/A	2	N/A	N/A	2	N/A	2	2
2'-O-methyl-C	(7)	0	N/A	N/A	0	0	0	N/A	N/A	N/A	N/A	0	0	0	0	0	N/A	N/A	N/A	2	N/A	N/A	2	N/A	N/A	2	N/A	0	N/A
5-F-cytidine	(8)	2	N/A	N/A	N/A	N/A	N/A	N/A	N/A	N/A	N/A	N/A	N/A	N/A	N/A	N/A	N/A	N/A	N/A	2	N/A	N/A	2	N/A	N/A	2	N/A	2	2
isocytidine	(9)	0	N/A	N/A	N/A	N/A	N/A	N/A	N/A	N/A	N/A	N/A	N/A	N/A	N/A	N/A	N/A	N/A	N/A	0	N/A	N/A	0	N/A	N/A	0	N/A	0	0
pseudoisocytidine	(10)	1	N/A	N/A	N/A	N/A	N/A	N/A	N/A	N/A	N/A	N/A	N/A	N/A	N/A	N/A	N/A	N/A	N/A	2	N/A	N/A	2	N/A	N/A	2	N/A	2	N/A
5-propynyl-2'-dC	(11)	2	N/A	N/A	N/A	N/A	N/A	N/A	N/A	N/A	N/A	N/A	N/A	N/A	N/A	N/A	N/A	N/A	N/A	2	N/A	N/A	2	N/A	N/A	2	N/A	2	N/A
5-hydroxymethyl-2'-dC	(12)	2	N/A	N/A	N/A	N/A	N/A	N/A	N/A	N/A	N/A	N/A	N/A	N/A	N/A	N/A	N/A	N/A	N/A	1	N/A	N/A	1	N/A	N/A	2	N/A	2	N/A
5-hydroxymethyl-C	(13)	2	N/A	N/A	N/A	N/A	N/A	N/A	N/A	N/A	N/A	N/A	N/A	N/A	N/A	N/A	N/A	N/A	N/A	2	N/A	N/A	2	N/A	N/A	1	N/A	1	N/A
5-methyl-2'-dC	(14)	2	N/A	N/A	N/A	N/A	N/A	N/A	N/A	N/A	N/A	N/A	N/A	N/A	N/A	N/A	N/A	N/A	N/A	2	N/A	N/A	2	N/A	N/A	2	N/A	2	2
5-hydroxy-2'-dC	(15)	2	N/A	N/A	N/A	N/A	N/A	N/A	N/A	N/A	N/A	N/A	N/A	N/A	N/A	N/A	N/A	N/A	N/A	2	N/A	N/A	2	N/A	N/A	2	N/A	2	2
2-thio-cytidine	(16)	0	N/A	N/A	N/A	N/A	N/A	N/A	N/A	N/A	N/A	N/A	N/A	N/A	N/A	N/A	N/A	N/A	N/A	0	N/A	N/A	1	N/A	N/A	1	N/A	0	0
Cytosine β-D-arabinofuranoside	(17)	2	N/A	N/A	N/A	N/A	N/A	N/A	N/A	N/A	N/A	N/A	N/A	N/A	N/A	N/A	N/A	N/A	N/A	2	2	2	2	N/A	N/A	2	2	2	0
2'-deoxy-L-cytidine	(18)	2	N/A	N/A	N/A	N/A	N/A	N/A	N/A	N/A	N/A	N/A	N/A	N/A	N/A	N/A	N/A	N/A	N/A	2	N/A	N/A	2	N/A	N/A	2	N/A	2	2
N[4]-acetyl-C	(19)	2	N/A	N/A	N/A	N/A	N/A	N/A	N/A	N/A	N/A	N/A	N/A	N/A	N/A	N/A	N/A	N/A	N/A	0	N/A	N/A	1	N/A	N/A	1	N/A	2	N/A
N[4]-benzoyl-C	(20)	2	N/A	N/A	N/A	N/A	N/A	N/A	N/A	N/A	N/A	N/A	N/A	N/A	N/A	N/A	N/A	N/A	N/A	0	N/A	N/A	0	N/A	N/A	0	N/A	2	N/A
N[4]-Benzoyl-5-methyl-C	(21)	2	N/A	N/A	N/A	N/A	N/A	N/A	N/A	N/A	N/A	N/A	N/A	N/A	N/A	N/A	N/A	N/A	N/A	0	N/A	N/A	0	N/A	N/A	2	N/A	2	N/A
N[4]-acetyl-2'-dC	(22)	2	2	0	2	2	2	2	N/A	N/A	N/A	2	2	2	2	2	N/A	N/A	N/A	0	0	0	2	2	0	2	2	2	2
N[4]-isobutyryl-2'-dC	(23)	2	N/A	N/A	N/A	N/A	N/A	N/A	N/A	N/A	N/A	N/A	N/A	N/A	N/A	N/A	N/A	N/A	N/A	0	N/A	N/A	1	N/A	N/A	0	N/A	2	N/A
N[4]-hexanoyl-2'-dC	(24)	2	N/A	N/A	N/A	N/A	N/A	N/A	N/A	N/A	N/A	N/A	N/A	N/A	N/A	N/A	N/A	N/A	N/A	0	N/A	N/A	1	N/A	N/A	1	N/A	2	2
N[4]-benzoyl-2'-dC	(25)	2	2	0	2	2	1	2	2	2	2	2	2	2	2	2	0	0	0	0	0	0	2	2	2	2	2	2	2
N[4]-nicotinoyl-2'-dC	(26)	2	N/A	N/A	N/A	N/A	N/A	N/A	N/A	N/A	N/A	N/A	N/A	N/A	N/A	N/A	N/A	N/A	N/A	0	N/A	N/A	2	N/A	N/A	2	N/A	2	0
N[4]-(3-acetyl-benzoyl)-2'-dC	(27)	2	2	0	0	2	0	0	N/A	N/A	N/A	N/A	N/A	N/A	N/A	N/A	N/A	N/A	N/A	0	0	0	0	0	0	0	N/A	2	0
N[4]-(4-acetyl-benzoyl)-2'-dC	(28)	2	2	0	2	2	0	0	N/A	N/A	N/A	2	2	2	2	2	N/A	N/A	N/A	0	0	0	0	0	0	0	0	2	2
N[4]-(2-benzoyl-benzoyl)-2'-dC	(29)	0	0	0	0	0	0	0	N/A	N/A	N/A	0	0	0	0	0	N/A	N/A	N/A	0	0	0	0	0	0	0	0	0	0
N[4]-(3-benzoyl-benzoyl)-2'-dC	(30)	2	N/A	N/A	N/A	N/A	N/A	N/A	N/A	N/A	N/A	N/A	N/A	N/A	N/A	N/A	N/A	N/A	N/A	0	N/A	N/A	0	N/A	N/A	0	N/A	2	N/A
N[4]-(4-benzoyl-benzoyl)-2'-dC	(31)	0	0	0	0	0	0	0	N/A	N/A	N/A	N/A	N/A	N/A	N/A	N/A	N/A	N/A	N/A	0	0	0	0	0	0	0	0	2	0
N[4]-acetyl-2'-deoxy-5'-O-DMT-C	(32)	2	N/A	N/A	N/A	N/A	N/A	N/A	N/A	N/A	N/A	N/A	N/A	N/A	N/A	N/A	N/A	N/A	N/A	0	0	0	N/A	N/A	N/A	N/A	0	N/A	N/A
5'-Levulinyl-*N*[4]-benzoyl-2'-dC	(33)	0	N/A	N/A	N/A	N/A	N/A	N/A	N/A	N/A	N/A	N/A	N/A	N/A	N/A	N/A	N/A	N/A	N/A	0	N/A	N/A	0	N/A	N/A	0	N/A	0	0
3'-Levulinyl-*N*[4]-benzoyl-2'-dC	(34)	0	N/A	N/A	N/A	N/A	N/A	N/A	N/A	N/A	N/A	N/A	N/A	N/A	N/A	N/A	N/A	N/A	N/A	0	N/A	N/A	0	N/A	N/A	0	N/A	0	0
3'-acetyl-*N*[4]-benzoyl- 2'-dC	(35)	0	N/A	N/A	N/A	N/A	N/A	N/A	N/A	N/A	N/A	N/A	N/A	N/A	N/A	N/A	N/A	N/A	N/A	0	N/A	N/A	0	N/A	N/A	0	N/A	0	0
3'-azido-*N*[4]-benzoyl-2',3'-ddC	(36)	0	N/A	N/A	N/A	N/A	N/A	N/A	N/A	N/A	N/A	0	0	0	0	0	N/A	N/A	N/A	0	N/A	N/A	0	N/A	N/A	0	N/A	0	0
4-thio-methyl-U	(37)	2	N/A	N/A	N/A	N/A	N/A	N/A	N/A	N/A	N/A	N/A	N/A	N/A	N/A	N/A	N/A	N/A	N/A	0	2	2	1	2	2	1	2	2	1
4-thio-ethyl-U	(38)	2	N/A	N/A	N/A	N/A	N/A	N/A	N/A	N/A	N/A	N/A	N/A	N/A	N/A	N/A	N/A	N/A	N/A	0	N/A	N/A	0	N/A	N/A	0	N/A	2	2
4-thio-*n* -propyl-U	(39)	2	N/A	N/A	N/A	N/A	N/A	N/A	N/A	N/A	N/A	N/A	N/A	N/A	N/A	N/A	N/A	N/A	N/A	N/A	N/A	N/A	N/A	N/A	N/A	N/A	N/A	2	N/A
4-thio-*iso* -propyl-U	(40)	2	N/A	N/A	N/A	N/A	N/A	N/A	N/A	N/A	N/A	N/A	N/A	N/A	N/A	N/A	N/A	N/A	N/A	N/A	N/A	N/A	N/A	N/A	N/A	N/A	N/A	2	N/A
4-thio-*iso* -butyl-U	(41)	2	N/A	N/A	N/A	N/A	N/A	N/A	N/A	N/A	N/A	N/A	N/A	N/A	N/A	N/A	N/A	N/A	N/A	N/A	N/A	N/A	N/A	N/A	N/A	N/A	N/A	2	N/A
4-thio-benzyl-U	(42)	2	2	1	N/A	N/A	1	2	N/A	N/A	N/A	N/A	N/A	N/A	N/A	N/A	N/A	N/A	N/A	0	N/A	N/A	0	N/A	N/A	0	N/A	2	1
5-F-4-thio-methyl-U	(43)	2	N/A	N/A	N/A	N/A	N/A	N/A	N/A	N/A	N/A	N/A	N/A	N/A	N/A	N/A	N/A	N/A	N/A	0	N/A	N/A	0	N/A	N/A	0	N/A	2	0
5-F-4-thio-ethyl-U	(44)	2	N/A	N/A	N/A	N/A	N/A	N/A	N/A	N/A	N/A	N/A	N/A	N/A	N/A	N/A	N/A	N/A	N/A	0	N/A	N/A	0	N/A	N/A	0	N/A	2	0
5-F-4-thio-benzyl-U	(45)	2	N/A	N/A	N/A	N/A	N/A	N/A	N/A	N/A	N/A	N/A	N/A	N/A	N/A	N/A	N/A	N/A	N/A	0	N/A	N/A	0	N/A	N/A	0	N/A	2	0
5-F-4-thio-phenyl-U	(46)	2	N/A	N/A	N/A	N/A	N/A	N/A	N/A	N/A	N/A	N/A	N/A	N/A	N/A	N/A	N/A	N/A	N/A	N/A	N/A	N/A	N/A	N/A	N/A	N/A	N/A	2	0
5-F-4-methoxy-U	(47)	2	N/A	N/A	N/A	N/A	N/A	N/A	N/A	N/A	N/A	N/A	N/A	N/A	N/A	N/A	N/A	N/A	N/A	0	N/A	N/A	2	N/A	N/A	0	N/A	2	0
5-F-4-butoxy-U	(48)	2	N/A	N/A	N/A	N/A	N/A	N/A	N/A	N/A	N/A	N/A	N/A	N/A	N/A	N/A	N/A	N/A	N/A	0	N/A	N/A	1	N/A	N/A	1	N/A	2	1
2′, 3′, 5′-tri-*O* -acetyl-5-F-4-butoxy-U	(49)	0	N/A	N/A	N/A	N/A	N/A	N/A	N/A	N/A	N/A	N/A	N/A	N/A	N/A	N/A	N/A	N/A	N/A	N/A	N/A	N/A	N/A	N/A	N/A	N/A	N/A	N/A	N/A
5-F-4-benzyloxy-U	(50)	2	N/A	N/A	N/A	N/A	N/A	N/A	N/A	N/A	N/A	N/A	N/A	N/A	N/A	N/A	N/A	N/A	N/A	0	N/A	N/A	1	N/A	N/A	1	N/A	2	1
N[4]-methylcytidine	(51)	2	2	2	2	2	2	2	N/A	N/A	N/A	N/A	N/A	N/A	N/A	N/A	N/A	N/A	N/A	2	0	2	2	2	2	2	2	2	2
N[4]-*N*[4]-dimethylcytidine	(52)	2	N/A	N/A	N/A	N/A	N/A	N/A	N/A	N/A	N/A	N/A	N/A	N/A	N/A	N/A	N/A	N/A	N/A	0	N/A	N/A	2	N/A	N/A	0	N/A	2	N/A
N[4]-2-hydroxyethyl-2'-dC	(53)	2	N/A	N/A	N/A	N/A	N/A	N/A	N/A	N/A	N/A	N/A	N/A	N/A	N/A	N/A	N/A	N/A	N/A	0	N/A	N/A	2	N/A	N/A	2	N/A	2	N/A
N[4]-aminoethyl-2'-dC	(54)	2	N/A	N/A	N/A	N/A	N/A	N/A	N/A	N/A	N/A	N/A	N/A	N/A	N/A	N/A	N/A	N/A	N/A	0	N/A	N/A	0	N/A	N/A	2	N/A	2	N/A
4-(4-morpholinyl)-2'-dU	(55)	2	2	0	0	2	0	0	N/A	N/A	N/A	N/A	N/A	N/A	N/A	N/A	N/A	N/A	N/A	0	0	0	2	0	0	0	N/A	2	0
5-F-4-(4-morpholinyl)-2'-dU	(56)	2	2	0	2	2	2	2	N/A	N/A	N/A	N/A	N/A	N/A	N/A	N/A	N/A	N/A	N/A	0	0	0	2	2	2	0	1	2	2
N[4]-hexyl-2'-dC	(57)	2	N/A	N/A	N/A	N/A	N/A	N/A	N/A	N/A	N/A	N/A	N/A	N/A	N/A	N/A	N/A	N/A	N/A	0	N/A	N/A	2	N/A	N/A	0	N/A	2	N/A
N[4]-(indolin-1-yl)-2'-dC	(58)	0	0	0	0	0	0	0	N/A	N/A	N/A	N/A	N/A	N/A	N/A	N/A	N/A	N/A	N/A	0	0	0	0	0	0	0	N/A	0	0
N[4]-(2,3,4,5,6-pentahydroxyhexyl)-2′-dC	(59)	2	0	0	0	0	0	0	N/A	N/A	N/A	N/A	N/A	N/A	N/A	N/A	N/A	N/A	N/A	0	0	0	0	0	0	0	N/A	2	0
N[4]-(1*H* -indol-6-yl)methyl)-2'-dC	(60)	2	2	0	2	0	2	2	N/A	N/A	N/A	N/A	N/A	N/A	N/A	N/A	N/A	N/A	N/A	0	0	0	0	0	0	0	1	2	2
Capecitabine	(61)	2	0	0	0	0	0	0	N/A	N/A	N/A	0	0	0	0	0	N/A	N/A	N/A	0	0	0	0	0	0	0	N/A	2	0
CMP	(62)	0	1	0	1	1	1	0	N/A	N/A	N/A	0	0	0	0	0	N/A	N/A	N/A	0	0	0	1	0	1	0	1	0	0

Figure S10. The specific activities of the CDAs mutants. The list of the tested substrates is shown on the left side. The number in brackets indicates the number of substrate (Fig. S7). The ID code representing each CDAs mutants is given at the top. The activity of enzymes is defined as follows: 2 – activity is observed after three hours of the incubation at RT; 1 – activity is observed after over-night incubation (weakly active), 0 – inactive towards the substrate, N/A – not analysed.

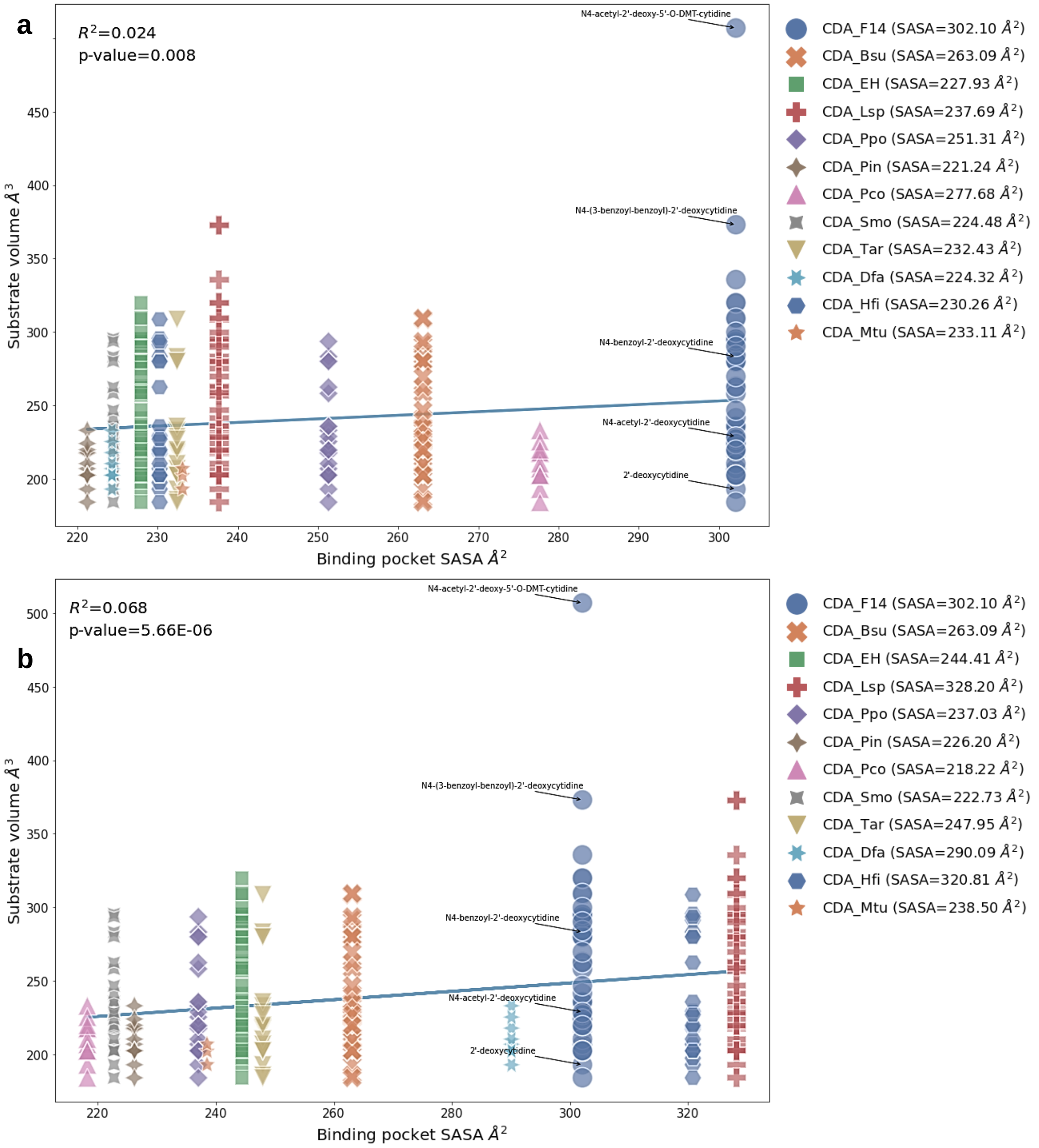

Figure S11. **The correlation of binding pocket of solvent accessible surface area (SASA) of CDA's with their substrate volumes. a** – Structures of CDA's were predicted by using AlphaFold2 (32), except CDA_F14 and CDA_Bsu, which PDB data were used; **b** – structures of CDA's were predicted on CDA_F14 structure template by using HHpred bioinformatics toolkit MODELLER (30).

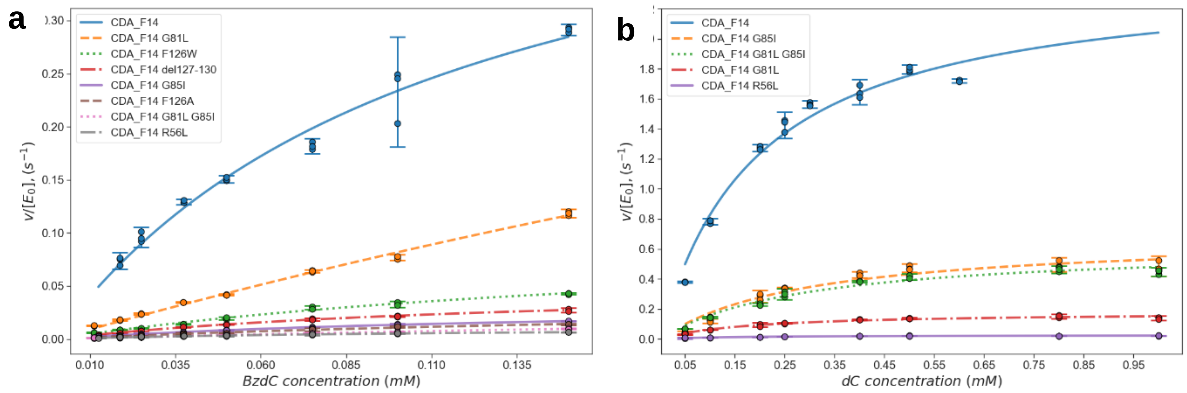

Figure S12. Activity graphs of CDA_F14 and its mutants. a – Catalytic efficiency of CDA_F14 and its mutants against BzdC (**25**), **b** – catalytic efficiency of CDA_F14 and its mutants against dC (**1**).

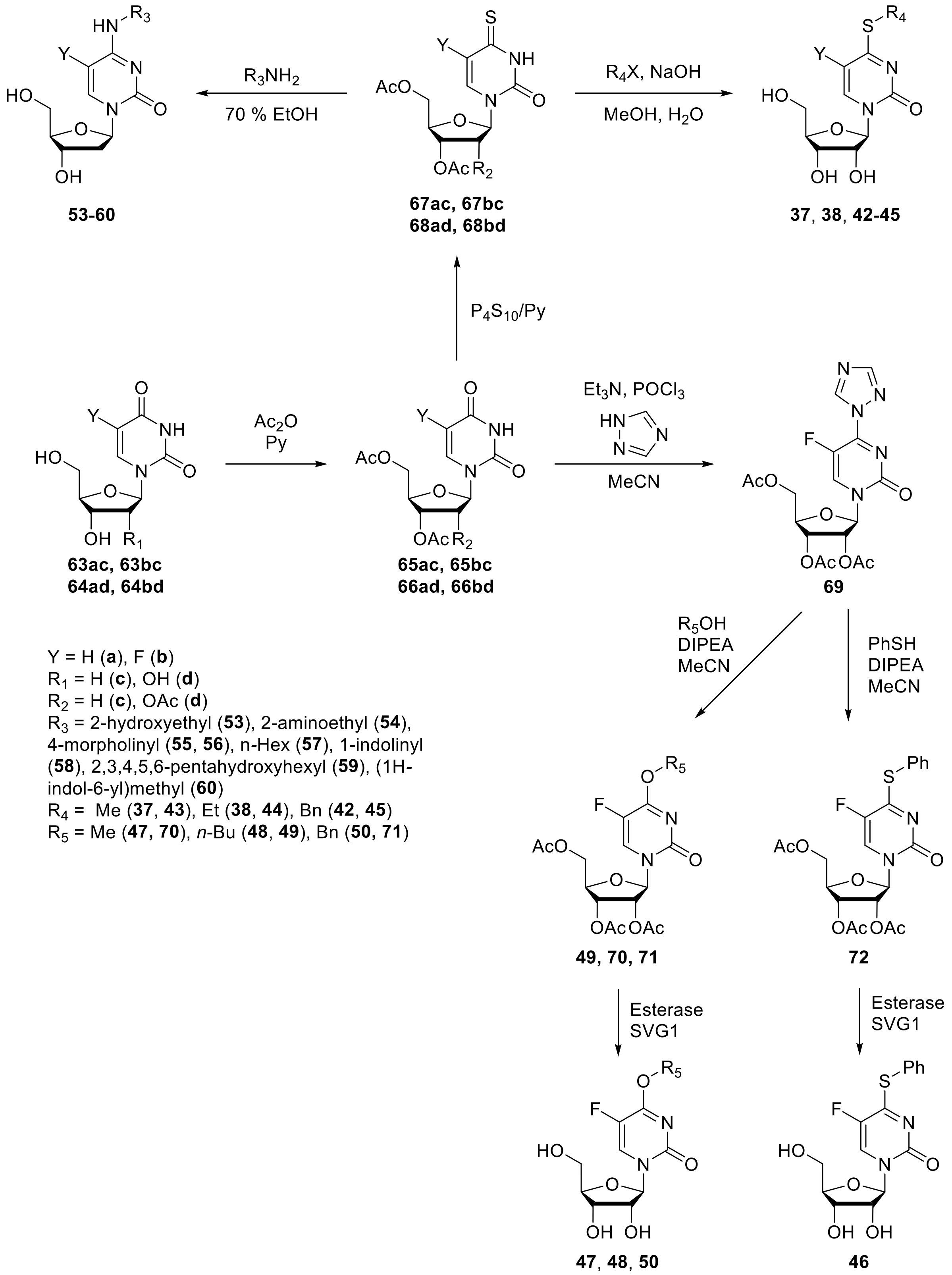

Figure S13. General scheme for the synthesis of *N*^{4}-alkylcytidine, *S*^{4}-alkylthiouridine and *O*^{4}-alkoxyuridine derivatives.

Table S1. The list of selected metagenomic cytidine deaminases and their nearest homologues. Asterisk * indicate CDA were selected on N^4-benzoyl-2'-deoxycytidine. M, E and V alphabet symbols indicate enzymes selected by using thiouridines substrates. **CDA encoded genes were synthesised, #CDA encoded genes were cloned from appropriate bacteria strain. CDA_Hsa- commercial enzyme.

No.	Cytidine deaminase, GenBank accession No.		Protein name, the nearest homologue genus, species, GenBank accession No.	Identity, %
**1**	CDA_F14*	OM350122	Cytidine deaminase [*Firmicutes* bacterium GWF2_51_9], OGS53896.1	85
**2**	CDA_EH*	OM350123	Cytidine deaminase [*Solobacterium* sp.], MBR2595354.1	99
**3**	CDA_F18*	OM350124	Cytidine deaminase [*Acidobacteria* bacterium 13_1_40CM_56_16], OLC32751.1	69
**4**	CDA_M1	OM350125	Cytidine deaminase [bacterium], MBI9032261.1	63
**5**	CDA_M2	OM350126	Cytidine deaminase [*Bacteroidales* bacterium], MZP55215.1	65
**6**	CDA_M13	OM350127	Cytidine deaminase [*Stomatobaculum* sp.], MBO6106869.1	86
**7**	CDA_E5	OM350128	Cytidine deaminase [*Bacteroidales* bacterium], MBE0674975.1	61
**8**	CDA_E8	OM350129	Cytidine deaminase [*Lachnospiraceae* bacterium], MBQ1931059.1	99
**9**	CDA_E9	OM350130	Cytidine deaminase [*Bacteroidales* bacterium], MBO6027659.1	95
**10**	CDA_E10	OM350131	Cytidine deaminase [*Choanephora cucurbitarum*], OBZ83712.1	49
**11**	CDA_V2	OM350132	Cytidine deaminase [*Bacteroidales* bacterium], MBR6546710.1	100
**12**	CDA_V4	OM350133	Cytidine deaminase [*Erysipelotrichaceae* bacterium], MBS6373486.1	71
**13**	CDA_V7	OM350134	Cytidine deaminase [*Anaerolinea* sp.], MBA4421068.1	66
**14**	CDA_V20	OM350135	Cytidine deaminase [*Lachnospiraceae* bacterium], MBR3186664.1	53
**15**	CDA_V30	OM350136	Cytidine deaminase [*Bacteroidales* bacterium], MBE6316241.1	100
**16**	CDA_V47	OM350137	Cytidine deaminase [*Clostridia* bacterium], MBR0206936.1	95
**17**	CDA_V52	OM350138	Cytidine deaminase [*Anaerolineales* bacterium], MBL8063484.1	79
**18**	CDA_V66	OM350139	Cytidine deaminase [*Acidobacteria* bacterium], MCA1579705.1	75
**19**	CDA_V75	OM350140	Cytidine deaminase [*Labilithrix* sp.], MBX3230353.1	85
**20**	CDA_V82	OM350141	Cytidine deaminase [*Schwartzia* sp.], MBO5514854.1	96
**21**	CDA_V86	OM350142	Cytidine deaminase [*Lachnospiraceae* bacterium] HCA22187.1	55
**22**	CDA_V94	OM350143	Cytidine deaminase [*Solobacterium* sp.], MBQ7992204.1	90

**23**	CDA_V106	OM350144	Cytidine deaminase [*Paenibacillus*], WP_009590810.1	100
**24**	CDA_V107	OM350145	Cytidine deaminase [*Paenibacillus* lautus], WP_213645365.1	99
**25**	CDA_V114	OM350146	Cytidine deaminase [*Bacillus stratosphericus* LAMA 585], EMI12311.1	99
**26**	CDA_V116	OM350147	MULTISPECIES: Cytidine deaminase [*Serratia*], WP_017893700.1	100
**27**	CDA_V123	OM350148	cytidine deaminase [*Bacteroidales* bacterium], MBR2225285.1	94
**28**	CDA_V125	OM350149	cytidine deaminase [*Bacteroidales* bacterium], MBR5300056.1	88
**29**	CDA_V135	OM350150	Cytidine deaminase [*Kofleriaceae* bacterium], NVB81525.1	66
**30**	CDA_V157	OM350151	Cytidine deaminase [*Cellulosilyticum* sp.], MBQ1275264.1	81
**31**	CDA_Hfi**	WP_117892443.1	Cytidine deaminase [*Holdemania filiformis*]	100
**32**	CDA_Lsp**	WP_204248541.1	Cytidine deaminase [*Lachnoclostridium* sp. An14]	100
**33**	CDA_Dfa**	HAH94059.1	Cytidine deaminase [*Dielma fastidiosa*]	100
**34**	CDA_Ppo**	WP_013373176.1	Cytidine deaminase [*Paenibacillus polymyxa*]	100
**35**	CDA_Mtu**	WP_003417264.1	Cytidine deaminase [*Mycobacterium tuberculosis*]	100
**36**	CDA_Pco**	WP_006849539.1	Cytidine deaminase [*Prevotella copri*]	100
**37**	CDA_Pin**	WP_044048061.1	Cytidine deaminase [*Prevotella intermedia*]	100
**38**	CDA_Smo**	WP_028077262.1	Cytidine deaminase [*Solobacterium moorei*]	100
**39**	CDA_Tar**	PBO85953.1	Cytidine deaminase [*Thaumarchaeota archaeon*]	100
**40**	CDA_Bsu[#]	WP_187956931.1	Cytidine deaminase [*Bacillus subtilis*]	100
**41**	CDA_Hsa	NP_001776.1	Cytidine deaminase [*Homo sapiens*]	100
**42**	CDA_Eco[#]	WP_112912831.1	Cytidine deaminase [*Escherichia coli*]	100

Table S2. Metagenomic libraries used in this work. Only the libraries that contained the positive hits are shown. Soil samples were taken from Vilnius region, Lithuania (54°40’N25°16’E).

**Metagenomic library**	**DNA source**	**Vector/ insertion site**	**Approx. number of clones in library**	**Number of selected hits (names of hits)**
F14-F17	Soil	pUC19/BamHI	77500	1 (CDA_F14)
F18-F19	Soil	pUC19/BamHI	180000	1 (CDA_F18)
EH	Cow dung	pUC19/BamHI	50000	1 (CDA_ EH)
Dus3	Lake water with sediments	pUC19/*Hind*III	175000	2 (CDA_M1, CDA_M2)
Mix of libraries: ZVK, ISER, DS1, RA	Lake water with sediments	pUC19/*Hind*III	1200000	1 (CDA_E5)
Mix of libraries: RI8, RI26	Soil	R18:pUC19/PstI, RI26:pUC19/BamHI	2200000	4 (CDA_E8, CDA_E9, CDA_E10, CDA_M13)
RI23	Soil	pUC19/BamHI	3500000	3 (CDA_V2, CDA_V4, CDA_V7)
LINK	Pond water with sediments	pUC19/*Hind*III	29000	2 (CDA_V20, CDA_V21)
Mix of libraries: 17, 47, 55	Soil	pUC19//PstI	1000000	1 (CDA_V30)
RI14	Soil	pUC19//PstI	500000	1 (CDA_V47)
LINKBam	Pond water with sediments	pUC19/BamHI	23000	1 (CDA_V52)
Mix of libraries: RECN, ReJU10	Soil	pUC19//PstI	90000	1 (CDA_V66)
Mix of libraries: 201, 202, 203, 204, 205, 206, 207, 208	Soil	pUC19/BamHI pUC19/*Hind*III pUC19//PstI	1200000	1 (CDA_V75)
Mix of libraries: 21, 137, 139, 151, 152	Soil	pUC19/*Hind*III	175000	2 (CDA_V82, CDA_V86)
MSL	Cow dung	pUC19/BamHI	50000	1 (CDA_V94)
Mix of libraries: MO2, MO4, MO12, MO14	Soil	pUC19//PstI	700000	2 (CDA_V106, CDA_V107)
RGH	Soil	pUC19/*Hind*III	350000	1 (CDA_V114)
Mix of libraries: MO2, MO4, MO12, MO14, 14, 17, 55, 133-183, CiBam, ZGBam, MO9, MO10, MO15, 185-193, RI26, RI9	Soil	pUC19/BamHI pUC19/*Hind*III pUC19//PstI	5000000	3 (CDA_V116, CDA_V123, CDA_V125)
LINK10	Pond water with sediments	pUC19/*Hind*III	370000	1 (CDA_V135)
AL25	Alpaca faeces/dung	pUC19/*Hind*III	140000	1 (CDA_V157)

Table S3. Data collection and refinement statistics.

**Data Collection**	
Wavelength	0.9763
Resolution range, Å	48.63 - 1.2 (1.243 - 1.2)
Space group	C 1 2 1
Unit cell	97.484 128.899 97.259<br>90 90.016 90
Total / unique reflections	2430245 (243928)/ 371943 (36936)
Multiplicity	6.5 (6.6)
Completeness (%)	99.71 (99.16)
Mean I/sigma(I)	19.34 (7.44)
Wilson B-factor	12.97
R-merge[a]	0.06849 (0.3537)
CC1/2[b]	0.994 (0.97)
**Refinement**	
Reflections used in refinement (total / R-free)	371870 / 36867
R-work[c]/ R-free[d], %	14.43 / 16.03
Number of non-hydrogen atoms (macromolecules / ligands / solvent)	8793 / 146 /714
RMS (bonds / angles)	0.008 / 0.99
Ramachandran: favoured / allowed / outliers (%)	99.81 / 0.19 /0.00
Rotamer outliers (%)	0.22
Clashscore	0.68
Average B-factor (protein / ligand / solvent)	18.15 (17.57 / 13.68 / 26.33)

Data collection statistics for the highest-resolution shell are shown in parentheses.

[a] $R_{merge} = \Sigma_{hkl} \sum_i |I_i(hkl) - \langle I(hkl)\rangle| / \sum_{hkl} \sum_i I_i(hkl)$, where $I_i(hkl)$ is the intensity of the measured reflection (hkl) and n denotes multiplicity.

[b] $CC_{1/2}$ is the correlation coefficient of the half datasets.

[c] R-work = $\Sigma ||F_{obs}| - |F_{calc}|| / \Sigma |F_{obs}|$, where F_{obs} and F_{calc} are observed and calculated structure factors.

[d] R-free = $\Sigma T||F_{obs}| - |F_{calc}|| / \Sigma T|F_{obs}|$, where T is a test dataset of ∼10% of the total reflections randomly chosen and set aside prior to refinement.

Table S4. Possible hydrogen bonds in CDA_F14 structure. Possible hydrogen bonds in CDA_F14 structure between BzdC and the enzyme**.** Table shows hydrogen bonds that formed during a 100 ns molecular dynamics simulation. The structures were snapshotted every 0.5 ns for a total of 200 frames. Acceptor - hydrogen bond acceptor atoms, DonorH – donor hydrogen atoms, Donor – donor atoms binding hydrogen, Frames – number of frames, out of a total of 200, when the bond was formed, AvgDist – average hydrogen bond distance over the simulation, AvgAng – average hydrogen bond angle over the simulation.

**#Acceptor**	**DonorH**	**Donor**	**Frames**	**Frac**	**AvgDist**	**AvgAng**
GLU_55@OE2	UNL_541@H18	UNL_541@O2	198	0.99	2.6195	166.4992
GLU_44@OE2	UNL_541@H14	UNL_541@O3'	194	0.97	2.6914	168.4034
GLU_55@OE1	UNL_541@H2	UNL_541@N1	188	0.94	2.791	151.9481
ALA_448@O	UNL_541@H17	UNL_541@O5'	170	0.85	2.7262	157.3839
GLU_55@OE2	UNL_541@H3	UNL_541@N2	163	0.815	2.7818	143.3205
UNL_541@O3'	ASN_42@HD22	ASN_42@ND2	122	0.61	2.8817	156.7407
UNL_541@O1	TYR_24@HH	TYR_24@OH	98	0.49	2.7555	163.0316
UNL_541@O	GLY_54@H	GLY_54@N	55	0.275	2.8868	159.0783
UNL_541@O5'	TYR_450@H	TYR_450@N	44	0.22	2.9243	166.8029
GLU_55@OE1	UNL_541@H18	UNL_541@O2	16	0.08	2.848	141.7439

Table S5. Kinetic parameters of CDAs. Substrate: N^4-benzoyl-2'-deoxycytidine (**25**); 2'-deoxycytidine (**1**).

**CDA**	**Substrate**	**K_M, (M)**	**k_{cat}, (s^{-1})**	**k_{cat}/K_M (M^{-1} S^{-1})**
**F14 (wt)**	(**25**)	$(1.15 \pm 0.16) \times 10^{-4}$	$(5.04 \pm 0.4) \times 10^{-1}$	$(4.36 \pm 3.61) \times 10^{3}$
	(**1**)	$(1.95 \pm 0.36) \times 10^{-4}$	$(24.4 \pm 1.71) \times 10^{-1}$	$(1.25 \pm 0.12) \times 10^{4}$
**F14_del127-130**	(**25**)	$(1.46 \pm 0.01) \times 10^{-4}$	$(5.41 \pm 0.23) \times 10^{-2}$	$(3.70 \pm 0.09) \times 10^{2}$
**F14_del83-85**	(**25**)	not determined	not determined	
	(**1**)	not determined	not determined	
**F14_F126A**	(**25**)	$(2.59 \pm 0.73) \times 10^{-4}$	$(3.88 \pm 0.8) \times 10^{-2}$	$(1.50 \pm 0.09) \times 10^{2}$
**F14_F126W**	(**25**)	$(2.11 \pm 0.38) \times 10^{-4}$	$(10.4 \pm 1.3) \times 10^{-2}$	$(4.92 \pm 0.24) \times 10^{2}$
**F14_G85I**	(**25**)	$(1.56 \pm 0.1) \times 10^{-4}$	$(3.44 \pm 0.14) \times 10^{-2}$	$(2.2 \pm 0.05) \times 10^{2}$
	(**1**)	$(2.96 \pm 0.9) \times 10^{-4}$	$(6.9 \pm 0.8) \times 10^{-1}$	$(2.32 \pm 0.3) \times 10^{3}$
**F14_G81L**	(**25**)	$(8.66 \pm 3.8) \times 10^{-4}$	$(7.89 \pm 3.08) \times 10^{-1}$	$(9.11 \pm 0.3) \times 10^{2}$
	(**1**)	$(2.27 \pm 0.15) \times 10^{-4}$	$(1.79 \pm 0.09) \times 10^{-1}$	$(9.70 \pm 0.9) \times 10^{2}$
**F14_G81LG85I**	(**25**)	$(4.93 \pm 1.3) \times 10^{-4}$	$(4.14 \pm 0.88) \times 10^{-2}$	$(8.40 \pm 0.3) \times 10^{1}$
	(**1**)	$(2.93 \pm 0.5) \times 10^{-4}$	$(6.2 \pm 0.44) \times 10^{-1}$	$(2.12 \pm 0.2) \times 10^{3}$
**F14_R56L**	(**25**)	$(1.33 \pm 0.17) \times 10^{-4}$	$(1.2 \pm 0.09) \times 10^{-2}$	$(9.20 \pm 0.42) \times 10^{1}$
	(**1**)	$(1.67 \pm 0.19) \times 10^{-4}$	$(2.58 \pm 0.09) \times 10^{-2}$	$(1.55 \pm 0.11) \times 10^{2}$

Table S6. Primers used for the amplification of the genes of the selected cytidine deaminases. The 5' end of the primer (highlighted in the underlined italic font) includes the pLATE31 vector-specific sequences for the ligation-independent cloning.

**Clone**	**Forward primer (5'->3')**	**Reverse primer (5'->3')**
F14	*AGAAGGAGATATAACTATG*ATGAACAAGGAAGATTTATTG	*GTGGTGGTGATGGTGATGGCC*TAAAAGATCCTCTTTCGTGA
F18	*AGAAGGAGATATAACTATGAGGC*ATGACCTCGTC	*GTGGTGGTGATGGTGATGGCC*ACCCAAACTGTTCGCGTC
EH	*AGAAGGAGATATAACT*ATGAAGGAAACACTTTGTCA	*GTGGTGGTGATGGTGATGGCC*TTTTCCGTAATCCTCGCTC
Eco	*AGAAGGAGATATAACT*ATGCATCCACGTTTTCAAAC	*GTGGTGGTGATGGTGATGGCC*AGCGAGAAGCACTCGG
Bsu	*AGAAGGAGATATAACT*ATGAACAGACAAGAATTAATAAC	*GTGGTGGTGATGGTGATGGCC*AAGCTTTCGTTCGTCATGTAA
E10	*AGAAGGAGATATAACT*ATGCGGACCAAAGAAGATT	*GTGGTGGTGATGGTGATGGCC*GTCGAGCAGGCGGAAAG
E5	*AGAAGGAGATATAACT*ATGAAAAAGATATCAATTACAAC	*GTGGTGGTGATGGTGATGGCC*CTCATCCGAAGAGCTAAG
E8	*AGAAGGAGATATAACT*ATGAGTAAGGTTTCTGATCAGG	*GTGGTGGTGATGGTGATGGCC*TGCCAGATCCTTCGGTC
E9	*AGAAGGAGATATAACT*ATGACCGACAGACGCATCA	*GTGGTGGTGATGGTGATGGCC*TTCCACTTTCAAACTGTCGA
M1	*AGAAGGAGATATAACT*ATGATATTATTGAGCGAAGCT	*GTGGTGGTGATGGTGATGGCC*CTCATCAGTTAAAGTAAATCG
M2	*AGAAGGAGATATAACT*ATGGTAACTAAGAATATTTCC	*GTGGTGGTGATGGTGATGGCC*CAGAGGCTTGATCTTCAG
M13	*AGAAGGAGATATAACT*ATGAGCGCGGACGAAAG	*GTGGTGGTGATGGTGATGGCC*TTTATCCAGATCTCCCGG
V4	*AGAAGGAGATATAACT*ATGACAGATTGGACATATTTGAT	*GTGGTGGTGATGGTGATGGCC*TTTTGCAAGATCTTCTGC
V20	*AGAAGGAGATATAACT*ATGGACCTCGAGCGC	*GTGGTGGTGATGGTGATGGCC*CTTCTTCGCCATATAGTTC
V30	*AGAAGGAGATATAACT*ATGACCGAAAAGAAAATCACC	*GTGGTGGTGATGGTGATGGCC*AAACTCGGTGAAGGAAAG
V47	*AGAAGGAGATATAACT*ATGAACCAACTGACGCAAC	*GTGGTGGTGATGGTGATGGCC*GACCCCCAGGTCCTTGG
V52	*AGAAGGAGATATAACT*ATGACCAATGTCACTCCCG	GTGGTGGTGATGGTGATGGCCGGATTTTAGATGCGCGGG
V75	*AGAAGGAGATATAACT*ATGAGCAGCAAGAAGAAGG	*GTGGTGGTGATGGTGATGGCC*GTGTTTCAGGCGGAACTG
V82	*AGAAGGAGATATAACT*ATGACAGAGACAGACAAGAAG	*GTGGTGGTGATGGTGATGGCC*TCCGTTAATGTCCTCGGC
V86	*AGAAGGAGATATAACT*ATGAATAAAGAAGTGATATCTGA	*GTGGTGGTGATGGTGATGGCC*ATTCTCAAGTGCTTCAGC
V106	*AGAAGGAGATATAACT*ATGGATGCAGGTTTGTTGATG	*GTGGTGGTGATGGTGATGGCC*CTGCTTGTTTAAATCCCATG

Table S7. Primers used for the site-directed mutagenesis.

Mutant	Forward primer (5'->3')	Reverse primer (5'->3')
F14_T51G	TTACGGAGCGGGTAATTGCGG	GAAGCATTTTCGATATTTGCGCCTAAAAAC
F14_G85L	TGATAGGGTCATCGCACCTTGCG	CCATCGGTTACGATCGCCAACG
F14G81I	CGATCGTAACCGATTTGGATAGGGTC	CCAACGCTTCGATGTCGTCAG
F14_R56L	ATTGCGGTGAATTAAGTGCCATTTTCGC	TGGTCGCTCCGTAAGAAGCATTTTCG
F14_F126A	GATGCGAGCAACGAAAGAGGATCTTTTAG	GGCAACAATTCATCGATCGTTTTTTCTAACG
F14_F126W	GATGCGATGGACGAAAGAGGATCTTTTAG	GGCAACAATTCATCGATCGTTTTTTCTAACG
F14_del83-85	GCACCTTGCGGAATCTGCC	ATCACCATCGGTTACGATCGCC
F14_mutdel83-85	NNNNNNNNNGCACCTTGCGGAATCTGCC	ATCACCATCGGTTACGATCGCC
F14_del127-130	CTTTTAGGCCATCACCATCACCAC	GAATCGCATCGGCAACAATTCATC
F14_mutdel127-130	NNNNNNNNNNNNCTTTTAGGCCATCACCATCACCAC	GAATCGCATCGGCAACAATTCATC
Pco_G70T	TCCCGTCTACCCTGTGCGCG	ACGCCGCGTTTTCCTGGTTCG
Pco_I108A	TGAAATCTCCGGCGTCTCCGTGC	GGAAACCGTTACCGTTACGCGCC
Ppo_C50T	CGGTCTGACCAACTGCGCGG	TAAGACGCGTTTTCAACGTTGCAACCG
Ppo_V82L	GAAGGTCCGCTGTCTCCGTGC	GGTGTCCGCCGCAACCG
Tar_I85A	AAAAACCGGCGTTCCCGTGCG	CCGGCGCGATAGAAGAAGAGATCGC
Lsp_A82I	TAACGCGGACATCGCGCCGTG	CCGTTGCAAACAACCGCCAGCG
R56L	ATTGCGGTGAATTAAGTGCCATTTTCGC	TGGTCGCTCCGTAAGAAGCATTTTCG
F14_C53H_R56Q	TCCGTAAGAAGCATTTTCGATATTTGCGCC	GCGACCAATCATGGTGAACAAAGTGCC
F14_C88H_C91H	ACCGACCCTATCACCATCGGTTAC	GCACCTCACGGAATCCACCGTCAAG

Data S1. Spreadsheet of specific activity of CDA_F14 (for fig.2)
Data S2. Set of CDAs homologous sequences (identity>50) (for fig.3).
Data S3. Set of representative CDA homologs used for plotting (for fig.3).

REFERENCES AND NOTES

1. P. Boccaletto, F. Stefaniak, A. Ray, A. Cappannini, S. Mukherjee, E. Purta, M. Kurkowska, N. Shirvanizadeh, E. Destefanis, P. Groza, G. Avşar, A. Romitelli, P. Pir, E. Dassi, S. G. Conticello, F. Aguilo, J. M. Bujnicki, MODOMICS: A database of RNA modification pathways. 2021 update. *Nucleic Acids Res.* **50**, D231–D235 (2022).

2. A. J. Sood, C. Viner, M. M. Hoffman, DNAmod: The DNA modification database. *J. Cheminform.* **11**, 30 (2019).

3. P. J. McCown, A. Ruszkowska, C. N. Kunkler, K. Breger, J. P. Hulewicz, M. C. Wang, N. A. Springer, J. A. Brown, Naturally occurring modified *ribonucleosides Wiley Interdiscip. Rev. RNA* **11**, e1595 (2020).

4. S. Hong, X. Cheng, DNA base flipping: A general mechanism for writing, reading, and erasing DNA modifications. *Adv. Exp. Med. Biol.* **945**, 321–341 (2016).

5. W. L. Nyhan, Nucleotide Synthesis Via Salvage Pathway, in *eLS* (John Wiley & Sons Ltd, 2014).

6. L. M. Iyer, D. Zhang, I. B. Rogozin, L. Aravind, Evolution of the deaminase fold and multiple origins of eukaryotic editing and mutagenic nucleic acid deaminases from bacterial toxin systems. *Nucleic Acids Res.* **39**, 9473–9497 (2011).

7. G. Niu, H. Tan, Nucleoside antibiotics: Biosynthesis, regulation, and biotechnology. *Trends Microbiol.* **23**, 110–119 (2015).

8. L. Li, J. Wu, Z. Deng, T. M. Zabriskie, X. He, Streptomyces lividans blasticidin S deaminase and its application in engineering a blasticidin S-producing strain for ease of genetic manipulation. *Appl. Environ. Microbiol.* **79**, 2349–2357 (2013).

9. N. Navaratnam, R. Sarwar, An overview of cytidine deaminases. *Int. J. Hematol.* **83**, 195–200 (2006).

10. A. R. Ramiro, V. M. Barreto, Activation-induced cytidine deaminase and active cytidine demethylation. *Trends Biochem. Sci.* **40**, 172–181 (2015).

11. L. Betts, S. Xiang, S. A. Short, R. Wolfenden, C. W. Carter Jr., Cytidine deaminase. The 2.3 A crystal structure of an enzyme: Transition-state analog complex. *J. Mol. Biol.* **235**, 635–656 (1994).

12. S. E. Faivre-Nitschke, J. M. Grienenberger, J. M. Gualberto, A prokaryotic-type cytidine deaminase from *Arabidopsis thaliana* gene expression and functional characterization. *Eur. J. Biochem.* **263**, 896–903 (1999).

13. W. Liu, F. Shang, Y. Chen, J. Lan, L. Wang, J. Chen, P. Gao, N.-C. Ha, C. Quan, K. H. Nam, Y. Xu, Biochemical and structural analysis of the *Klebsiella pneumoniae* cytidine deaminase CDA. *Biochem. Biophys. Res. Commun.* **519**, 280–286 (2019).

14. S. Costanzi, S. Vincenzetti, G. Cristalli, A. Vita, Human cytidine deaminase: A three-dimensional homology model of a tetrameric metallo-enzyme inferred from the crystal structure of a distantly related dimeric homologue. *J. Mol. Graph. Model.* **25**, 10–16 (2006).

15. E. Johansson, N. Mejlhede, J. Neuhard, S. Larsen, Crystal structure of the tetrameric cytidine deaminase from *Bacillus subtilis* at 2.0 Å resolution. *Biochemistry* **41**, 2563–2570 (2002).

16. C. Serdjebi, G. Milano, J. Ciccolini, Role of cytidine deaminase in toxicity and efficacy of nucleosidic analogs. *Expert Opin. Drug Metab. Toxicol.* **11**, 665–672 (2015).

17. C. M. Walko, C. Lindley, Capecitabine: A review. *Clin. Ther.* **27**, 23–44 (2005).

18. R. M. Cohen, R. Wolfenden, Cytidine deaminase from *Escherichia coli*. *J. Biol. Chem.* **246**, 7561–7565 (1971).

19. A. J. Burke, W. R. Birmingham, Y. Zhuo, T. W. Thorpe, B. Zucoloto da Costa, R. Crawshaw, I. Rowles, J. D. Finnigan, C. Young, G. M. Holgate, M. P. Muldowney, S. J. Charnock, S. L. Lovelock, N. J. Turner, A. P. Green, An engineered cytidine deaminase for biocatalytic production of a key intermediate of the Covid-19 antiviral molnupiravir. *J. Am. Chem. Soc.* **144**, 3761–3765 (2022).

20. A. M. Goble, H. Fan, A. Sali, F. M. Raushel, Discovery of a cytokinin deaminase. *ACS Chem. Biol.* **6**, 1036–1040 (2011).

21. N. Urbelienė, R. Meškienė, M. Tiškus, R. Stanislauskienė, A. Aučynaitė, A. Laurynėnas, R. Meškys, A rapid method for the selection of amidohydrolases from metagenomic libraries by applying synthetic nucleosides and a uridine auxotrophic host. *Catalysts* **10**, 445 (2020).

22. A. Frances, P. Cordelier, The emerging role of cytidine deaminase in human diseases: A new opportunity for therapy? *Mol. Ther.* **28**, 357–366 (2020).

23. A. Aučynaitė, R. Rutkienė, R. Gasparavičiūtė, R. Meškys, J. Urbonavičius, A gene encoding a DUF523 domain protein is involved in the conversion of 2-thiouracil into uracil. *Environ. Microbiol. Rep.* **10**, 49–56 (2018).

24. K. Clark, I. Karsch-Mizrachi, D. J. Lipman, J. Ostell, E. W. Sayers, GenBank. *Nucleic Acids Res.* **44**, D67–72 (2016).

25. E. Johansson, J. Neuhard, M. Willemoës, S. Larsen, Structural, kinetic, and mutational studies of the zinc ion environment in tetrameric cytidine deaminase. *Biochemistry* **43**, 6020–6029 (2004).

26. B. E. Suzek, H. Huang, P. McGarvey, R. Mazumder, C. H. Wu, UniRef: Comprehensive and non-redundant UniProt reference clusters. *Bioinformatics* **23**, 1282–1288 (2007).

27. F. Pedregosa, G. Varoquaux, A. Gramfort, V. Michel, B. Thirion, O. Grisel, M. Blondel, P. Prettenhofer, R. Weiss, V. Dubourg, J. Vanderplas, A. Passos, D. Cournapeau, Scikit-learn: Machine learning in Python. *J. Mach. Learn. Res.* **12**, 2825–2830 (2011).

28. O. Trott, A. J. Olson, AutoDock Vina: Improving the speed and accuracy of docking with a new scoring function, efficient optimization and multithreading. *J. Comput. Chem.* **31**, 455–461 (2010).

29. T. Matsubara, M. Ishikura, M. Aida, A quantum chemical study of the catalysis for cytidine deaminase: Contribution of the extra water molecule. *J. Chem. Inf. Model.* **46**, 1276–1285 (2006).

30. J. Jumper, R. Evans, A. Pritzel, T. Green, M. Figurnov, O. Ronneberger, K. Tunyasuvunakool, R. Bates, A. Žídek, A. Potapenko, A. Bridgland, C. Meyer, S. A. A. Kohl, A. J. Ballard, A. Cowie, B. Romera-Paredes, S. Nikolov, R. Jain, J. Adler, T. Back, S. Petersen, D. Reiman, E. Clancy, M. Zielinski, M. Steinegger, M. Pacholska, T. Berghammer, S. Bodenstein, D. Silver, O. Vinyals, A. W. Senior, K. Kavukcuoglu, P. Kohli, D. Hassabis, Highly accurate protein structure prediction with AlphaFold. *Nature* **596**, 583–589 (2021).

31. L. Zimmermann, A. Stephens, S.Z. Nam, D. Rau, J. Kübler, M. Lozajic, F. Gabler, J. Söding, A. N. Lupas, V. Alva, A completely reimplemented MPI bioinformatics toolkit with a new HHpred server at its core. *J. Mol. Biol.* **430**, 2237–2243 (2018).

32. Z. A. Sánchez-Quitian, L. F.S.M. Timmers, R. A. Caceres, J. G. Rehm, C. E. Thompson, L. A. Basso, W. F. de Azevedo Jr, D. S. Santos, Crystal structure determination and dynamic studies of *Mycobacterium tuberculosis* cytidine deaminase in complex with products. *Arch. Biochem. Biophys.* **509**, 108–115 (2011).

33. N. Shigi, Biosynthesis and degradation of sulfur modifications in tRNAs. *Int. J. Mol. Sci.* **22**, 11937 (2021).

34. C. Borek, V. F. Reichle, S. Kellner, Synthesis and metabolic fate of 4-methylthiouridine in bacterial tRNA. *Chembiochem* **21**, 2768–2771 (2020).

35. R. Stanislauskienė, A. Laurynėnas, R. Rutkienė, A. Aučynaitė, D. Tauraitė, R. Meškienė, N. Urbelienė, A. Kaupinis, M. Valius, L. Kaliniene, R. Meškys, YqfB protein from *Escherichia coli*: An atypical amidohydrolase active towards N4-acylcytosine derivatives. *Sci. Rep.* **10**, (2020), 788.

36. N. Shrivastav, D. Li, J. M. Essigmann, Chemical biology of mutagenesis and DNA repair: Cellular responses to DNA alkylation. *Carcinogenesis* **31**, 59–70 (2010).

37. A. E. Pegg, Multifaceted roles of alkyltransferase and related proteins in DNA repair, DNA damage, resistance to chemotherapy and research tools. *Chem. Res. Toxicol.* **24**, 618–639 (2011).

38. M. Zimmermann, M. Zimmermann-Kogadeeva, R. Wegmann, A. L. Goodman, Mapping human microbiome drug metabolism by gut bacteria and their genes. *Nature* **570**, 462–467 (2019).

39. B. Javdan, J. G. Lopez, P. Chankhamjon, Y.C. J. Lee, R. Hull, Q. Wu, X. Wang, S. Chatterjee, M. S. Donia, Personalized mapping of drug metabolism by the human gut microbiome. *Cell* **181**, 1661–1679.e22 (2020).

40. J. Jakubovska, D. Tauraite, L. Birštonas, R. Meškys, N^4-acyl-2′-deoxycytidine-5′-triphosphates for the enzymatic synthesis of modified DNA. *Nucleic Acids Res.* **46**, 5911–5923 (2018).

41. N. Urbelienė, S. Kutanovas, R. Meškienė, R. Gasparavičiūtė, D. Tauraitė, M. Koplūnaitė, R. Meškys, Application of the uridine auxotrophic host and synthetic nucleosides for a rapid selection of hydrolases from metagenomic libraries. *J. Microbial. Biotechnol.* **12**, 148–160 (2019).

42. S. Kumar, G. Stecher, M. Li, C. Knyaz, K. Tamura, MEGA X: Molecular evolutionary genetics analysis across computing platforms. *Mol. Biol. Evol.* **35**, 1547–1549 (2018).

43. T. Baba, T. Ara, M. Hasegawa, Y. Takai, Y. Okumura, M. Baba, K.A. Datsenko, M. Tomita, B.L. Wanner, H. Mori, Construction of *Escherichia coli* K-12 in-frame, single-gene knockout mutants: The Keio collection. *Mol. Syst. Biol.* **2**, 2006.0008 (2006).

44. F. Sievers, D. G. Higgins, Clustal omega for making accurate alignments of many protein sequences. *Protein Sci. Publ. Protein Soc.* **27**, 135–145 (2018).

45. M. Remmert, A. Biegert, A. Hauser, J. Söding, HHblits: Lightning-fast iterative protein sequence searching by HMM-HMM alignment. *Nat. Methods* **9**, 173–175 (2012).

46. M. Steinegger, J. Söding, MMseqs2 enables sensitive protein sequence searching for the analysis of massive data sets. *Nat. Biotechnol.* **35**, 1026–1028 (2017).

47. W. Kabsch, XDS. *Acta Crystallogr. D Biol. Crystallogr.* **66**, 125–132 (2010).

48. The CCP4 suite: Programs for protein crystallography. *Acta Crystallogr. D Biol. Crystallogr.* **50**, 760–763 (1994).

49. A. Waterhouse, M. Bertoni, S. Bienert, G. Studer, G. Tauriello, R. Gumienny, F. T. Heer, T. A. P. de Beer, C. Rempfer, L. Bordoli, R. Lepore, T. Schwede, SWISS-MODEL: Homology modelling of protein structures and complexes. *Nucleic Acids Res.* **46**, W296–W303 (2018).

50. A. J. McCoy, R. W. Grosse-Kunstleve, P. D. Adams, M. D. Winn, L. C. Storoni, R. J. Read, Phaser crystallographic software. *J. Appl. Cryst.* **40**, 658–674 (2007).

51. P. Emsley, K. Cowtan, Coot: Model-building tools for molecular graphics. *Acta Crystallogr. D Biol. Crystallogr.* **60**, 2126–2132 (2004).

52. P. V. Afonine, R. W. Grosse-Kunstleve, N. Echols, J. J. Headd, N. W. Moriarty, M. Mustyakimov, T. C. Terwilliger, A. Urzhumtsev, P. H. Zwart, P. D. Adams, Towards automated crystallographic structure refinement with phenix.refine. *Acta Crystallogr. D Biol. Crystallogr.* **68**, 352–367 (2012).

53. E. F. Pettersen, T. D. Goddard, C. C. Huang, G. S. Couch, D. M. Greenblatt, E. C. Meng, T. E. Ferrin, UCSF Chimera-a visualization system for exploratory research and analysis. *J. Comput. Chem.* **25**, 1605–1612 (2004).

54. F. Gabler, S.-Z. Nam, S. Till, M. Mirdita, M. Steinegger, J. Söding, A. N. Lupas, V. Alva, Protein sequence analysis using the MPI bioinformatics toolkit. *Curr. Protoc. Bioinforma.* **72**, e108 (2020).

55. M. Baek, F. DiMaio, I. Anishchenko, J. Dauparas, S. Ovchinnikov, G. R. Lee, J. Wang, Q. Cong, L. N. Kinch, R. D. Schaeffer, C. Millán, H. Park, C. Adams, C. R. Glassman, A. DeGiovanni, J. H. Pereira, A. V. Rodrigues, A. A. van Dijk, A. C. Ebrecht, D. J. Opperman, T. Sagmeister, C. Buhlheller, T. Pavkov-Keller, M. K. Rathinaswamy, U. Dalwadi, C. K. Yip, J. E. Burke, K. C. Garcia, N. V. Grishin, P. D. Adams, R. J. Read, D. Baker, Accurate prediction of

protein structures and interactions using a three-track neural network. *Science* **373**, 871–876 (2021).

56. N. Hiranuma, H. Park, M. Baek, I. Anishchenko, J. Dauparas, D. Baker, Improved protein structure refinement guided by deep learning based accuracy estimation. *Nat. Commun.* **12**, 1340 (2021).

57. J. Dapkūnas, K. Olechnovič, Č. Venclovas, Modeling of protein complexes in CAPRI Round 37 using template-based approach combined with model selection. *Proteins* **86** (Suppl. 1), 292–301 (2018).

58. K. Olechnovič, Č. Venclovas, VoroMQA: Assessment of protein structure quality using interatomic contact areas. *Proteins* **85**, 1131–1145 (2017).

59. J. D. Hunter, Matplotlib: A 2D graphics environment. *Comput. Sci. Eng.* **9**, 90–95 (2007).

60. A. Shrake, J. A. Rupley, Environment and exposure to solvent of protein atoms. Lysozyme and insulin. *J. Mol. Biol.* **79**, 351–371 (1973).

61. P. J. A. Cock, T. Antao, J. T. Chang, B. A. Chapman, C. J. Cox, A. Dalke, I. Friedberg, T. Hamelryck, F. Kauff, B. Wilczynski, M. J. L. de Hoon, Biopython: Freely available Python tools for computational molecular biology and bioinformatics. *Bioinformatics* **25**, 1422–1423 (2009).

62. R. A. Laskowski, M. B. Swindells, LigPlot+: Multiple ligand-protein interaction diagrams for drug discovery. *J. Chem. Inf. Model.* **51**, 2778–2786 (2011).

63. Y. Gong, L. Chen, W. Zhang, R. Salter, Transglycosylation in the modification and isotope labeling of pyrimidine nucleosides. *Org. Lett.* **22**, 5577–5581 (2020).

64. J. Milecki, J. Nowak, B. Skalski, S. Franzen, 5-Fluoro-4-thiouridine phosphoramidite: New synthon for introducing photoaffinity label into oligodeoxynucleotides. *Bioorg. Med. Chem.* **19**, 6098–6106 (2011).

65. Z. Kaleta, B. T. Makowski, T. Soós, R. Dembinski, Thionation using fluorous Lawesson's reagent. *Org. Lett.* **8**, 1625–1628 (2006).

66. G. Wenska, K. Taras-Goslinska, P. Filipiak, G. L. Hug, B. Marciniak, Photochemical reactions of 4-thiouridine disulfide and 4-benzylthiouridine—The involvement of the 4-pyrimidinylthiyl radical. *Photochem. Photobiol. Sci.* **7**, 250–256 (2008).

67. A. Kraszewski, A. M. Delort, R. Teoule, Synthesis of 4-mono- and dialkyl-2′-deoxycytidines and their insertion into an oligonucleotide. *Tetrahedron Lett.* **27**, 861–864 (1986).

68. X. Robert, P. Gouet, Deciphering key features in protein structures with the new ENDscript server. *Nucleic Acids Res.* **42**, W320–W324 (2014).
